# Selective Constraints on Amino Acids Estimated by a Mechanistic Codon Substitution Model with Multiple Nucleotide Changes

**DOI:** 10.1371/journal.pone.0017244

**Published:** 2011-03-18

**Authors:** Sanzo Miyazawa

**Affiliations:** Graduate School of Engineering, Gunma University, Kiryu, Gunma, Japan; Institute of Infectious Disease and Molecular Medicine, South Africa

## Abstract

**Background:**

Empirical substitution matrices represent the average tendencies of substitutions over various protein families by sacrificing gene-level resolution. We develop a codon-based model, in which mutational tendencies of codon, a genetic code, and the strength of selective constraints against amino acid replacements can be tailored to a given gene. First, selective constraints averaged over proteins are estimated by maximizing the likelihood of each 1-PAM matrix of empirical amino acid (JTT, WAG, and LG) and codon (KHG) substitution matrices. Then, selective constraints specific to given proteins are approximated as a linear function of those estimated from the empirical substitution matrices.

**Results:**

Akaike information criterion (AIC) values indicate that a model allowing multiple nucleotide changes fits the empirical substitution matrices significantly better. Also, the ML estimates of transition-transversion bias obtained from these empirical matrices are not so large as previously estimated. The selective constraints are characteristic of proteins rather than species. However, their relative strengths among amino acid pairs can be approximated not to depend very much on protein families but amino acid pairs, because the present model, in which selective constraints are approximated to be a linear function of those estimated from the JTT/WAG/LG/KHG matrices, can provide a good fit to other empirical substitution matrices including cpREV for chloroplast proteins and mtREV for vertebrate mitochondrial proteins.

**Conclusions/Significance:**

The present codon-based model with the ML estimates of selective constraints and with adjustable mutation rates of nucleotide would be useful as a simple substitution model in ML and Bayesian inferences of molecular phylogenetic trees, and enables us to obtain biologically meaningful information at both nucleotide and amino acid levels from codon and protein sequences.

## Introduction

Any method for inferring molecular phylogeny is implicitly or explicitly based on the evolutionary mechanism of nucleotide or amino acid substitutions, and the reliability of phylogenetic analyses strongly depends on models assumed for the substitution processes of nucleotide and amino acid. Mutational events occur at the individual nucleotide level, but selective pressure primarily operates at the amino acid level. Thus, a codon-based model of amino acid substitutions has a potential to be preferable to both mononucleotide substitution models [1]–[3] and amino acid substitution models [4]–[12], because it can take into account both mutational tendencies at the nucleotide level and selective pressure on amino acid replacements as well as the knowledge of a genetic code. Schneider et al. [13] and Kosiol et al. [14] empirically estimated a codon substitution matrix from a large number of coding sequence alignments. However, the tendencies of substitutions differ among nuclear, mitochondrial [6], and chloroplast genes [8]. Delport et al. [15], [16] pointed out that empirical substitution matrices represent the average tendencies of substitutions over various protein families by sacrificing gene-level resolution. A mechanistic codon substitution model, in which one can change a genetic code, and adjust mutational tendencies at the codon level and selectional preferences on amino acid replacements, is potentially more superior than empirical codon substitution matrices.

A main difference between the current mechanistic codon substitution models [7], [15]–[24] resides in the estimation of selective constraints against amino acid replacements. (1) In [19], [20], [22], the difference between nonsynonymous and synonymous substitution rates was taken into account but the amino acid dependences of selective constraints were not taken into account; i.e., single selective constraints. (2) In [7], [17], [18], selective constraints against amino acid replacements were evaluated from physico-chemical properties of amino acids. (3) In [21], [23], [24], codon exchangeabilities for nonsynonymous changes were evaluated from those in empirical amino acid substitution matrices. (4) In [15], [16], selective constraints were grouped, and the number of groups and the strength of selective constraint of each group were optimized for a given protein phylogeny. The fourth method has the highest resolution of selective constraints employing as many substitution groups as necessary. However, it seems to be a very computer-intensive calculation [16]. Here, we try to estimate selective constraint for each type of amino acid replacement by maximizing the likelihood of individual empirical substitution matrices. Unlike the present method, in the previous methods of this third category codon exchangeabilities for nonsynonymous changes were assumed to be proportional to the corresponding amino acid exchangeability [23], or a codon substitution matrix was restricted to yield amino acid exchangeabilities equal to empirically-derived ones [21]. The empirical substitution matrices fitted are 1-PAM amino acid substitution frequency matrices, the JTT matrix [5], the WAG matrix [10], and the LG matrix [11], evaluated from relatively large data of nuclear-encoded proteins, the mtREV matrix [6] from vertebrate mitochondrial proteins, and the cpREV matrix [8] from chloroplast-encoded proteins, and also a 1-PAM codon substitution frequency matrix (KHG) [14]. In the following, these empirical substitution frequency matrices corresponding to 1 PAM will be simply referred to by their common acronyms, JTT, WAG, LG, KHG, mtREV, and cpREV.

In most of the reversible Markov models for codon substitutions, instantaneous rates for codon substitutions that require multiple nucleotide changes were assumed to be equal to 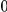. [15], [17]–[19]. However, in all empirical substitution matrices unnegligible amounts of rates are assigned to amino acid replacements that require multiple nucleotide changes. Variations in substitution rates or time intervals would yield significant amounts of probabilities for the multi-step substitutions. Alternative explanation is that the significant fraction of these substitutions occurred with multiple nucleotide changes. Thus, both of them are taken into account in the present work. It is assumed that substitution rates are distributed with a 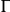 distribution. The use of 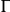 distribution for rate variation has been attempted in many studies [25], [26]. Multiple nucleotide changes are assumed to occur in the same order of time as single nucleotide changes do.

Interdependence of nucleotide substitutions at three codon positions [7] and also spanning codon boundaries [20] have been pointed out. Evidences for a high frequency, which is the order of 0.1 per site per billion years, of double-nucleotide substitutions were found in diverse organisms by Averof et al. [27], although there is a report [28] indicating a low rate of double-nucleotide mutations in primates. Bazykin et al. [29] pointed out a possibility of successive single compensatory substitutions for multiple nucleotide changes. Recently, many codon models relaxing mathematical assumptions in a more sophisticated way than the models of Goldman and Yang [18] and Muse and Gaut [19] are devised to study and to detect evidence of positive selection in codon evolutionary processes; see Anisimova and Kosiol [30] for a review.

In the Singlet-Doublet-Triplet (SDT) mutation model [20], single-nucleotide, doublet and triplet mutations spanning codon boundaries are taken into account, but double nucleotide mutations at the first and the third positions in a codon were not taken into account. The dependences of selective constraints on amino acid pairs were not taken into account. In the present model, it is assumed that nucleotide mutations occur independently at each codon position and so any double nucleotide mutation occurs as frequently as doublet mutations. The codon substitution rate matrix of KHG [14] indicates that some types of double nucleotide mutations at the first and the third positions frequently occur.

Close relationships between selective constraints on amino acids and physico-chemical properties of amino acids and protein structures have been pointed out [4],[9],[17],[31]–[34]. We suppose that the relative strengths of selective constraints among amino acid pairs do not strongly depend on species, organelles, and even protein families but amino acid pairs. Then, we examine the performance of the present codon-based model, in which selective constraints are approximated to be a linear function of those estimated from JTT, WAG, LG, or KHG, in respect of how well other empirical substitution matrices including cpREV and mtREV can be fitted by adjusting parameters such as mutational tendencies and the strength of selective constraints. It is shown that these maximum likelihood (ML) estimators of the selective constraints perform better than any physico-chemical estimation. It is also indicated that the present model yields good values of Akaike information criterion (AIC) for a phylogenetic tree of mitochondrial coding sequences in comparison with the codon model almost equivalent to mtREV. If the present model is applied to the ML inference of phylogenetic trees, it will allow us to estimate mutational tendencies at the nucleotide level, which are specific to each species and organelle, such as transition-transversion bias and the ratio of nonsynonymous to synonymous rate. One of the interesting results revealed by the present model is that the ML estimators of transition to transversion bias calculated from the empirical substitution matrices are not so large as previously estimated. Also, AIC values indicate that a model allowing multiple nucleotide changes fits the empirical substitution matrices and the phylogeny of vertebrate mitochondrial proteins significantly better.

The present codon-based model with the new estimates for selective constraints on amino acids is useful as a simple evolutionary model for phylogenetic estimation, and also useful to generate log-odds for codon substitutions in protein-coding sequences with any genetic code.

## Methods

### A mechanistic codon substitution model with multiple nucleotide changes

In early codon substitution models [17], [18], the probabilities of multiple nucleotide replacements in the infinitesimal time difference 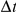 were completely neglected by assuming them to be 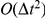, when the probabilities of single nucleotide replacements are taken to be 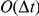. In other words, the instantaneous mutation rate 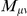 from codon 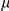 to 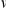 was assumed to be equal to zero for codon pairs requiring multiple nucleotide replacements. However, multiple nucleotide mutations may not be neglected in real protein evolution [7], [14], [20], [27], [29], [35]. Here, multiple nucleotide changes are assumed to occur with the same order of time as single nucleotide changes occur, but unlike the SDT model [20] a mutation process is simplified in such a way that mutations independently occur at each position of a codon. Thus, the mutation rate matrix for a codon is defined here as 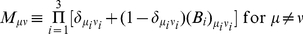
(1)


where 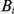 is a mutation rate matrix between the four types of nucleotides at the 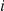
th codon position, 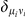 is the Kronecker's 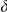, and the index 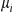 means the 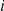
th nucleotide in the codon 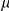; 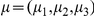 where 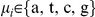. Assuming that the rate matrix 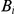 satisfies the detailed balance condition, it is represented as 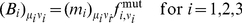
(2)

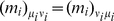
(3)

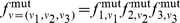
(4)


where 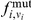 is the equilibrium composition of nucleotide 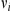 at the 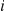
th codon position, and 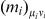 is the exchangeability between nucleotides 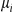 and 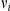 at the 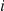
th codon position. As a result of the detailed balance condition assumed for the 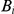, the 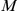 also satisfies the detailed balance condition; 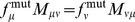
(5)


The instantaneous substitution rate 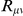 from codon 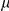 to 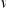 can be represented as the product of the mutation rate 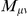 and the fixation probability 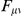 of the mutations under selection pressure; 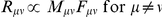. Let us assume that the 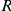 also satisfies the detailed balance condition; that is,
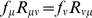
(6)


where 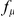 is the equilibrium codon composition of the substitution rate matrix 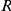. The detailed balance condition Eq. 6 for the 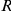 is equivalent with a condition that 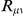 can be expressed to be a product of the 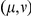 element of a symmetric matrix and the equilibrium composition 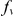. Similarly, the detailed balance condition Eq. 5 for the 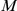 is equivalent with a condition that the matrix whose (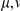) element is equal to 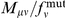 is symmetric. Thus, the detailed balance conditions for the 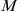 and the 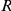 require that the fixation probability 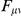 must be represented as the product of frequency-dependent, 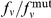, and frequency-independent, 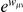, terms; 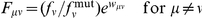, where 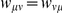. Then, the codon substitution rate 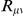 can be represented as 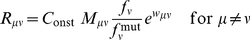
(7)


where 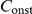 is an arbitrary scaling constant. The unit of time is chosen by determining the arbitrary scaling constant 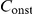 in Eq. 7 in such a way that the total rate of the rate matrix 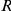 is equal to one; 
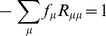
(8)


Therefore, only the relative values among 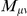 are meaningful. The frequency-dependent term 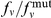 represents the effects of selection pressures at the DNA level as well as at the amino acid level, which preserve the codon frequency, 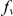, specific to a species and a protein, from the mutational frequency, 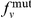. By taking the frequencies of stop codons to be zero, the rates from any codon to the termination codons are set to zero. The quantity 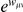 is the same as the one that Miyata et al. [32] called the rate of acceptance. We assume that selection pressure against codon replacements principally appears on an amino acid sequence encoded by a nucleotide sequence; 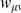 for the codon pair 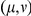 is equal to the selective constraint 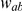 for the encoded amino acid pair 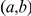.
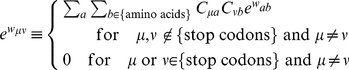
(9)


where 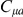 is a genetic code table and takes the value one if codon 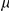 encodes amino acid 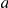, otherwise zero. At the amino acid level, there should be no selection pressure against synonymous mutations. Thus, the 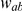 satisfies
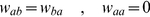
(10)


The matrix 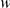 will be directly estimated by maximizing the likelihood of an empirical substitution matrix, or it will be evaluated for a specific protein family as a linear function of such an estimate of 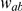;
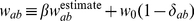
(11)


In Eq. 11, 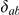 is the Kronecker's 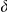, and 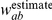 means the estimate of 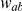, which is either a physico-chemical estimate or a ML estimate calculated from a specific substitution matrix, and satisfies Eq. 10. The parameter 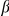, which is non-negative, adjusts the strength of selective constraints for a protein family. The parameter 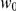 controls the ratio of nonsynonymous to synonymous substitution rate, but it will be ineffective and may be assumed to be equal to 0 if amino acid sequences rather than codon sequences are analyzed.

Then, the substitution probability matrix 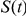 at time t in a time-homogeneous Markov process can be calculated as 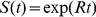
(12)


Because the rate matrix 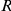 satisfies the detailed balance condition, the 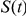 also satisfies it. Therefore, a substitution process is modeled as a reversible Markov process. The 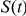 and the 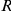 that satisfy the detailed balance condition can be easily diagonalized with real eigenvalues and eigenvectors [17]; the eigenvalues of 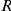 are the same as those of a symmetric matrix whose 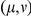 element is equal to 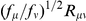.

If multiple nucleotide changes were completely ignored, then Eq. 1 would be simplified as 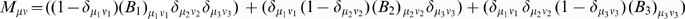, whose formulation for a codon mutation rate matrix with Eq. 2 is essentially the same as the one proposed by Muse and Gault [19]. Here, it should be noted that 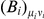 in Eq. 2 is defined to be proportional to the equilibrium nucleotide composition 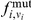. Alternatively, one may define 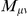 as 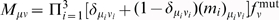 in the same way as Miyazawa and Jernigan [17] and others [7], [18] defined it to be proportional explicitly to the composition of the base triplet, 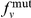. This alternative definition with Eqs. 7 and 8 is equivalent to Eqs. 1 and 2 with 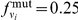, and thus it is a special case in the present formulation; see [36] for justifications of this alternative definition.

In the present analyses, we assume for simplicity that 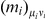 and 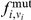 do not depend on codon position 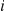; that is, 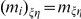 and 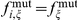, where 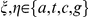. This assumption is reasonable because mutational tendencies may not depend on a nucleotide position in a codon. Let us define 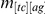 to represent the average of the exchangeabilities of the transversion type, 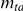, 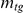, 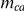, and 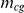, and likewise 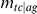 to represent the average of the exchangeabilities of the transition type, 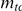 and 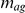. We use the ratios 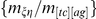 as parameters for exchangeabilities, and 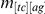 to represent the ratio of the exchangeability of double nucleotide change to that of single nucleotide change and also the ratio of the exchangeability of triple nucleotide change to that of double nucleotide change; note that the exchangeabilities of single, double, and triple nucleotide changes are of 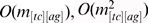, and 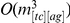 in Eq. 1, respectively, and that Eq. 8 must be satisfied. Then, multiple nucleotide changes in a codon can be completely neglected by making the parameter 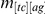 approach zero with keeping 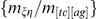 constant in Eq. 8. Also, it is noted that double nucleotide changes at the first and the third positions in a codon are assumed to occur as frequently as doublet changes.

### Empirical substitution matrices used for model fitting

Maximum likelihood (ML) values are calculated for each 1-PAM substitution frequency matrix, which corresponds to the time duration of 1 amino acid substitution per 100 amino acids, of the JTT [5], the WAG [10], the LG [11], the cpREV [8], and the mtREV [6] amino acid substitution matrices, and of the KHG codon substitution matrix [14]. We have arbitrarily chosen the transition matrices of 1-PAM, whose time interval is long enough for the significant number of substitutions to occur and also too short for multi-step substitutions to cover multiple nucleotide changes. JTT is an accepted point mutation matrix compiled from the pairs of closely related proteins encoded in nuclear DNA. WAG, LG, cpREV, and mtREV are amino acid substitution matrices estimated by maximizing the likelihood of a given set of optimum phylogenetic trees. The KHG matrix used is the one named ECMunrest in the supplement of their paper, for which multiple nucleotide changes are allowed. JTT, WAG, LG, and KHG were all calculated from nuclear-encoded proteins, although JTT was calculated by a different method from the others. The matrices of cpREV and mtREV were calculated from proteins encoded in chloroplast DNA, and in vertebrate mitochondrial DNA, respectively. It should be noted here that a non-universal genetic code is used in the mitochondrial DNA.

### Average of a transition matrix over time or over rate

In the present study, model parameters are estimated by maximizing the likelihood of each 1-PAM substitution frequency matrix of JTT, WAG, LG, cpREV, mtREV, and KHG. In the case of JTT, the pairs of closely related sequences were used to count substitutions and the transition matrix was calculated by completely neglecting multiple substitutions at a site in a parsimony method. Thus, JTT should be considered to consist of substitutions that occurred in various time intervals (various branch lengths). The substitution rate matrices of WAG, LG, mtREV, cpREV and KHG were estimated by the ML method for a given set of protein phylogenetic trees. Each site of protein families may have evolved with a different rate. As a result, these substitution matrices may be regarded as an average over different substitution rates. Here we assume that evolutionary time intervals or substitution rates for each substitution matrix are distributed in a 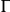 distribution. There have been many attempts [25], [26] of using a 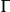 distribution for rate variation.

If the substitution rate matrix 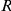 is assumed to vary only by a scalar factor, the mean of a substitution matrix irrespective of over-time and over-rate will be calculated as 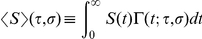


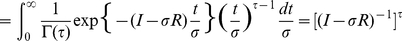
(13)


where 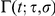 is the probability density function of a 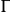 distribution with a scale parameter 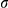 and a shape parameter 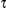, 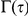 is the 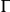 function, and 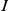 is the identity matrix. The mean and the variance of the 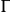 distribution 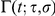 are equal to 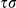 and 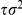, respectively. Here we should recall that the rate matrix 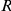 is normalized such that the total rate per unit time is equal to one; see Eq. 8.

### Evaluation of the log-likelihood of an empirical substitution matrix

The log-likelihood of the empirical frequency, 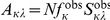, of substitutions from 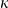 to 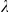 in the present model can be calculated as 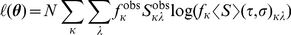
(14)


where 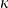 and 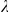 mean one of the amino acid types for amino acid substitution matrices or one of the codon types for codon substitution matrices, 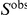 is an observed transition probability matrix corresponding to the accepted point mutation matrix 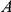, 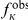 is the observed composition of amino acid or codon 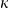, and 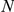 is the total number of amino acid or codon sites compared to count substitutions. The observed composition 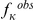 is assumed to be the equilibrium composition of 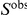. 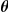 is a set of parameters and 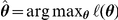 is a set of the maximum likelihood (ML) estimators. Similarly, the estimate 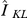 of the Kullback-Leibler (K-L) information by replacing the real distribution to the observed frequency distribution is calculated as 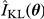


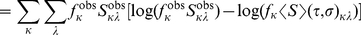
(15)

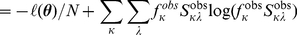
(16)


Maximum log-likelihood 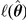 corresponds to the minimum of the estimate of K-L information, 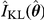.

The transition probability, 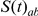, between amino acids 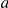 and 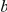 and the composition, 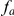, of amino acid 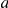 are related to those for codons as follows. 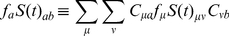
(17)

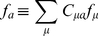
(18)


The goodness of a model and the significance of parameters can be indicated by Akaike Information Criterion (AIC). The AIC value is defined as 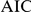


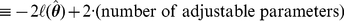
(19)

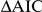


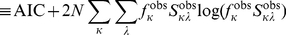
(20)

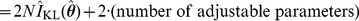
(21)


For convenience, 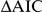, which is equal to a constant value added to the AIC value, is also defined above. The AIC and 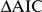 always take a non-negative value. Models with smaller AIC and 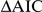 can be considered to be more appropriate [37].

Parameters in the present model are 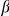, 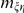, 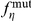, 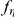, 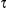, and 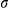. Assuming that the observed process of substitutions is in the stationary state, the estimates of the equilibrium codon and the equilibrium amino acid compositions, 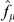 and 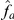, are taken to be the observed composition of the codon and of the amino acid: 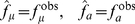
(22)


In the case of amino acid sequences, for which their coding sequences are not available, codon compositions may be parameterized by 
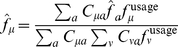
(23)

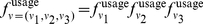
(24)


In the present analyses, this parameterization is used for the equilibrium codon compositions in amino acid sequences.

Then, the shape parameter 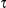 of a 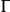 distribution for variations in mutation rates or evolutionary time intervals for observed codon or amino acid substitutions is estimated by equating the ratio of the expected number of substitutions in the model to its observed value.
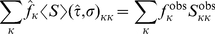
(25)


Other parameters 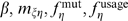, 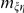, 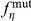, 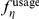, and 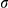 are evaluated as ML estimators or fixed to a proper value. The observed transition matrix 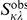 corresponding to 1-PAM is used here; PAM means accepted point mutations per 100 amino acids. 
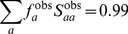
(26)


### The total number of site comparisons (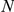) for each empirical substitution matrix

In the case of JTT, 59190 accepted point mutations found in 16130 protein sequences were used to build a substitution probability matrix of 1-PAM [5]. Thus, the total number 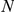 of amino acid comparisons for JTT is assumed to be equal to 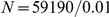. On the other hand, a phylogenetic tree for cpREV is based on 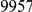 amino acid sites of 45 proteins encoded in chloroplast DNAs of 9 species [8], and the one for mtREV is based on 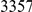 amino acid sites of the complete mitochondrial DNA from 20 vertebrate species (3 individuals from human) [6]. Thus, the total number of site comparisons 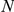 for them may be approximated to be equal to the number of amino acid sites multiplied by the number of branches in the phylogenetic tree used to evaluate the transition matrices; that is, 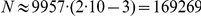 for cpREV, and 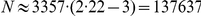 for mtREV. The BRKALN database consisting of 50867 sites and 895132 residues was used to estimate WAG. Thus, 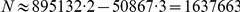 is used for WAG [10], [11]. To evaluate LG, 3412 of 3912 alignments consisting of 49637 sequences, 599692 sites, and 6697813 residues are used [11]. Therefore, 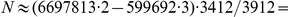 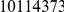 is assumed for LG. These crude estimates of 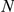 are used to evaluate the AICs of JTT, WAG, LG, cpREV and mtREV.

In the case of KHG, which was estimated by maximizing a likelihood of a set of phylogenetic trees of coding sequences of 7332 nuclear protein families taken from Pandit database [38], the total numbers of residues and sites are not written in Kosiol et al. [14], so that an AIC value is not given for KHG in the following.

## Results

Models, each of which includes a different number of parameters and is a special case of models including more parameters, are fitted by a maximum likelihood method to each of the 1-PAM amino acid substitution frequency matrices, JTT [5], WAG [10], and LG [11] for proteins encoded in nuclear DNA, cpREV [8] for chloroplast DNA, and mtREV [6] for mitochondrial DNA. Also, the models are fitted to the 1-PAM codon substitution frequency matrix of KHG [14] for nuclear DNA. The selective constraints 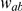 are either directly estimated by ML or evaluated from a known estimate 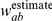 by Eq. 11 that includes two parameters 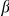 and 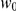. The parameter 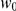 is fixed here to 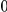 for amino acid substitution matrices because the likelihood of an amino acid substitution matrix does not strongly depend on 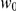; codon substitution data are required to reliably estimate the value of 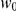, which significantly affects the ratio of nonsynonymous to synonymous substitution rate. Each model is named to indicate either the method to estimate 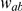 or the name of 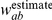 with a suffix meaning the number of ML parameters. Each model is briefly described in Table 1. The Nelder-Mead Simplex algorithm has been used for the maximization of likelihoods.

**Table 1:** Brief description of models.

Model name	Description
No-Constraints- 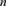	No amino acid dependences of selective constraints; 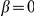 . The suffix 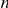 means the number of ML parameters.
EI- 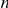	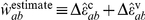 based on the Energy-Increment-based (EI) method, which is described in Text S1, is used to estimate 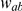 in Eq. 11. The suffix 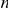 means the number of ML parameters.
Miyata- 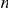	The amino acid pair distance 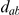 estimated by Miyata et al. [32] is used as 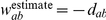 to estimate 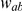 in Eq. 11. The suffix 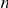 means the number of ML parameters.
Grantham- 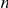	The amino acid distance 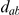 estimated by Grantham [31] is used as 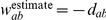 to estimate 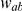 in Eq. 11. The suffix 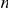 means the number of ML parameters.
ML- 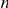	Selective constraints 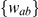 are estimated by maximizing the likelihood of JTT [5], WAG [10], or LG [11], and called 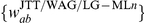 . The suffix 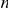 means the number of ML parameters. In the ML-87, multiple nucleotide changes are disallowed, and 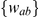 for all 75 single-step amino acid pairs are estimated. In the ML-91 and the ML-94, multiple nucleotide changes are allowed, and 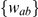 for all 75 single-step amino acid pairs and for 6 groups of multiple-step amino acid pairs are estimated. In the ML-91, equal codon usage is assumed. In the ML-200 for codon substitution matrices, 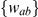 for all 190 amino acid pairs are estimated.
ML- 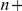	First, the ML- 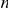 is used to estimate parameters, and then 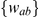 for all multiple-step amino acid pairs are estimated by maximizing the likelihood with fixing all other parameters to the values estimated by the ML- 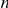 .
JTT-ML91- 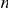 , WAG-ML91- 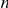 , LG-ML91- 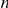	Selective constraints 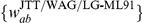 estimated by maximizing the likelihood of JTT/WAG/LG [5], [10], [11] in the ML-91 model are used as 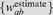 in Eq. 11. The suffix 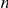 means the number of ML parameters.
JTT-ML91+− 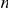 , WAG-ML91+− 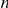 , LG-ML91+− 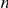	Selective constraints 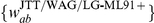 estimated by maximizing the likelihood of JTT/WAG/LG [5], [10], [11] in the ML-91+ model are used as 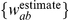 in Eq. 11. The suffix 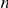 means the number of ML parameters. The JTT/WAG/LG-ML91+−0 models correspond to the JTT/WAG/LG-F models, respectively.
KHG-ML200- 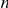	Selective constraints 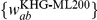 estimated by maximizing the likelihood of the KHG codon substitution matrix [14] in the ML-200 model are used as 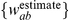 in Eq. 11. The suffix 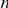 means the number of ML parameters. The KHG-ML200-0 models correspond to the KHG-F model.

### The effects of selective constraints

First, the No-Constraints models, in which selective constraints do not depend on amino acid pairs, 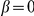 in Eq. 11, were examined to see how well nucleotide mutation rates, codon frequencies and a genetic code can explain the observed frequencies of amino acid substitutions in JTT, WAG, cpREV, and mtREV; the No-Constraints models disallowing multiple nucleotide changes are equivalent to mononucleotide substitution models, because 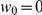 is used here. The 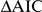 value and the ML estimates for each parameter set are listed in Table 2 and Table S1, respectively. Please refer to Text S1 for details. These No-Constraints models serve as a reference to measure how selection models can improve the likelihoods. Then, we examine various estimations of selective constraints on amino acids based on the physico-chemical distances of amino acids evaluated by Grantham [31] and by Miyata et al. [32] and mean energy increments due to an amino acid substitution. These models are called Grantham, Miyata, and Energy-Increment-based (EI) models, respectively. Please refer to Text S1 for the definition of the mean energy increment and for the details of each model. The 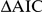 values and the ML estimates for these models with various sets of parameters are also listed in Table 2, and Tables S2 and S3, respectively. Comparisons of 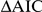 values between the models in Table 2 indicate that the selective constraints on amino acids representing conservative selection against amino acid substitutions significantly improve the 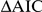 values of all substitution matrices. It is also indicated that the Miyata's physico-chemical distance performs better in all parameter sets than the Grantham's distance, This result is consistent with that of Yang et al. [7] for mitochondrial proteins. The present physico-chemical evaluation of selective constraints (EI models) fits JTT and WAG even better than the Miyata's distance scale, although the performances of both the methods are almost same for cpREV and mtREV. One of the important facts in these results is that allowing multiple nucleotide changes in a codon significantly improve the AIC irrespective of the estimations of selective constraints; compare the 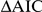 values between the Grantham-10 and the Grantham-11, between the Miyata-10 and the Miyata-11, and between the EI-10 and the EI-11.

**Table 2:** 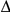
 AICvalues of the present models without and with the selective constraints on amino acids, which are based on mean energy increments due to an amino acid substitution (EI), the Miyata's and the Grantham's physico-chemical distances, for the 1-PAM amino acid substitution matrices of JTT, WAG, cpREV, and mtREV.

		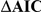 a			
Model	#parameters	JTT	WAG	cpREV	mtREV
	(id no.^b^)				
No-Constraints-					
1	21( 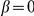 , 3)	86428.1	37917.6	3478.0	2644.1
10	30( 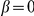 , 2–10,14)	24595.6	7719.1	904.5	901.0
13	33( 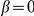 , 2–14)	22913.6	7141.5	874.9	798.8
EI-					
2	22(1,3)	77337.9	35058.8	3186.0	2396.6
2G	22(1,14)	24197.7	5571.6	974.0	1066.8
3	23(1,3,14)	16463.7	4995.0	761.5	776.4
4	24(1–3,14)	15808.7	4443.6	743.0	753.9
8	28(1–7,14)	15715.0	4327.8	722.0	728.2
7	27(1–3,8–10,14)	15081.0	4312.6	650.7	688.7
10	30(1,3–10,14)	15435.7	4801.8	670.7	702.8
10M	30(1–10)	15270.7	4250.4	645.3	674.3
11	31(1–10,14)	14999.0	4202.5	636.0	674.3
10MU	30(1–3,8–14)	13464.3	3959.7	578.9	662.4
12	32(1,3–13)	72316.3	33908.4	2939.7	2215.0
13	33(1,3–14)	13819.7	4554.2	623.6	655.5
13M	33(1–13)	13436.2	3822.4	551.1	623.3
14	34(1–14)	13151.9	3748.0	541.9	614.8
Miyata-					
4	24(1–3,14)	16090.1	4938.1	750.3	783.0
7	27(1–3,8–10,14)	15767.2	4715.4	654.5	701.6
10	30(1,3–10,14)	16446.1	5124.9	679.2	708.5
11	31(1–10,14)	15536.8	4429.5	628.4	658.4
13	33(1,3–14)	15058.2	4943.1	656.5	682.3
14	34(1–14)	14338.5	4254.0	603.7	613.6
Grantham-					
4	24(1–3,14)	20505.1	5953.7	916.4	887.1
7	27(1–3,8–10,14)	18898.2	5814.0	840.6	832.9
10	30(1,3–10,14)	18744.5	5749.0	805.4	799.8
11	31(1–10,14)	18680.9	5579.7	803.2	796.5
13	33(1,3–14)	16784.9	5512.9	765.0	741.0
14	34(1–14)	16729.7	5477.1	755.0	739.5

^*a*^ 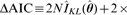 #parameters with 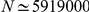 for JTT, 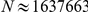 for WAG, 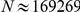 for cpREV, and 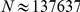 for mtREV; see text for details.

^*b*^ML parameters in each model are specified by the parameter id numbers in the parenthesis, and other parameters are fixed at 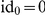, 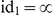, 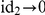, 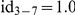, 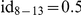, and 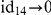. Each id number corresponds to the parameter id number listed in Table 3.

**Table 3:** ML estimates and 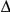
AIC values of the present models for the 1-PAM amino acid substitution matrices of JTT, WAG, and LG, and the 1-PAM codon substitution matrix of KHG.

		JTT			WAG			LG		KHG
										(codon)
id	parameter	ML–87^a^	ML–91^a^	ML–94	ML–87^a^	ML–91^a^	ML–94	ML–91^a^	ML–94	ML–200
no.										
0	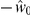	N/A	N/A	N/A	N/A	N/A	N/A	N/A	N/A	N/A
1	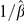	N/A	N/A	N/A	N/A	N/A	N/A	N/A	N/A	N/A
2	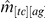	( 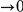 )	0.637	0.662	( 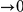 )	1.28	1.29	1.08	1.19	0.939
3	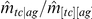	0.0919	1.57	1.59	0.746	1.70	1.69	1.85	1.81	0.843
4	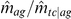	1.77	1.14	1.15	1.98	1.32	1.31	1.23	1.21	0.945
5	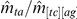	0.0293	0.729	0.730	0.0477	0.791	0.784	0.676	0.682	1.52
6	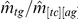	3.21	0.940	0.950	3.64	1.04	1.01	1.07	1.07	0.554
7	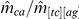	0.719	1.19	1.18	0.110	1.23	1.23	1.28	1.25	0.573
8	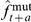	0.408	0.459	0.446	0.372	0.367	0.392	0.388	0.403	0.497
9	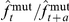	0.113	0.501	0.522	0.234	0.587	0.513	0.450	0.439	0.513
10	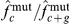	0.698	0.429	0.436	0.425	0.479	0.471	0.427	0.383	0.470
11	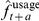	0.0682	(0.5)	0.483	0.0669	(0.5)	0.221	(0.5)	0.447	NA
12	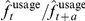	0.461	(0.5)	0.491	0.330	(0.5)	0.429	(0.5)	0.555	NA
13	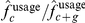	0.386	(0.5)	0.558	0.310	(0.5)	0.306	(0.5)	0.249	NA
14	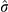	27.3	0.738	0.740	43.3	0.905	0.840	0.415	0.395	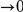
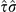		0.334	0.0243	0.0246	0.317	0.0223	0.0207	0.0246	0.0240	0.0240
#parameters		107	111	114	107	111	114	111	114	261
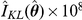 b		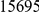	638	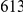	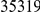	1903	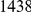	2771	2335	269946
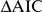 c		2072.0	297.5	300.6	1370.8	284.3	275.1	782.5	700.4	unknown
Ratio of substitution rates										
per codon										
the total base/codon		1.28	1.35	1.35	1.38	1.53	1.52	1.38	1.39	1.29
										(1.29)^d^
transition/transversion		0.464	1.08	1.08	0.482	0.932	0.806	1.18	1.20	0.764
										(0.765)^d^
nonsynonymous/synonymous^e^		1.13	1.37	1.34	1.57	2.07	2.40	1.05	1.20	0.726
										(0.723)^d^
Ratio of substitution rates										
per codon for 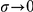										
total base/codon		1.0	1.22	1.22	1.0	1.38	1.40	1.31	1.33	1.29
transition/transversion		0.101	1.21	1.22	0.647	1.11	0.932	1.31	1.35	0.764
nonsynonymous/synonymous^e^		0.0644	1.04	1.02	0.138	1.50	1.79	0.853	0.889	0.726
Ratio of substitution rates per										
codon for 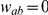 and 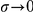										
total base/codon		1.0	1.45	1.46	1.0	1.72	1.74	1.67	1.71	1.51
transition/transversion		0.0605	0.829	0.831	0.499	0.933	0.849	0.992	0.981	0.427
nonsynonymous/synonymous^e^		11.3	5.58	5.74	11.1	8.68	11.1	7.45	8.46	6.81

^*a*^If the value of a parameter is parenthesized, the parameter is not variable but fixed to the value specified.

^*b*^ 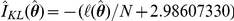 for JTT, 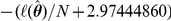 for WAG, 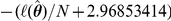 for LG, and 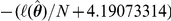 for KHG; see text for details.

^*c*^ 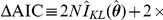 #parameters with 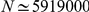 for JTT, 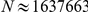 for WAG, 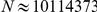 for LG, and the value of 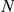 is unknown for KHG; see text for details.

^*d*^The value in the parenthesis corresponds to the one for the KHG codon substitution probability matrix.

^*e*^Note that these ratios are not the ratios of the rates per site but per codon; see text for details.

### The effects of multiple nucleotide changes on ML estimations

In principle, all parameters 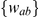 for selective constraints can be optimized in the case of codon sequences. In the case of protein sequences, all 190 non-diagonal elements of 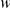 in addition to the parameters for mutational tendencies at the nucleotide level and others cannot simultaneously be optimized; the number of freedoms in a general reversible model for an amino acid transition matrix is equal to 209.

In order to see how well amino acid substitution matrices can be explained with the assumption of successive single nucleotide substitutions, let us optimize 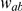 corresponding to single-step amino acid pairs by assuming that only single nucleotide mutations are possible, i.e., by 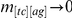 with 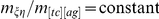 in Eq. 8. The number of 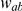 for the single-step amino acid pairs is equal to 75 in the case of the universal genetic code. All 75 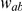 for the single-step amino acid pairs have been optimized for each of JTT and WAG together with the nucleotide exchangeabilities 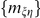, the equilibrium nucleotide composition 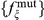, the codon usage parameters 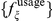 and the scale parameter 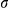; the total number of the parameters is equal to 87 in addition to the 19 amino acid frequencies and the shape parameter 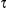. This maximum likelihood model to estimate the matrix 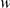 is called ML with a suffix meaning the number of ML parameters; see Table 1. The ML estimates of these parameters except 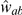 for the ML-87 are listed in Table 3 for JTT and WAG.

In the lowest rows of this table, the ratio of the total nucleotide substitution rate per codon to the codon substitution rate, which represents the average number of nucleotide changes for substituting a codon, the ratio of the total transition to the total transversion rate per codon, and the ratio of nonsynonymous to synonymous substitution rate per codon are listed for the models. The sum of the total transition and the total transversion rates per codon is equal to the total nucleotide substitution rate per codon. The lowest three rows list their values in the case of 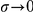 and 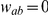, and the second lowest three rows for the case of 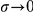. Thus, the differences of their values between the lowest and second lowest three rows represent the effects of selective constraints on amino acids (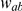), and those between the second lowest and the third lowest three rows describe the effects of rate/time variations on the substitution matrix. If codon substitutions proceed by successive single nucleotide changes, i.e., 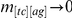, then the ratio of the total nucleotide to the codon substitution rate will be equal to 1 in the case of 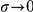.

Here it should be noticed that the nonsynonymous and the synonymous substitution rates are defined not to be rate per site but simply rate per codon. The sum of the nonsynonymous and the synonymous substitution rates is equal to the codon substitution rate. The ratio of the nonsynonymous to the synonymous substitution rate per codon does not corresponds to the ratio of nonsynonymous to synonymous substitutions per site, 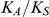 [39], but the ratio of nonsynonymous to synonymous substitutions per codon, 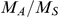 [39]. The ratio (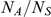 [39]) of the effective number of nonsynonymous sites to that of synonymous sites per codon corresponds to the ratio of nonsynonymous to synonymous rate in the case of no selective constraints (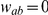). In the present models, 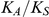 indicating the effects of selection on amino acid replacements corresponds to the nonsynonymous to synonymous substitution rate ratio in the case of 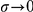 divided by that in the case of 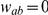 and 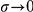. Table 3 indicates that selection on amino acids is conservative, because the ratio of nonsynonymous to synonymous rate per codon is much smaller in the case of 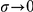 than in the case of 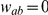 and 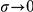.

As expected, the AIC value drastically decreases from that of the EI-14 in both cases of JTT and WAG, indicating that the introduction of many parameters may be still appropriate. However, there are large discrepancies between the observed transition matrix and the one estimated by the ML-87. Let us see the discrepancies between them in terms of log-odds.

A log-odds matrix introduced by Dayhoff et al. [4] is one of the representations of amino acid substitution propensities. The 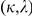 element of the log-odds matrix is defined to be the logarithm of odds to find an amino acid pair 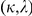 in comparison with random sequences. The odds 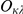 is equal to the 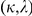 element of transition matrix divided by the amino acid composition 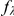.
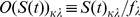
(27)

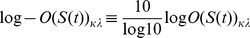
(28)


The proportional constant in Eq. 28 is the one originally used by Dayhoff et al. [4].

**Figure 1:**
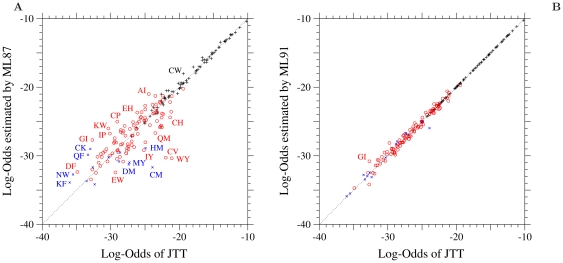
The ML-87 and the ML-91 models fitted to JTT. Each element log-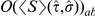 of the log-odds matrices of (A) the ML-87 and (B) the ML-91 models fitted to the 1-PAM JTT matrix is plotted against the log-odds log-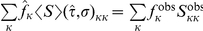 calculated from JTT. Plus, circle, and cross marks show the log-odds values for the types of substitutions requiring single, double and triple nucleotide changes, respectively. The dotted line in each figure shows the line of equal values between the ordinate and the abscissa.

In Fig. 1, the log-odds 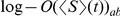 corresponding to the 1 PAM transition matrix of the ML-87 model fitted to JTT are plotted against those calculated from JTT. Plus, circle and cross marks show the log-odds for one-, two-, and three-step amino acid pairs, respectively. Although the estimated values of log-odds for one-step amino acid pairs are almost exactly equal to those of the JTT matrix, there are still large discrepancies between the log-odds values for two- and three-step amino acid pairs, indicating a non-stepwise manner of codon substitutions. Similar discrepancies are also found in Fig. S1 for WAG.

We have examined how the AIC is improved by enabling multiple nucleotide changes in a codon. The selective constraints 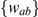 for multiple nucleotide changes are classified into 6 groups according to the amounts of discrepancies between the observed and the estimated values of the log-odds as shown in Fig. 1. Then, the ML estimates of 94 parameters including 7 additional parameters, 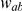 for the 6 groups of multiple nucleotide changes and the parameter 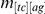 for the rate of multiple nucleotide change, are calculated. This model is called ML-94. Also, the values of 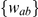 for multi-step amino acid pairs are calculated by maximizing the likelihood with fixing the values of all other parameters including 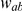 for the single-step amino acid pairs; this model is called here ML-94+ by appending the "+" mark. It should be noted that these values of 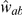 for the multi-step amino acid pairs in the ML-94+ are not ML estimates at all. The ML estimates 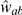 for single-step amino acid pairs, the classification of multi-step amino acid pairs into the 6 groups, and the ML estimates for those categories of 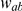 are provided in Data S1. As shown in Table 3, the ML estimates of 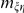, 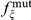, and 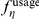 for the ML-87 model are very different from those for the ML-94, and some of them for the ML-87 seem to be unrealistic. For example, 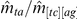 is evaluated to be smaller than 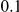. Also, the small value of 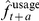 indicates the extremely biased usage of codons. The ML estimate 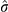 of a 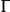 distribution is too large. These parameters are forced in the ML-87 to take such values to reduce the discrepancies between the observed and the estimated counts for multi-step amino acid pairs. In the ML-94 model, the ML estimators of these parameters take more reasonable values. However, it may also yield unreasonable estimates for codon usage parameters, 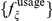; for example, 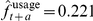 in the ML-94 for WAG, and 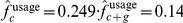 in the ML-94 for LG. Thus, the ML-91 model with 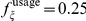, which means equal codon usage, may be better than the ML-94. The ML-91 model was applied for JTT, WAG, and LG, and the ML estimates for them in the ML-91 are also listed in Table 3.

The ML estimators 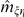, 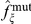, and 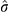 show a similar tendency between the ML-91 models for all the amino acid substitution matrices, i.e., JTT, WAG, and LG. The parameter 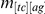 for multiple nucleotide changes and the scale parameter 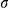 for rate variation are both significant for all the matrices. The values of 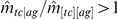 for JTT, WAG, and LG indicate that the mean exchangeability of the transition type is larger than that of the transversion type in all the matrices.

As shown in Fig. 1 for JTT and in Fig. S1 for WAG, the large discrepancies of the log-odds for the multi-step amino acid pairs disappear in the ML-91, in which multiple nucleotide changes are taken into account. The AIC values of JTT and WAG are significantly improved by enabling multiple nucleotide changes in the ML-91. This fact confirms that multiple nucleotide changes are statistically significant and should be taken into account to build a codon substitution model.

### ML estimation for the KHG codon substitution matrix

If a codon substitution matrix is used for model fitting with the assumption of multiple nucleotide changes, all 190 parameters of selective constraints 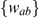 will be able to be optimized. The ML-200 model has been fitted to the 1-PAM codon substitution frequency matrix of KHG, which was empirically estimated without any restriction on multiple nucleotide changes [14].

**Figure 2:**
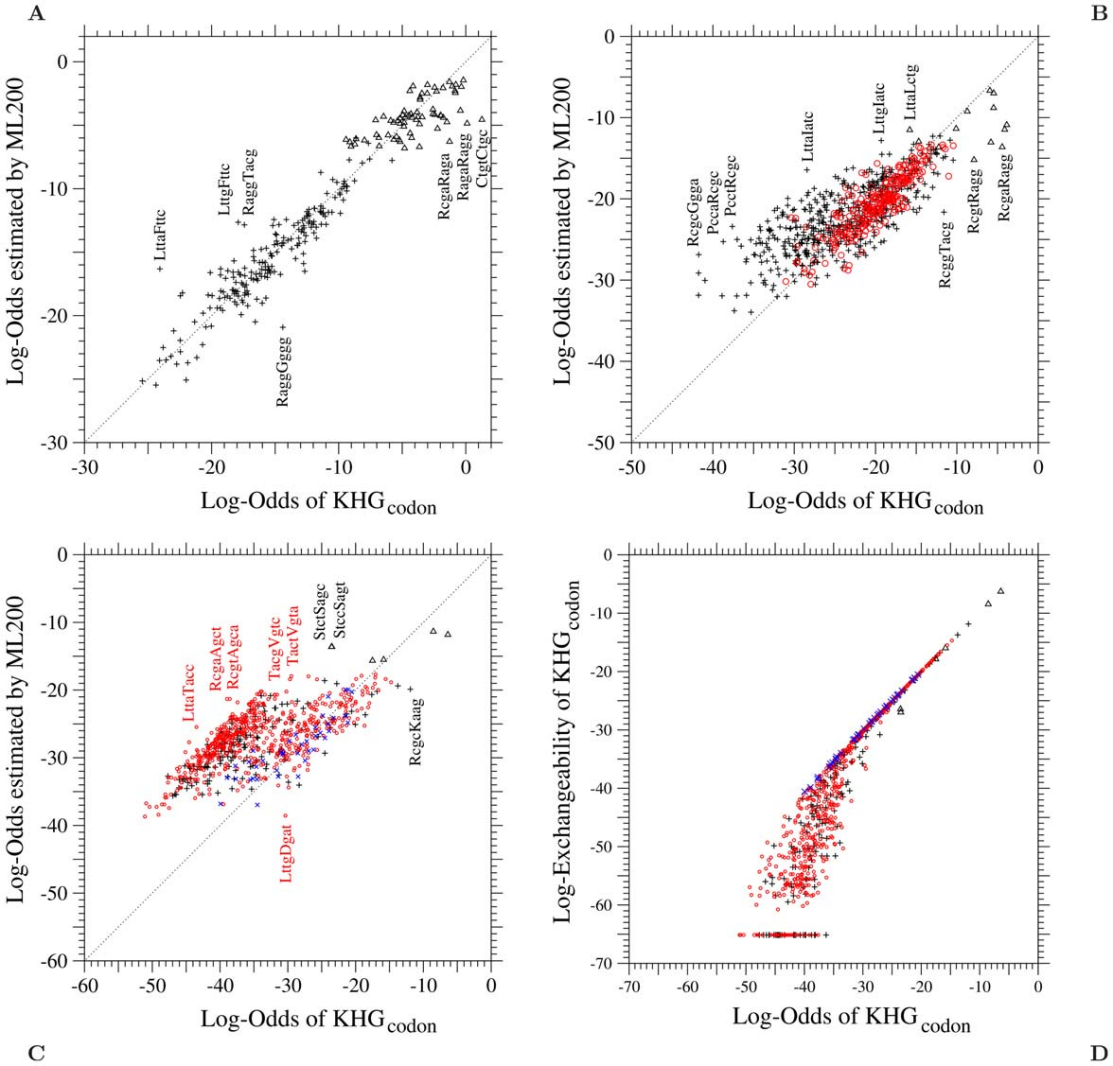
The ML-200 model fitted to KHG. Each element log-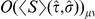 of the log-odds matrix corresponding to (A) single, (B) double, and (C) triple nucleotide changes in the ML-200 model fitted to the 1-PAM KHG codon substitution matrix is plotted against the log-odds log-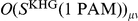 calculated from KHG. In (D), codon log-exchangeabilities of the 1-PAM KHG codon substitution matrix corresponding to triple nucleotide changes are plotted against the log-odds log-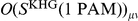 calculated from KHG. The log-exchangeability of the 1-PAM KHG is defined as 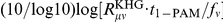. Upper triangle, plus, circle, and cross marks show the log-odds values for synonymous pairs and one-, two-, and three-step amino acid pairs, respectively. Log-exchangeabilities for the codon pairs whose instantaneous rates are estimated to be 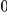 in KHG are shown to be about 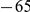 in this figure. The dotted line in each figure shows the line of equal values between the ordinate and the abscissa.

The log-odds values for the codon pairs requiring single, double, and triple nucleotide changes are shown in Figs. 2A, 2B, and 2C, respectively. In these figures, upper triangle, plus, circle, and cross marks show the log-odds values for synonymous pairs and one-, two-, and three-step amino acid pairs, respectively. The dotted line shows the line of values where the observed and the estimated values of log-odds are equal to each other. The log-odds of the codon pairs requiring single/double/triple nucleotide changes for one/two/three-step amino acid pairs respectively tend to fall along the dotted line in comparison with the log-odds of the other codon pairs. In other words, the log-odds of the codon pairs for which any nucleotide change is accompanied by an amino acid change are correctly estimated. On the other hand, the estimated log-odds values do not well agree with the observed ones for synonymous codon pairs shown by the upper triangles. These estimated log-odds can be adjusted only by changing nucleotide mutation rates, i.e., 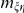 and 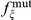. Thus, the approximations of the independence and of no difference of nucleotide exchangeabilities between nucleotide positions may be limited; see Eq. 1.

The codon pairs, whose log-odds values are less than 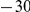 and which require more nucleotide changes than the least nucleotide changes required for the corresponding amino acid pair, tend to be located in the upper region than in the lower region of the dotted line; see plus marks in Fig. 2B and plus and circle marks in Fig. 2C. Such a tendency is more clear in Fig. 2C, in which plus and circle marks corresponding to one- and two-step amino acid pairs are mostly located far from and almost in parallel to the dotted line. The estimated values of the log-odds for these one- and two-step amino acid pairs are greater by 10 – 15 than the observed values.

In Fig. 2D, the log-exchangeabilities of the codon pairs requiring triple nucleotide changes in the 1-PAM KHG matrix are plotted against their log-odds of the 1-PAM KHG matrix. The log-exchangeability is defined here to be 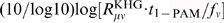. The log-exchangeabilities of the codon pairs corresponding to three-step amino acid pairs are all nearly equal to their log-odds. The smallest log-exchangeabilities of these codon pairs reach almost 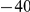. However, there are many codon pairs whose log-exchangeabilities are smaller than 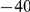, and all of them correspond to one- or two-step amino acid pairs. The log-exchangeabilities of these codon pairs are significantly smaller than their log-odds, indicating that almost all substitutions of these codon pairs were estimated in KHG not to occur by triple nucleotide changes but rather by successive single or double nucleotide changes.

In the present model, codon exchangeabilities are approximated by the product of nucleotide exchangeabilities; see Eq. 1 for the exact expression. Therefore, all codon exchangeabilities for triple nucleotide changes are in the same order of magnitude, and specific codon pairs cannot be significantly less exchangeable. Thus, the present approximation for codon exchangeabilities may have a limitation, unless those exchangeabilities of KHG are underestimated. Estimation of the exchangeabilities for those codon pairs, which require more nucleotide changes than the least nucleotide changes required for the corresponding amino acid pair, may be less reliable than for the others.

The ML estimates 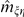, 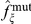 and 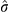 for KHG are listed in Table 3. The scale parameter 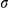 of the 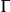 distribution is estimated to be 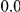 for KHG, meaning that variations in rates need not be taken into account for KHG. There is a different tendency in the 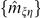 between KHG and the amino acid substitution matrices. One remarkable difference between them is that the parameter 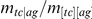 for transition-transversion bias is estimated to be greater than one in the ML-91 for JTT, WAG, and LG but to be less than one in the ML-200 for KHG. This estimation of transition to transversion bias for KHG results from a fact that the ratio of the total transition to the total transversion substitution rate is actually equal to 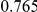 in KHG, although this fact is contrary to the common understanding of transition-transversion bias. Because selective constraints on amino acids more favor transitions than transversions, transition-transversion bias in nucleotide mutation rates for KHG must be much less than 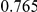. Actually the ratio of the total transition to the total transversion mutation rate is estimated to be 0.427; see Table 3.

### Comparison of ML estimates 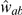 among the present models

In Table 4, the correlation coefficients of 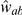 between the present models are listed. The lower half of the table lists those for single-step amino acid pairs, and the upper half lists those for multi-step amino acid pairs by excluding the amino acid pairs that belong to the least exchangeable class at least in one of the models. Each model name of JTT/WAG/LG-ML91+ and KHG-ML200 means the empirical substitution matrix and the method used to estimate selective constraints, 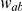. In the following, these ML estimates of 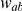 will be specified as 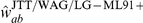 and 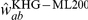. In the EI method, selective constraints are approximated by a linear function of the energy increment due to an amino acid substitution, 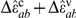, which is defined by Eqs. S1-4, S1-5, and S1-6 in Text S1; therefore, 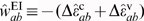.

**Table 4:** Correlations of 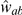 between various estimates; the lower half shows the correlation coefficients of 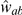 for 75 single-step amino acid pairs and the upper half does those of 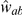 for 86 multi-step amino acid pairs by excluding 29 amino acid pairs of the least exchangeable category in the JTT-ML91, the WAG-ML91 or the LG-ML91.

Model	EI	JTT-ML91+	WAG-ML91+	LG-ML91+	KHG-ML200	
EI		0.45	0.51	0.59	0.55	(0.65)^a^
JTT-ML91+	0.66		0.80	0.80	0.51	
WAG-ML91+	0.68	0.87		0.86	0.55	
LG-ML91+	0.71	0.82	0.90		0.58	
KHG-ML200	0.71	0.77	0.69	0.74		

^*a*^The value in the parenthesis is the correlation coefficient for which the 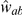 for all multi-step amino acid pairs are taken into account. The correlation coefficient of 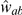 for all amino acid pairs between the EI and the KHG-ML200 is equal to 0.60.

The correlations of the ML estimates 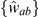 between the JTT-ML91+, the WAG-ML91+, and the LG-ML91+ are very strong even for the multi-step amino acid pairs. Comparisons of the ML estimates of selective constraints between various models are shown in Fig. S2. The 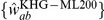 estimated from the KHG codon substitution matrix are less correlated with 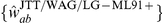 from the other amino acid substitution matrices, especially less for the multi-step amino acid pairs. The ML estimates 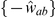 for the multi-step amino acid pairs are relatively smaller in the KHG-ML200 than in the JTT/WAG/LG-ML91+ models; see Fig. S2.

**Figure 3:**
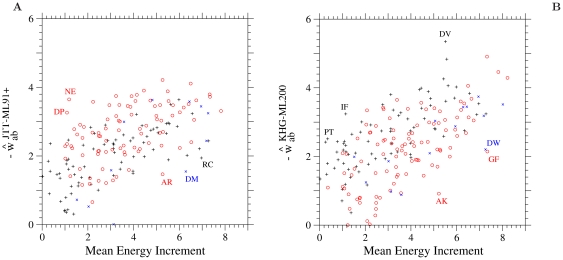
Selective constraint for each amino acid pair estimated from JTT and from KHG. The ML estimate, (A) 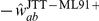 in the ML-91+ model fitted to the 1-PAM JTT amino acid substitution matrix and (B) 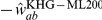 in the ML-200 model fitted to the 1-PAM KHG codon substitution matrix, for each amino acid pair is plotted against the mean energy increment due to an amino acid substitution, (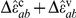) defined by Eqs. S1-4, S1-5, and S1-6 in Text S1. In (A), the estimates 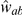 for the least exchangeable class of multi-step amino acid pairs are not shown. Plus, circle, and cross marks show the values for one-, two-, and three-step amino acid pairs, respectively.

The correlations of 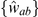 between the EI and others are not as good as those between the other estimates, but they are significant especially between the EI and the KHG-ML200 even for the multi-step amino acid pairs. In Fig. 3A, the ML estimates 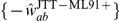 in the JTT-ML91+ are plotted against the energy increments 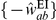 due to an amino acid substitution; the least exchangeable category of multi-step amino acid pairs are not shown in this figure. Similar plots for the WAG-ML91+ and for the LG-ML91+ are shown in Fig. S3. The ML estimates 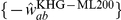 for all amino acid pairs in the KHG-ML200 are plotted against the energy increments 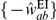 in Fig. 3B. No drastic difference in the correlation between these two quantities is found among one-, two-, and three-step amino acid pairs. The correlations of 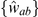 between the EI and the other models are better for the ML-91 than for the ML-87; the correlation coefficient between them for the single step amino acid pairs is equal to 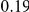 for the JTT-ML87 but 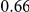 for the JTT-ML91 and 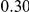 for the WAG-ML87 but 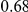 for the WAG-ML91. The ML estimates 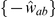 for the single step amino acid pairs are compared between the ML-87 and the ML-91 models in Fig. S4.

In the next section, we will examine whether the differences among these estimates of 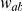 are significant in representing selective constraints on amino acids.

### Performance of the ML estimates 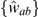 and the characteristics of nucleotide mutations estimated

The present model for codon substitutions is designed to separate selective pressures at the amino acid level from mutational events at the nucleotide level. Both unequal usage of degenerate codons and different rates of transition and transversion are characteristic of a genetic system specific to each species and each organelle. On the other hand, the relative strengths of selective constraints on amino acids would be far less specific to each species and each protein than each type of amino acid, although the mean strength of the selective constraints is specific to each protein family. Thus, we tried to approximate selective constraints (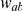) for empirical substitution matrices including cpREV and mtREV by a linear function of those (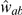) estimated from each of JTT, WAG, LG, and KHG; 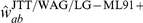 and 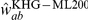 are used as 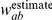 in Eq. 11. We call these models JTT/WAG/LG-ML91+ or KHG-ML200, which mean the empirical substitution matrix and the model used to estimate 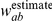, with a suffix meaning the number of ML parameters; see Table 1.

In Table 5, the ML values for these models with the various sets of parameters are listed for all empirical substitution matrices. The ML estimates in the JTT/WAG/LG-ML91+−11 and the KHG-ML200-11 models are listed in Tables 6, 7, and 8. The JTT-ML91+−0, the WAG-ML91+−0 and the LG-ML91+−0 models are the codon-based models corresponding to the JTT-F, the WAG-F and the LG-F amino-acid-based model, respectively, in which the JTT, the WAG and the LG rate matrices with an adjustment for the equilibrium frequencies of amino acids are used as a substitution rate matrix, because all 11 parameters of 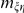, 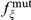, and 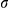 are fixed to the values of their ML estimators in the ML-91+ for JTT, WAG, and LG; 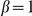 and 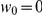 are assumed, However, a critical difference is that a genetic code cannot be taken into account in the JTT/WAG/LG-F but in the JTT/WAG/LG-ML94+−0. This difference between both models can been clearly seen in the present models applied to mtREV, because a non-universal genetic code is used in the vertebrate mitochondrial DNA. The 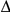
AIC is improved from 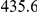 in the JTT-F to 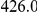 in the JTT-ML91+−0. This indicates an advantage of the present mechanistic model to the empirical amino acid substitution model.

**Table 5:** 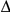
AIC values of the present models with the respective selective constraints on amino acids, 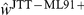, 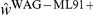, 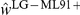, and 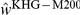, for the various 1-PAM substitution matrices.

	#parameters	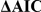 b					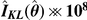 c	
Model name	#parameters (id no.^a^)	JTT	WAG	LG	cpREV	mtREV	KHG (amino acid)	KHG (codon)
JTT-ML91+−								
0	20		2657.5	20807.0	461.7	426.0		
1	21(14)		2065.1	20382.6	433.9	424.4		
4	24(1–3,14)		1773.7	16148.3	439.2	401.9		
7	27(1–3,8–10,14)		1257.8	12330.2	303.4	295.5		
11	31(1–10,14)		1152.9	12140.0	291.5	286.5	40931	
12	32(0–10,14)							473668
WAG-ML91+−								
0	20	9095.4		10537.3	316.2	535.1		
1	21(14)	8928.9		9196.3	317.1	532.8		
4	24(1–3,14)	6274.9		6354.9	281.4	414.0		
7	27(1–3,8–10,14)	3658.3		5294.9	261.6	383.6		
11	31(1–10,14)	3299.2		4813.3	259.1	365.1	12789	
12	32(0–10,14)							496804
LG-ML91+−								
0	20	13669.8	1806.0		487.1	593.4		
1	21(14)	12176.2	1188.8		421.4	558.0		
4	24(1–3,14)	6325.7	811.6		340.6	391.6		
7	27(1–3,8–10,14)	3983.0	636.0		267.0	329.8		
11	31(1–10,14)	3878.5	574.7		267.1	314.9	5732	
12	32(0–10,14)							436557
KHG-ML200-								
0	20	15063.5	953.4	12568.9	403.6	593.6		
1	21(14)	15078.6	955.4	12570.9	405.6	595.6		
4	24(1–3,14)	6398.0	540.7	5683.3	297.4	399.3		
7	27(1–3,8–10,14)	4611.5	533.4	3804.2	259.9	358.0		
11	31(1–10,14)	4429.9	518.7	3006.1	251.7	334.1		

^*a*^Parameter id numbers in the parenthesis mean ML parameters in each model and other parameters except for 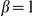 and 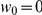 are fixed to the value of the corresponding parameter listed in the column of the ML-91 or the ML-200 in Table 3; each id number corresponds to the parameter id number listed in Table 3.

^*b*^ 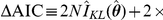 #parameters with 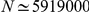 for JTT, 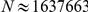 for WAG, 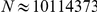 for LG, 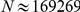 for cpREV, and 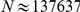 for mtREV; see text for details.

^*c*^ 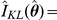 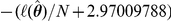 for the KHG-derived amino acid substitution probability matrix, and 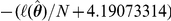 for the KHG codon substitution probability matrix; see text for details.

**Table 6:** ML estimates of the present models with the respective selective constraints for the 1-PAM amino acid substitution matrices of JTT, WAG, and LG.

	JTT			WAG			LG		
	WAG- a	LG- a	KHG- a	JTT- a	LG- a	KHG- a	JTT- a	WAG- a	KHG- a
	ML91+−11		ML200-11	ML91+−11		ML200-11	ML91+−11		ML200-11
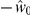	(0.0)	(0.0)	(0.0)	(0.0)	(0.0)	(0.0)	(0.0)	(0.0)	(0.0)
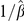	1.08	1.32	1.07	1.04	1.28	1.01	0.830	0.798	0.757
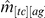	0.429	0.304	0.257	1.29	0.921	0.648	1.45	1.543	0.577
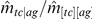	2.36	2.42	1.26	1.19	1.71	0.850	1.16	1.82	0.783
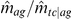	1.22	1.16	0.915	1.26	1.27	1.00	1.20	1.26	0.869
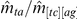	0.649	0.654	1.32	0.814	0.802	1.54	0.668	0.634	1.59
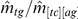	1.13	1.01	0.622	0.862	0.947	0.568	0.988	1.20	0.524
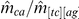	1.18	1.31	0.605	1.27	1.33	0.597	1.24	1.20	0.446
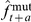	0.481	0.507	0.578	0.351	0.405	0.512	0.333	0.335	0.534
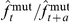	0.527	0.488	0.490	0.548	0.527	0.519	0.462	0.518	0.463
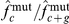	0.429	0.390	0.413	0.461	0.435	0.463	0.455	0.468	0.446
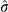	1.09	1.28	0.604	0.893	0.751	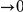	0.886	0.718	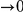
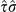	0.0263	0.0310	0.0363	0.0220	0.0230	0.0275	0.0246	0.0231	0.0444
#parameters	31	31	31	31	31	31	31	31	31
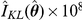 b	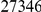	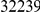	36897	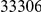	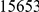	13945	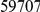	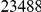	14554
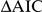 c	3299.2	3878.5	4429.9	1152.9	574.7	518.7	12140.0	4813.3	3006.1
Ratio of substitution									
rates per codon									
the total base/codon	1.35	1.32	1.19	1.51	1.45	1.19	1.47	1.49	1.12
transition/transversion	1.23	1.25	1.02	0.815	0.959	0.753	0.902	1.08	0.789
non-/synonymous^d^	1.49	1.17	0.612	2.07	1.59	0.577	1.56	1.60	0.293
For 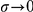									
the total base/codon	1.19	1.13	1.09	1.37	1.33	1.19	1.34	1.39	1.12
transition/transversion	1.51	1.57	1.06	0.923	1.10	0.753	1.03	1.29	0.789
non-/synonymous^d^	1.03	0.755	0.449	1.54	1.19	0.577	1.14	1.20	0.293
For 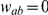 and 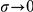									
the total base/codon	1.38	1.29	1.18	1.66	1.60	1.38	1.68	1.80	1.34
transition/transversion	1.27	1.28	0.642	0.645	0.926	0.440	0.622	0.989	0.390
non-/synonymous^d^	4.67	3.99	3.71	8.62	7.02	5.35	8.79	9.49	5.23

^*a*^In all models, equal codon usage (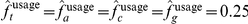) is assumed. If the value of a parameter is parenthesized, the parameter is not variable but fixed to the value specified.

^*b*^ 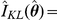 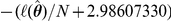 for JTT, 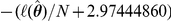 for WAG, and 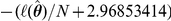 for LG.

^*c*^ 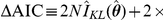 #parameters with 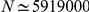 for JTT, 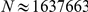 for WAG, and 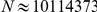 for LG; see text for details.

^*d*^Note that these ratios are not the ratios of the rates per site but per codon; see text for details.

**Table 7:** ML estimates of the present models with the respective selective constraints for the 1-PAM amino acid substitution matrices of cpREV and mtREV.

	cpREV				mtREV			
	JTT- a	WAG- a	LG- a	KHG- a	JTT- a	WAG- a	LG- a	KHG- a
	ML91+−11			ML200-11	ML91+−11			ML200-11
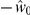	(0.0)	(0.0)	(0.0)	(0.0)	(0.0)	(0.0)	(0.0)	(0.0)
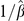	0.940	0.977	1.18	1.02	0.690	0.845	0.977	0.752
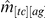	0.865	0.917	0.611	0.521	0.564	0.524	0.321	0.228
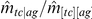	1.50	2.23	2.353	1.14	2.01	3.43	3.82	1.64
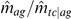	1.28	1.30	1.24	0.973	1.06	1.13	1.08	0.752
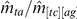	0.746	0.705	0.733	1.61	0.681	0.595	0.638	2.00
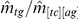	1.17	1.37	1.25	0.747	0.792	0.893	0.839	0.411
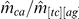	1.23	1.17	1.26	0.566	1.65	1.67	1.76	0.623
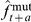	0.283	0.306	0.328	0.442	0.262	0.270	0.287	0.426
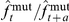	0.611	0.654	0.609	0.597	0.601	0.652	0.598	0.631
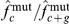	0.425	0.446	0.393	0.425	0.349	0.304	0.260	0.332
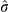	1.93	1.43	1.75	0.158	3.48	2.18	3.37	2.89
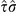	0.0325	0.0285	0.0339	0.0288	0.0603	0.0445	0.0653	0.0923
#parameters	31	31	31	31	31	31	31	31
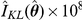 b	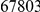	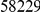	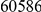	56032	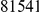	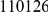	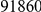	98837
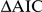 c	291.5	259.1	267.1	251.7	286.5	365.1	314.9	334.1
Ratio of substitution								
rates per codon								
the total base/codon	1.45	1.46	1.41	1.20	1.36	1.37	1.33	1.23
transition/transversion	1.05	1.20	1.25	1.05	1.44	1.65	1.74	1.45
non-/synonymous^d^	1.74	1.80	1.38	0.631	0.908	1.04	0.772	0.403
For 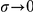								
the total base/codon	1.21	1.26	1.20	1.16	1.11	1.15	1.09	1.05
transition/transversion	1.42	1.66	1.77	1.07	2.52	2.73	3.31	1.96
non-/synonymous^d^	1.03	1.10	0.794	0.573	0.387	0.515	0.312	0.163
For 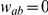 and 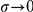								
the total base/codon	1.45	1.55	1.44	1.33	1.31	1.37	1.26	1.16
transition/transversion	0.797	1.20	1.25	0.569	1.06	1.78	1.98	0.883
non-/synonymous^d^	6.06	6.33	5.14	4.97	3.40	3.09	2.58	3.02

^*a*^In all models, equal codon usage (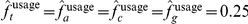) is assumed. If the value of a parameter is parenthesized, the parameter is not variable but fixed to the value specified.

^*b*^ 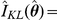 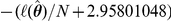 for cpREV, and 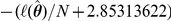 for mtREV; see text for details.

^*c*^ 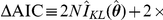 #parameters with 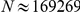 for cpREV, and 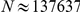 for mtREV; see text for details.

^*d*^Note that these ratios are not the ratios of the rates per site but per codon; see text for details.

**Table 8:** ML estimates of the present models with the respective selective constraints for the 1-PAM KHG-derived amino acid and KHG codon substitution matrices.

	KHG (amino acid)			KHG (codon)		
	JTT- a	WAG- a	LG- a	JTT- a	WAG- a	LG- a
	ML91+−11			ML91+−12		
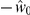	(0.0)	(0.0)	(0.0)	1.29	1.50	1.11
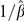	0.952	0.912	1.22	1.72	2.02	1.91
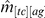	1.545	1.68	1.33	1.23	1.21	1.15
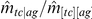	1.19	1.73	1.69	0.992	1.07	1.09
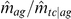	1.24	1.28	1.22	1.09	1.12	1.10
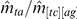	0.689	0.682	0.748	1.26	1.25	1.25
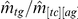	0.855	1.07	0.943	0.646	0.662	0.671
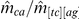	1.32	1.26	1.31	0.815	0.806	0.813
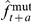	0.317	0.334	0.377	0.480	0.484	0.488
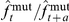	0.533	0.579	0.512	0.499	0.499	0.493
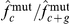	0.460	0.480	0.441	0.464	0.459	0.459
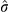	2.64	2.25	1.30	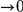	0.0496	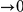
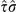	0.0308	0.0286	0.0247	0.0240	0.0247	0.0240
#parameters	31	31	31	32	32	32
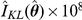 b	40931	12789	5732	473668	496804	436557
Ratio of substitution						
rates per codon						
the total base/codon	1.64	1.66	1.59	1.29	1.29	1.29
transition/transversion	0.772	0.859	0.891	0.759	0.765	0.767
non-/synonymous*^c^*	2.56	2.61	2.03	0.728	0.727	0.724
For 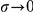						
the total base/codon	1.39	1.45	1.43	1.29	1.28	1.29
transition/transversion	0.977	1.15	1.08	0.759	0.770	0.767
non-/synonymous^*c*^	1.48	1.54	1.36	0.728	0.704	0.724
For 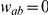 and 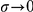						
the total base/codon	1.71	1.83	1.75	1.65	1.65	1.64
transition/transversion	0.637	0.926	0.892	0.51	0.552	0.561
non-/synonymous*^c^*	9.41	10.3	8.86	8.16	8.07	7.77

^*a*^In all models, codon frequencies are taken to be equal to the observed ones. If the value of a parameter is parenthesized, the parameter is not variable but fixed to the value specified.

^*b*^ 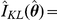 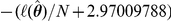 for the KHG-derived amino acid substitution probability matrix, and 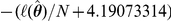 for the KHG codon substitution probability matrix; see text for details.

^*d*^Note that these ratios are not the ratios of the rates per site but per codon; see text for details.

The AIC values of the JTT/WAG/LG-ML91+−0 are better for all the four matrices (JTT, WAG, cpREV, and mtREV) than those of the physico-chemical method EI-11; compare Tables 2 and 5. The AIC values of the KHG-200-0 are better for all except for JTT than those of the EI-11. The AIC values of all the models are drastically improved for all the matrices by optimizing the 11 parameters; see Table 5. It is noteworthy that all the models of the JTT-ML91+−11, the LG-ML91+−11, and the KHG-ML200-11 yield a better AIC value for WAG than the ML-87 model does, rejecting the null hypothesis of no multiple nucleotide change again; see Tables 3 and 5. Thus, the ML estimates 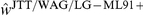 and 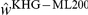 sufficiently represent selective constraints on amino acid substitutions.

In addition, Table 5 indicates which parameters are the most effective for improving AIC. As well as the EI models, the JTT/WAG/LG-ML91+−7, in which the parameters 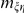 are fixed to the ML estimates for JTT/WAG/LG with a certain ratio of transition to transversion exchangeability, can improve the AIC up to the similar degree to the AIC values of the JTT/WAG/LG-ML91+−11, respectively. In other words, the parameters 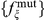 are very effective to improve the AIC in comparison with the parameters 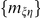.

**Figure 4:**
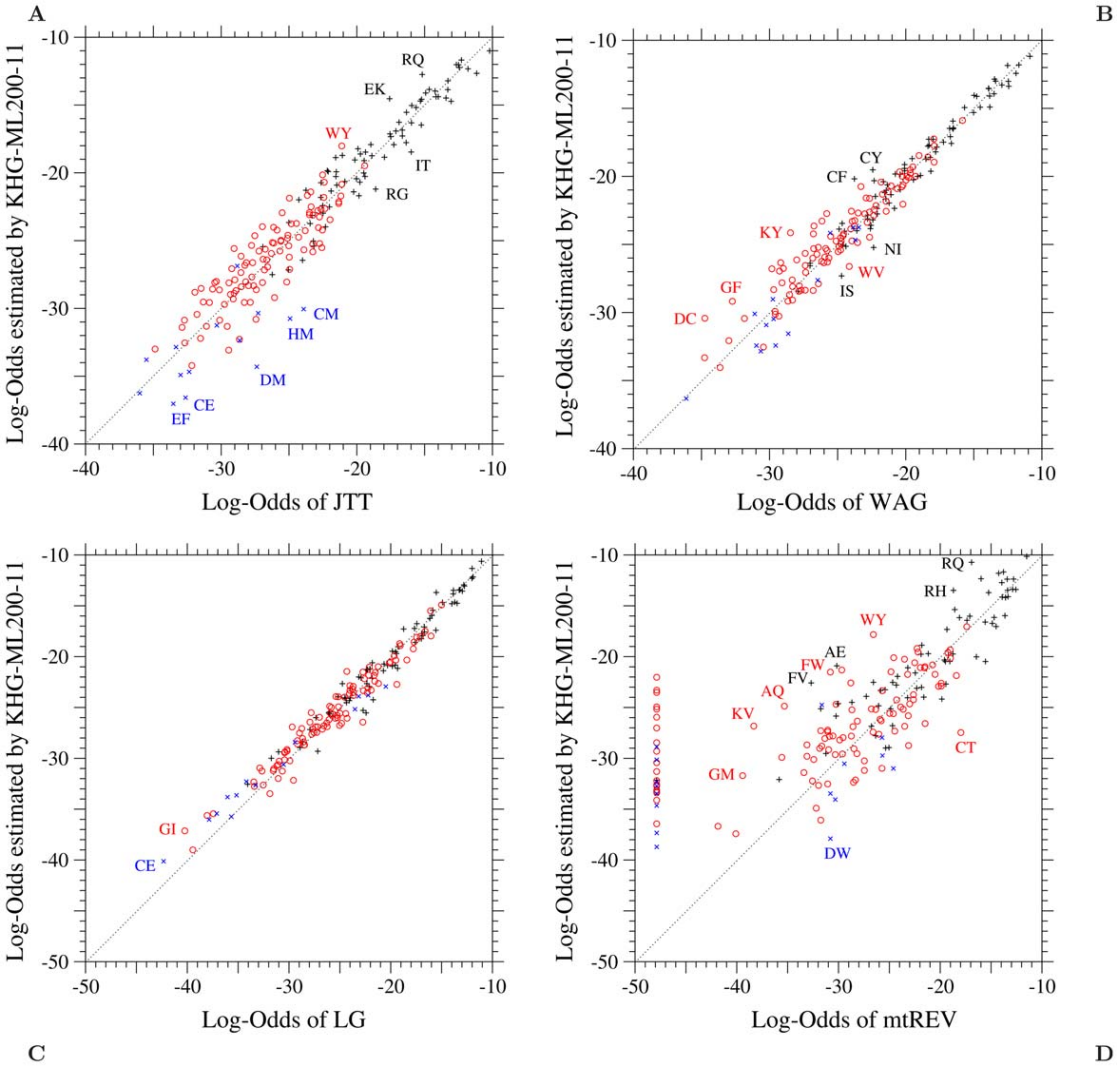
The KHG-ML200-11 model fitted to each of JTT, WAG, LG, and mtREV. Each element log-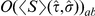 of the log-odds matrices of the KHG-ML200-11 model fitted to the 1-PAM matrices of (A) JTT, (B) WAG, (C) LG, and (D) mtREV is plotted against the log-odds log-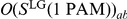 calculated from the corresponding empirical substitution matrices. Plus, circle, and cross marks show the log-odds values for one-, two-, and three-step amino acid pairs, respectively. The dotted line in each figure shows the line of equal values between the ordinate and the abscissa. The log-odds elements of mtREV whose values are smaller than about 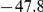 are all assumed to be 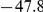; see the original paper [6].

The log-odds values of amino acid pairs estimated by the KHG-ML200-11 are plotted against their empirical values for the 1-PAM amino acid substitution matrices of JTT, WAG, LG, and mtREV in Fig. 4. Similar plots are shown in Figs. S5 – S10. The comparisons of Fig. 1 and Fig. S1 for the ML-87 model with Fig. 4 and Fig. S5 clearly indicate the good qualities of the ML estimators 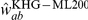 and 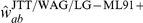. Relatively large disagreements between empirical and estimated log-odds exist for cpREV and mtREV in comparison with those for JTT, WAG, LG, and the KHG-derived amino acid substitution matrix (KHGaa); see Fig. 4 and Figs. S5 – S7. It is unknown whether the disagreements shown in these figures represent meaningful features in the amino acid substitutions in the chloroplast DNA and the mitochondrial DNA or result from the relatively small size of sequence data used for cpREV and mtREV. However, the large disagreements in the region of low log-odds values may be artifacts, because cpREV and mtREV tend to include relatively large errors in this region, especially for mtREV; the log-odds values for mtREV whose values are smaller than about 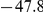 are all assumed to be 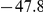; see the original paper [6].

The ML estimates of 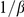 listed in Tables 6, 7, and 8 indicate that the strength of selective constraints on amino acids is strong in the order of LG, WAG, and JTT. The strength of selective constraints is also shown by the change of the ratio of nonsynonymous to synonymous rate per codon between the two cases without and with selective constraints, i.e., the cases of 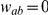 and 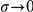, and 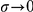. As already noted, the ratio of these values between the two cases represents the strength of selective constraints. In the KHG-ML200-11, these ratios are equal to 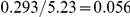, 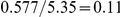, and 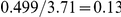 for LG, WAG, and JTT, respectively, meaning that the selective constraints of LG are strongest; it should be noted that this order agrees with the increasing order of 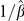.

Tables 6 and 7 indicate that the selective constraints 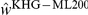 estimated from the KHG codon substitution matrix tend to estimate the contribution of multiple nucleotide changes (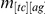) to be smaller, the ratio of transition to transversion exchangeability (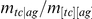) to be smaller, 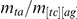 to be larger, and variations in substitution rates (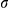) to be less than the 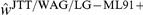 from the amino acid substitution matrices. Table 8 shows that the same characteristic differences will be observed if the JTT/WAG/LG-ML91+−11 models are fitted to the codon substitution matrix of KHG instead of its derived amino acid substitution matrix. Tables 6, 7, and 8 also show that the ratio of transition to transversion exchangeability (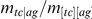) tends to be estimated to be smaller in the order of the LG-ML91+, the WAG-ML91+, the JTT-ML91+, and the KHG-ML200. The 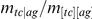 is estimated by the ML-91 or the ML-200 model to be smaller in the order of LG, WAG, JTT, and KHG; see Table 3. The present ML estimates 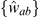 for selective constraints on amino acids seem to reflect the characteristics of respective substitution matrices to which the models are fitted. It remains to be analyzed which estimation is better among the JTT/WAG/LG-ML91+ and the KHG-ML200 and how better it is. Irrespective of which estimation of the selection constraints is better, the ML estimates 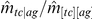 indicate that the transition to transversion bias is not so strong as previously estimated.

One of the interesting facts is that the ratio of the total transition to the total transversion rate per codon will be estimated to be much larger if multiple nucleotide changes are neglected; 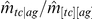 (and the ratio of the total transition to the total transversion rate for 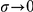) are estimated for the mtREV to be 2.15 (3.32) in the JTT-ML91+−10 but 2.01 (2.52) in the JTT-ML91+−11, 4.27 (4.13) in the WAG-ML91+−10 but 3.43 (2.73) in the WAG-ML91+−11, 4.57 (4.74) in the LG-ML91+−10 but 3.82 (3.31) in the LG-ML91+−11, and 1.81 (2.58) in the KHG-ML200-10 but 1.64 (1.96) in the KHG-ML200-11. The same tendency is observed for JTT, WAG, cpREV, and mtREV irrespective of the matrices, and for the EI, the Miyata, and the Grantham models irrespective of the models.

In the case of mtREV, not only the transition-transversion exchangeability bias (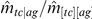) but also the ratio of the total transition to the total transversion rate per codon is larger in the JTT/WAG/LG-ML91+−11 than in the JTT/WAG/LG-ML91+−0, and in the KHG-ML200-11 than in the KHG-ML200-0. Also, the JTT/WAG/LG-ML91+−11 and the KHG-ML200-11 models estimate 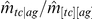 and the ratio of the total transition to the total transversion rate to be larger for mtREV than for JTT, WAG, and cpREV. These results are consistent with a well-known fact that transition to transversion bias is larger in mitochondrial DNA than in nuclear DNA.

## Discussion

Halpern and Bruno [40] considered a codon-substitution model in which site-specific selection is taken into account in terms of residue frequencies. If site-specific codon frequencies are explicitly taken into account in the present model, the substitution rate 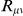 will be regarded as the average of the site-specific rate 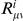 over sites 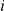. According to Eq. 7, the site-specific rate is defined as the product of site-independent mutation rate 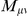 and site-dependent fixation probability, 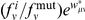.
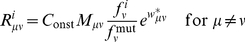
(29)


Here the site-dependency of the fixation probability is taken into account only in terms of codon frequencies. Then, the average of the site-specific rate over sites is calculated as follows. 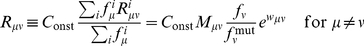
(30)

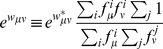
(31)


where 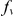 is the average of 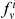 over sites. Thus, the 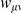 defined here includes the effects of site-specific selection in terms of codon frequencies.

In the model of Halpern and Bruno [40], the term of 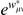 was not distinguished from and merged with the mutation rate 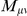; that is, 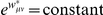 for 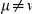 was assumed, Yang and Nielsen [22] considered mutation-selection models of codon substitutions and estimated selective strengths on codon usage. In their models, selection pressures that deviate codon frequencies from the equilibrium codon frequencies at the mutational level were explicitly taken into account, and selective constraints on amino acids are assumed to be constant over amino acid pairs; that is, 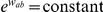 for 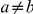 was assumed. However, the site-specific selection was not considered; that is, 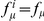. In other words, unlike the present model, selection was taken into account principally in terms of codon or residue frequencies in both the models. Also. multiple nucleotide changes were not taken into account. Halpern and Bruno [40] developed their model for distance calculation. As pointed out by Yang and Nielsen [22], taking account of site-specific codon frequencies is not practical for real data analysis due to the use of too many parameters. Instead, the use of 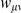 is more practical. The present results show that the ML values of the JTT/WAG/cpREV/mtREV amino acid substitution matrices are too small in the No-Constraints models in which 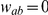 is assumed, and they can be improved by taking account of the term of the selective constraints 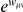. Also, it is indicated that selective constraints on amino acids strongly depend on the type of amino acid.

In some previous models [7], [17], [18], amino acid substitutions were assumed to proceed in a stepwise manner by successive single nucleotide changes in a codon. The empirical amino acid substitution matrices of JTT, WAG, LG, cpREV, and mtREV, and the codon substitution matrix KHG all include many substitutions between amino acid or codon pairs requiring multiple nucleotide changes. Significance of multiple nucleotide substitutions was pointed out [7], [14], [20], [27], [29]. There are two possible mechanisms to yield substitutions between such multi-step amino acid pairs even for a short time interval. One is variations in substitution rates or time intervals. Another is multiple nucleotide changes in a codon. Here, the assumption of multiple nucleotide changes has been directly introduced into a codon-based substitution model together with the use of a 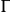 distribution for variations in substitution rates and time intervals, and the effectiveness of the assumption has been examined.

In the models using any physico-chemical evaluation of selective constraints, the significance of multiple nucleotide changes has been indicated; see Tables 2 and 3. The ML-87 models fitted to JTT and WAG, in which the selective constraints 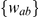 for all single-step amino acid pairs are optimized by maximizing the likelihood with the assumptions of no multiple nucleotide change for codon substitutions and of variations in substitution rates, reveal that large discrepancies between the observed and the estimated log-odds values remain for multi-step amino acid pairs; see Fig. 1. When multiple nucleotide changes are taken into account in the model ML-91, these discrepancies disappear and the AIC values significantly decrease, indicating the significance of multiple nucleotide changes in codon substitutions; see Fig. 1, Fig. S1, and Table 3.

Evidence for multiple nucleotide changes was found by Averof et al. [27], and the frequency of multiple nucleotide changes was evaluated [20]. On the other hand, a possibility for successive single compensatory substitutions was pointed out by Bazykin et al. [29]. As pointed out by Kosiol et al. [14], the high exchangeabilities of the double nucleotide changes, Rcgt 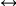 Ragg and Rcgt 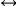 Raga, in KHG may result from successive single compensatory substitutions. On the other hand, a selection on synonymous substitutions is necessary for compensatory substitutions to cause the higher exchangeability of Rcga 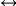 Ragg than estimated, because the most probable paths of single nucleotide changes between Rcga and Ragg are Rcga 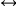 Raga 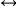 Ragg and Rcga 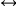 Rcgg 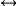 Ragg both of which do not accompany any amino acid change; see Fig. 2. Whatever causes multiple nucleotide changes, the present scheme for codon substitutions could be applied to phylogenetic analyses of protein-coding sequences, because the underlying time scale in the present substitution model is much longer than that of positive selection for successive single compensatory substitutions.

The models JTT/WAG/LG-ML91+−0 and KHG-ML200-0, in which parameters are taken to be equal to the ML estimates for JTT/WAG/LG in the ML-91+ model and the ML estimates for KHG in the ML-200 model, are codon-based models corresponding to the JTT/WAG/LG/KHG-F model, respectively. The model ML-91+ can almost perfectly reproduce JTT, WAG, and LG. The model ML-200 for the KHG codon substitution matrix can well reproduce the codon substitution probabilities for the codon pairs for which any nucleotide change is accompanied by an amino acid change, although the exchangeabilities of the other codon pairs are over-estimated for KHG. This means that the JTT/WAG/LG-ML91+−0 and the KHG-ML200-0 models can be used as a simple substitution model without any loss of information instead of the empirical substitution matrices of the JTT/WAG/LG/KHG in maximum likelihood and Bayesian inferences of phylogenetic trees of amino acid and codon sequences, respectively. Although the empirical substitution matrices represent the average tendencies of substitutions over proteins and species and may lack gene-level resolution [15], [16], the present mechanistic codon model has adjustable parameters for nucleotide mutation and for the strength of selective constraints, which can be tailored to specific genes. It is possible to optimize the selective constraints 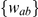 for each gene. However, such a method [12], [15], [16] is far more computer-intensive than the present method. The present methods, JTT/WAG/LG-ML91+−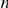 using 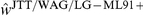 and the KHG-ML200-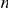 with the 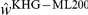, provide alternative models for amino acid/codon substitutions with a small number of ML parameters in the probabilistic inference of phylogenetic trees. The number of ML parameters specific to the present model is at most 6 exchangeabilities and 3 equilibrium frequencies for nucleotide mutations, and 2 parameters for selective constraints. Thus, the present model requires the same order of cpu time as the nucleotide substitution model (GTR) does. In other codon models [21], [23], exchangeabilities between amino acids are taken to be equal to their values in empirical amino acid substitution matrices. However, in the present codon model, amino acid and codon exchangeabilities vary according to nucleotide mutation rates and the strength of selective constraints.

The parameters 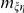, 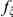, and 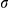 are differently estimated by the KHG-ML200-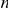 and the JTT/WAG/LG-ML91+−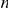 using different 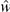; see Tables 6, 7, and 8. The 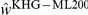 yields a smaller rate of multiple nucleotide changes, a smaller 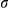, a smaller ratio of transition to transversion exchangeability, and a smaller ratio of nonsynonymous to synonymous rate per codon than the 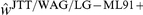 does. Whichever estimation is better, the present ML estimators 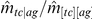 for transition-transversion bias strongly indicate that the transition-transversion bias is not so large as previously estimated. An excess of transitional over transversional substitutions was shown in the DNA sequences of metazoa, and has been assumed to be universal. However, Keller et al. [41] found a counter example to the transition-transversion bias from grasshopper pseudogenes. The present ML estimate of the ratio of transition to transversion exchangeability for the KHG codon substitution matrix is rather less than 1.0, i.e., 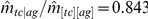 in the ML-200 model, which corresponds to the overall rate bias of transitions over transversions, 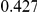. Even for the amino acid substitution matrices JTT, WAG, and LG, the ML-91 model estimates 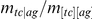 to be less than 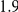, making the overall rate bias of transitions over transversions less than 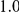; see Table 3. It should be noted that the ratio of transition to transversion exchangeability tends to be overestimated if no multiple nucleotide change is allowed; see Tables S2 and S3. Thus, the present results indicate that transition-transversion bias is not a solid assumption. On the other hand, the present results indicate that transition-transversion bias is stronger in mitochondrial DNA than in nuclear DNA in accordance with previous understanding; see Tables 6 and 7.

The ML estimates 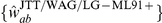 and 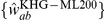 significantly correlate with each other and also with the mean energy increments due to an amino acid replacement. However, the JTT/WAG/LG-ML91+−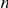 and KHG-ML200-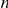 models fit substitution data significantly better than the EI-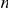 model; see Tables 2 and 5. This fact indicates that the differences between the physico-chemical estimates and the ML estimates 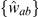 for selective pressure at the amino acid level reflect the actual tendency of selective constraints for respective types of amino acid pairs in protein evolution. Eq. 31 indicates that the 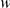 is modulated by site-specific codon frequencies and differentiated from the site-independent constraints, 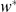, which may be more similar to the physico-chemical estimates than the 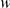. The selective constraints estimated here may be used as a base line to detect evidence of positive selection. Models [20], [22] in which the dependences of selective constraints on amino acid pairs are not taken into account may be improved by introducing them. On the other hand, it still remains to be examined whether or not the JTT/WAG/LG-ML91+−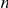 and the KHG-ML200-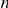 perform comparably with cpREV for the maximum likelihood inferences of phylogenetic trees of chloroplast proteins and with mtREV for those of mitochondrial proteins. Also, it should be examined which performs better.

A preliminary calculation has been pursued to examine the performance of the present substitution models in the ML inference of a phylogenetic tree. Log-likelihoods of the present models and the codon models corresponding to the mtREV-F, the JTT-F, the WAG-F, and the LG-F are calculated and listed in Table 9 for a phylogenetic tree [6] of the concatenated sequences of 12 protein-coding sequences encoded on the same strand of mitochondrial DNA from 20 vertebrate species with 2 races from human. The phylogenetic tree and the proteins used are those which Adachi and Hasegawa [6] used to estimate mtREV; the Japanese mtDNA was not used because it couldn't be found in the GenBank database. The coding sequences of each protein were aligned with codon score matrices by the ClustalW2 [42], and then concatenated. Their likelihoods on the phylogenetic tree were calculated by the Phyml [43]. Both the programs have been modified for the analysis of coding sequences. Log-odds calculated by the KHG-ML200-11 fitted to mtREV were used as the codon score matrices. Positions with gaps are included for the calculation of the likelihoods. The codon substitution matrices corresponding to mtREV, JTT, WAG, LG, and the KHG-derived amino acid substitution matrix (KHGaa) are calculated in such a way that codon exchangeabilities for nonsynonymous codon pairs are taken to be equal to 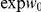 multiplied by the exchangeability of the corresponding amino acid pair and those for synonymous codon pairs are assumed to be all equal to the mean amino acid exchangeability. In all models, the parameter 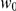 in Eq. 11 was optimized even for the No-Constraints models, and codon frequencies were taken to be equal to those in coding sequences. The substitution matrices, JTT, WAG, LG, and KHG were estimated from nuclear DNA, which use a different genetic code from vertebrate mtDNA. On the other hand, mtREV was estimated by a maximum likelihood method from the almost same set of the protein sequences encoded in mtDNA. Thus, it is expected that the log-likelihood values of the mtDNA phylogenetic tree for the models, KHGaa-1-F, LG-1-F, WAG-1F, and JTT-1-F are worse than that for the mtREV-1-F. An important thing is that the codon models with the selective constraints estimated from nuclear DNA or by the physico-chemical method yield a much smaller value of AIC than the mtREV-1-F. One of the effective parameters is 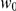 that directly controls the ratio of nonsynonymous to synonymous substitution rate. It also improves the likelihood to explicitly take account of rate variations over sites. The discrete approximation [44] of the 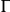 distribution with 4 categories was used to represent rate variations over sites in the models named with the suffix "dG4"; the shape parameter 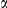 is a ML parameter. An interesting and reasonable fact is that averaging substitution matrices over rate becomes unnecessary, i.e., 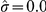, in the case that rate variations over sites are explicitly taken into account; in the Yang's model [26], [44], the likelihood of a phylogenetic tree of each site is averaged over rate. Also, all the present codon-based models estimate 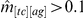, which indicates the significance of multiple nucleotide changes. The present results strongly indicate that the tendencies of nucleotide mutations and codon usage are characteristic of a genetic system specific to each species and oranelle, but the amino acid dependences of selective constraints are more specifc to each type of amino acid than each species, organelle, and protein family. Full evaluation will be provided in a succeeding paper.

**Table 9:** Log-likelihoods of a phylogenetic tree [6] of the concatenated sequences of 12 protein-coding sequences encoded on the same strand of mitochondrial DNA from 20 vertebrate species with 2 races from human.

Codon Substitution	#p^b^	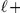	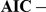	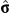	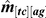	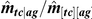
Model^a^		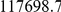	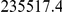			
KHGaa-1-F*^cd^*	60	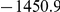	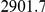			
LG-1-F^c^	60	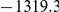	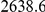			
WAG-1-F^c^	60	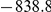	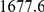			
JTT-1-F^c^	60	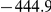	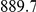			
mtREV-1-F^c^	60	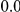	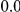			
No-Constraints-1-F^e^	60	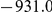	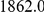	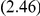	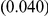	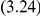
WAG-ML91+−1-F^e^	60	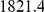	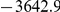	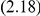	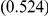	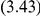
JTT-ML91+−1-F^e^	60	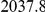	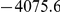	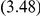	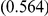	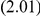
LG-ML91+−1-F^e^	60	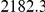	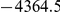	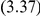	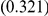	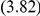
EI-1-F^e^	60	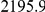	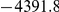	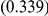	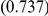	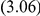
KHG-ML200-1-F^e^	60	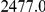	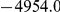	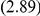	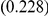	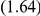
No-Constraints-11-F	70	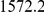	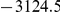	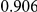	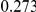	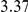
EI-12-F	71	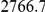	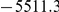	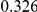	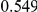	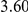
WAG-ML91+−12-F	71	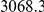	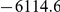	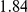	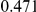	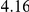
JTT-ML91+−12-F	71	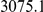	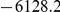	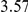	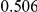	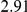
KHG-ML200-12-F	71	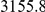	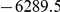	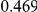	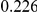	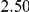
LG-ML91+−12-F	71	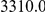	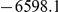	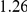	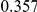	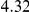
No-Constraints-11-F-dG4	71	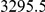	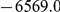	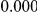	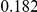	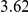
EI-12-F-dG4	72	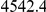	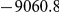	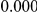	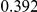	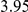
JTT-ML91+−12-F-dG4	72	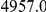	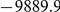	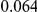	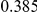	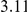
KHG-ML200-12-F-dG4	72	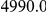	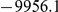	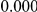	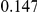	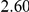
WAG-ML91+−12-F-dG4	72	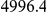	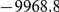	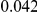	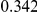	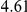
LG-ML91+−12-F-dG4	72	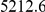	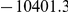	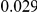	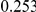	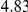

a aIn all models named with a suffix "F", codon frequencies are taken to be equal to those in coding sequences. A suffix "dG4" means the discrete approximation of the 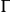 distribution with 4 categories [44] for rate variation. The parameter 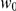 in Eq. 11 is optimized in all models.

bThe number of parameters; the value for the mtREV-1-F is not quite correct, because mtREV was estimated from the almost same set of protein sequences [6].

cThe exchangeabilties of nonsynonymous and synonymous codon pairs are equal to 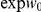 multiplied by those of the corresponding amino acid pairs and all equal to the mean amino acid exchangeability in the empirical amino acid substitution matrix specified, respectively.

dKHGaa means the amino acid substitution matrix derived from KHG.

eAll parameters except 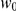 and codon frequencies are fixed to those ML estimates of each model fitted to mtREV.

One may question whether the whole evolutionary process of protein-coding sequences can be approximated by a reversible Markov process or not. Kinjo and Nishikawa [45] reported that the log-odds matrices constructed for 18 different levels of sequence identities from structure-based protein alignments have a characteristic dependence on time in the principal components of their eigenspectra. Although they did not explicitly mention, this type of temporal process peculiar to the log-odd matrix in protein evolution is fully encoded in the transition matrices of JTT, WAG, LG, and KHG. In Fig. S11, it is shown that this characteristic dependence of log-odds on time can be reproduced by the transition matrix based on the present reversible Markov model fitted to JTT; see Text S1 for details. This fact supports the appropriateness of the present Markov model for codon substitutions. The present codon-based model can be used to generate log-odds for codon substitutions as well as amino acid substitutions. Such a log-odds matrix of codon substitutions would be useful to allow us to align nucleotide sequences at the codon level rather than the amino acid level, increasing the quality of sequence alignments.

As a result, the present model would enable us to obtain more biologically meaningful information at both nucleotide and amino acid levels from codon sequences and even from protein sequences, because this is a codon-based model.

## Supporting Information

Text S1 **Supporting information consisting of the following sections.** 1. A method for the physico-chemical evaluation of selective constraints on amino acid replacement. 2. Models with no amino acid dependences of selective constraints. 3. A physico-chemical evaluation of selective constraints on amino acids. 4. Other physico-chemical evaluations of selective constraints on amino acids. 5. Evolutionary process of amino acid substitutions in terms of log-odds.
(PDF)

Data S1 **A computer-readable dataset of the ML estimates of parameters in the ML-200 for KHG, and the ML-91 and the ML-91+ for LG, WAG, and JTT as well as the EI.**
(TXT)

Figure S1
**The ML-87 and the ML-91 models fitted to WAG.** Each element log-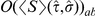 of the log-odds matrices of (A) the ML-87 and (B) the ML-91 models fitted to the 1-PAM WAG matrix is plotted against the log-odds log-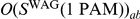 calculated from WAG. Plus, circle, and cross marks show the log-odds values for one-, two-, and three-step amino acid pairs, respectively. The dotted line in each figure shows the line of equal values between the ordinate and the abscissa.(PDF)

Figure S2
**Comparison between various estimates of selective constraint for each amino acid pair** The ML estimates of selective constraint on substitutions of each amino acid pair are compared between the models fitted to various empirical substitution matrices. The estimates 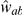 for multi-step amino acid pairs that belong to the least exchangeable class at least in one of the models are not shown. Plus, circle, and cross marks show the values for one-, two-, and three-step amino acid pairs, respectively.(PDF)

Figure S3
**Selective constraint for each amino acid pair estimated from WAG and from LG.** The ML estimate, 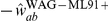 in (A) and 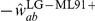 in (B), of selective constraint on substitutions of each amino acid pair in the ML-91+ models fitted to the 1-PAM matrices of WAG and LG is plotted against the mean energy increment due to an amino acid substitution, (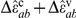) defined by Eqs. S1-4, S1-5, and S1-6 in Text S1. The estimates 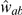 for the least exchangeable class of multi-step amino acid pairs are not shown. Plus, circle, and cross marks show the values for one-, two-, and three-step amino acid pairs, respectively.(PDF)

Figure S4 **Comparison of the ML estimates of selective constraint for each amino acid pair between the ML-87 and the ML-91 models.** The ML estimate of selective constraint for each single step amino acid pair in the ML-87 model fitted to (A) the 1-PAM JTT matrix or (B) the 1-PAM WAG matrix is plotted against that in the ML-91 model.
(PDF)

Figure S5
**Models fitted to each of JTT, WAG, and LG.** Each element log-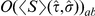 of the log-odds matrix of the model fitted to each empirical substitution matrix is plotted against the log-odds log-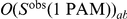 calculated from the corresponding empirical substitution matrix. Plus, circle, and cross marks show the log-odds values for one-, two-, and three-step amino acid pairs, respectively. The dotted line in each figure shows the line of equal values between the ordinate and the abscissa.(PDF)

Figure S6
**Models fitted to each of cpREV and mtREV.** Each element log-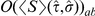 of the log-odds matrix of the model fitted to each empirical substitution matrix is plotted against the log-odds log-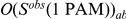 calculated from the corresponding empirical substitution matrix. Plus, circle, and cross marks show the log-odds values for one-, two-, and three-step amino acid pairs, respectively. The dotted line in each figure shows the line of equal values between the ordinate and the abscissa.(PDF)

Figure S7
**Models fitted to the KHG-derived amino acid substitution matrix.** Each element log-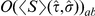 of the log-odds matrix of the model fitted to the 1-PAM KHG-derived amino acid substitution matrix (KHGaa) is plotted against the log-odds log-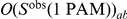 calculated from KHGaa. Plus, circle, and cross marks show the log-odds values for one-, two-, and three-step amino acid pairs, respectively. The dotted line in each figure shows the line of equal values between the ordinate and the abscissa.(PDF)

Figure S8
**The JTT-ML91+−12 model fitted to the 1-PAM KHG codon substitution matrix.** Each element log-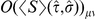 of the log-odds matrix corresponding to (A) single, (B) double, and (C) triple nucleotide changes in the JTT-ML91+−12 model fitted to the 1-PAM KHG codon substitution matrix is plotted against the log-odds log-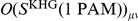 calculated from KHG. Upper triangle, plus, circle, and cross marks show the log-odds values for synonymous pairs and one-, two-, and three-step amino acid pairs, respectively. The dotted line in each figure shows the line of equal values between the ordinate and the abscissa.(PDF)

Figure S9
**The WAG-ML91+−12 model fitted to the 1-PAM KHG codon substitution matrix.** Each element log-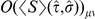 of the log-odds matrix corresponding to (A) single, (B) double, and (C) triple nucleotide changes in the WAG-ML91+−12 model fitted to the 1-PAM KHG codon substitution matrix is plotted against the log-odds log-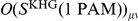 calculated from KHG. Upper triangle, plus, circle, and cross marks show the log-odds values for synonymous pairs and one-, two-, and three-step amino acid pairs, respectively. The dotted line in each figure shows the line of equal values between the ordinate and the abscissa.(PDF)

Figure S10
**The LG-ML91+−12 model fitted to the 1-PAM KHG codon substitution matrix.** Each element log-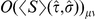 of the log-odds matrix corresponding to (A) single, (B) double, and (C) triple nucleotide changes in the LG-ML91+−12 model fitted to the 1-PAM KHG codon substitution matrix is plotted against the log-odds log-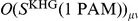 calculated from KHG. Upper triangle, plus, circle, and cross marks show the log-odds values for synonymous pairs and one-, two-, and three-step amino acid pairs, respectively. The dotted line in each figure shows the line of equal values between the ordinate and the abscissa.(PDF)

Figure S11Temporal changes of the eigenvalues and the eigenvectors of the log-odds matrix log-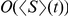 calculated by the ML-91+ model fitted to JTT as a function of sequence identity. In (A), the solid, the broken, and the dotted lines show the temporal changes of the first (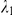), the second (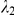), and the third (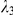) principal eigenvalues, respectively. The inner products of the eigenvectors with the eigenvectors of the JTT 20-PAM log-odds matrix, 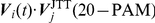, are shown in (B) for the first principal eigenvector (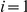), in (C) for the second principal eigenvector (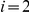), and in (D) for the third principal eigenvector (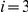), by solid lines for 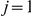, by broken lines for 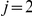, and by dotted lines for 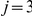.(PDF)

Table S1 **ML estimates of the present models without selective constraints on amino acids for the 1-PAM substitution matrices of JTT, WAG, cpREV, and mtREV.**
(PDF)

Table S2 **ML estimates of the present models with the selective constraints based on mean energy increments due to an amino acid substitution (EI) for the 1-PAM substitution matrices of JTT, WAG, cpREV, and mtREV.**
(PDF)

Table S3 **ML estimates of the present models with the selective constraints based on the Grantham's and the Miyata's amino acid distances for the 1-PAM substitution matrices of JTT and WAG.**
(PDF)
