# Supplementary material for: Selective Constraints on Amino Acids Estimated by a Mechanistic Codon Substitution Model with Multiple Nucleotide Changes
Source: PLoS One. 2011 Mar 18;6(3):e17244. doi: 10.1371/journal.pone.0017244 (PMC3060808; doi:10.1371/journal.pone.0017244)
Supplement: Text S1 — Supporting information consisting of the following sections. 1. A method for the physico-chemical evaluation of selective constraints on amino acid replacement. 2. Models with no amino acid dependences of selective constraints. 3. A physico-chemical evaluation of selective constraints on amino acids. 4. Other physico-chemical evaluations of selective constraints on amino acids. 5. Evolutionary process of amino acid substitutions in terms of log-odds. (PDF) [file pone.0017244.s001.pdf]

Supporting Information
for
Selective Constraints on Amino Acids
Estimated by a Mechanistic Codon Substitution Model
with Multiple Nucleotide Changes

Sanzo Miyazawa

Graduate School of Engineering
Gunma University
Kiryu, Gunma 376-8515, Japan
Phone: +81-277-30-1940
E-Mail: miyazawa@smlab.sci.gunma-u.ac.jp
sanzo.miyazawa@gmail.com

(February 9, 2011)

Supplementary Methods

A method for the physico-chemical evaluation of selective constraints on amino acid replacements

Physico-chemical evaluations of $\{w_{ab}\}$ are not meaningless, even though selective constraints $\{w_{ab}\}$ in Eq. 9 in the text can be optimized for observed data. Their performance in reproducing observed substitution data indicates how extensively selective constraints on amino acid substitutions can be explained by physico-chemical requirements on amino acid substitutions to preserve protein structures and functions. In this section, a new physico-chemical method for the evaluation of the selective constraints is introduced.

The rate of acceptance in amino acid replacements is assumed here to be proportional to the mean relative stability of the native conformation $\mathcal{C}$ of the mutant type of sequence $\mathcal{S}'$ to that of the wild type of sequence $\mathcal{S}$. The probability $P(\mathcal{C}|\mathcal{S})$ of a conformation $\mathcal{C}$ that a sequence $\mathcal{S}$ takes is equal to the Boltzmann factor of $\mathcal{C}$ divided by the conformational partition function of $\mathcal{S}$. The conformational partition function of a protein may be crudely approximated in the high temperature expansion.

$$\begin{aligned} &\log P(\mathcal{C}|\mathcal{S}) \\ &\quad \simeq \; -\frac{1}{kT}\mathcal{E}(\mathcal{C},\mathcal{S}) \\ &\qquad -[\log(\sum_{\mathcal{C}\in\{\text{compact}\}} 1) - \frac{1}{kT}\langle\mathcal{E}(\mathcal{C},\mathcal{S})\rangle_{\text{compact},T\to\infty}] \end{aligned} \tag{S1-1}$$

where k is the Boltzmann constant, T is temperature, and $\mathcal{E}(\mathcal{C},\mathcal{S})$ is the conformational free energy of the conformation $\mathcal{C}$ taken by the sequence $\mathcal{S}$. First, the sum over conformations $\mathcal{C}$ are approximated by the sum over compact/nativelike conformations whose energies are significantly lower than those of extended conformations. Then, the logarithm of the partition function is approximated by the sum of the first and the second terms in the high temperature expansion. Thus, the relative stability of the native conformation of sequence $\mathcal{S}'$ to that of sequence $\mathcal{S}$ is estimated by

$$\begin{aligned} &\log[P(\mathcal{C}|\mathcal{S}')/P(\mathcal{C}|\mathcal{S})] \\ &\quad \simeq \; -\frac{1}{kT}(\mathcal{E}(\mathcal{C},\mathcal{S}') - \mathcal{E}(\mathcal{C},\mathcal{S}))\ , \\ &\qquad \text{if the amino acid composition does not change.} \end{aligned} \tag{S1-2}$$

The mean energy of compact conformations does not depend on the details of the amino acid order in protein sequences but primarily on the amino acid composition. Therefore, if the amino acid composition keeps constant during amino acid substitutions, as indicated by the present assumption of the stationary state for amino acid substitutions, the relative stability can be approximated by the difference of the native conformational energies of the two sequences.

As a result, the parameter w_{ab}, whose exponent is the acceptance rate of substitutions between amino acids of type a and type b, is evaluated here to be proportional to the mean free energy increment caused by a substitution between amino acids of type a and type b. Then, the mean free energy increment is approximated by the sum of two terms one of which results from the increment of contact energy between amino acids in a protein structure and the other from the change of side-chain volume.

$$w_{ab} \;=\; -\beta[\Delta\hat{\varepsilon}^{\text{c}}_{ab} + \Delta\hat{\varepsilon}^{\text{v}}_{ab}] + w_0(1-\delta_{ab}) \tag{S1-3}$$

where β is a parameter, $\Delta\hat{\varepsilon}^{c}_{ab}$ is the mean increment of contact energy between amino acids due to an amino acid exchange between amino acids of type a and type b in a protein structure, and $\Delta\hat{\varepsilon}^{v}_{ab}$ is the

mean increment of free energy caused by the change of side-chain volume between amino acids of type a and type b. The exponent of the constant term, e^{w_0}, may represent the ratio of replaceable amino acid sites in a protein sequence, and then the first term represents the ratio of neutral substitutions at such mutable sites; the ratio of nonsynonymous to synonymous mutations is primarily determined by w_0. However, w_0 may be positive, meaning positive selection.

Mean energy increment for each type of amino acid substitutions

To consider the mean contact energy increment due to an amino acid replacement between amino acids of type a and type b, we must note that the evolutionary process of amino acid substitutions in proteins is assumed here to be in the stationary process, which means that the amino acid composition of proteins must be kept constant in the whole process of amino acid substitutions. To keep the amino acid composition constant, an exchange of amino acids in a protein may be considered as the process of substitutions. A mean contact energy increment, $2\Delta\varepsilon^c_{ab}$, due to an exchange between amino acids of type a and type b in a protein can be estimated [17] by averaging the difference of interaction energies over surrounding residues as

$$\Delta\hat{\varepsilon}^{\mathrm{c}}_{ab} = \Delta\hat{\varepsilon}^{\mathrm{c}}_{ba} = \sum_c (e_{bc} - e_{ac})(\frac{N_{ac}}{N_a} - \frac{N_{bc}}{N_b}) \geq 0 \qquad \text{(S1-4)}$$

where $e_{ac}(= e_{ca})$ is the contact energy between amino acids of type a and c, and $N_{ac}(= N_{ca})$ is a half of the observed number of contacts between amino acids of type a and type c, and N_a is the number of amino acids of type a in protein structures. The contact energies e_{ab} and the number of contacts N_{ab} are the ones evaluated from the numbers of contacts between amino acids observed in representative protein structures [46]. The mean energy increment due to an amino acid exchange is non-negative for any pair of amino acids [17], because the contact energies are derived by assuming that the native conformations of proteins are at the minimum of the total contact energy. This means that no favorable substitutions occur in protein evolution in which amino acid substitutions are in the stationary state. Thus, the assumption of the stationary state for amino acid substitutions is consistent [46] with the neutral theory [47] of molecular evolution.

A contact potential used is a statistical estimate [46] of contact energies with a correction [48] for the Bethe approximation [49, 50]. The contact energy between amino acids of type a and type b was estimated as

$$e_{ab} = e_{rr} + \alpha'[\Delta e^{\mathrm{Bethe}}_{ar} + \Delta e^{\mathrm{Bethe}}_{rb} + \frac{\beta'}{\alpha'}\delta e^{\mathrm{Bethe}}_{ab}] \qquad \text{(S1-5)}$$

e_{rr} is part of contact energies irrespective of residue types and is called a collapse energy, which is essential for a protein to fold by cancelling out the large conformational entropy of extended conformations but cannot be estimated explicitly from contact frequencies between amino acids in protein structures. $\Delta e^{\mathrm{Bethe}}_{ar}$ and $\delta e^{\mathrm{Bethe}}_{ab}$ are the values of Δe_{ar} and δe_{ab} evaluated by the Bethe approximation from the observed numbers of contacts between amino acids. $\Delta e_{ar} + e_{rr}$ is a partition energy or hydrophobic energy for a residue of type a. δe_{ab} is an intrinsic contact energy for a contact between residues of type a and type b; refer to [48, 50] for their exact definitions. The proportional constants for correction were estimated as $\beta'/\alpha' = 2.2$ and $\alpha' \leq 1$ [48]. Here, energy is measured in kT units. The scaling constant β in Eq. S1-3 in the text is given for $\alpha' = 1$.

The energy increment $\Delta\hat{\varepsilon}^v_{ab}$, which results from a replacement between amino acids of different sizes, is assumed here to be proportional to the volume difference between amino acids of the type a and type b:

$$\Delta\hat{\varepsilon}^{\mathrm{v}}_{ab} = \upsilon\,[\frac{\sum_{a,b}\Delta\hat{\varepsilon}^{\mathrm{c}}_{ab}}{\sum_{a,b}|V_a - V_b|}]|V_a - V_b| \qquad \text{(S1-6)}$$

where V_a is the volume of amino acid a, and v is a proportional constant. The value of v is taken to be equal to one, otherwise specified; that is, the contact energy increment and the volume change are assumed to contribute to the total free energy increment and the acceptance rate with an equal weight. The amino acid volumes used here are the mean volume occupied by each type of amino acid in protein structures, and taken from the set named BL+ in Table 6 of Tsai et al. [51]; the volume of a half cystine (labeled as "cys" in the table) is used here for a cysteine.

The values of $[\Delta\hat{\varepsilon}^{\mathrm{c}}_{ab} + \Delta\hat{\varepsilon}^{\mathrm{v}}_{ab}]$ for all amino acid pairs are provided in Supporting Information, Data S1.

Supplementary Results

Models with no amino acid dependences of selective constraints

Before examining the effects of selective constraints (w_{ab}) on likelihood, ML values for the models with no amino acid dependences of selective constraints, i.e., $\beta = 0$ in Eq. 11, were calculated for JTT, WAG, cpREV, and mtREV. The ΔAIC value and the ML estimators of $m_{\xi\eta}$, f_{ξ}^{mut}, f_{ξ}^{usage}, and σ for each model are listed in Table 2 and Table S1, respectively. Please note that w_0 is fixed here to 0, and so there is completely no selection pressure on nonsynonymous replacements; the likelihoods of amino acid substitution matrices do not strongly depend on w_0 and codon substitution data are required to reliably estimate the value of w_0. ML parameters in each model are specified by the parameter id numbers written in the parenthesis in the second column; each id number corresponds to the parameter id number listed in Table 3. Each model is called the No-Constraints model with a suffix meaning the number of ML parameters; see Table 1. Although No-Constraints models corresponding to the Kimura's two-parameter model [1], the model of Hasegawa et al. [2], the Tamura-Nei model [3] and the general reversible model [52] were examined, only three models for each matrix are shown in Table 2.

The bias toward transition has been often pointed out [53]. In the present results for the No-Constraints models, tthe ratio of transition to transversion exchangeability $m_{tc|ag}/m_{[tc][ag]}$ is evaluated to be between 1.5 and 3.3 for all four matrices of JTT, WAG, cpREV, and mtREV, although that for mtREV is larger than those for the others. For the No-Constraints-1 of mtREV, its parameter is evaluated to be $\hat{m}_{tc|ag}/\hat{m}_{[tc][ag]} = 2.32$ and the ratio of the total transition to the total transversion rate is equal to 1.24. This estimate of transition to transversion exchangeability bias for mitochondrial proteins is significantly smaller than the previous estimate by a maximum likelihood method for phylogeny. Yang et al. [7] estimated $\hat{m}_{tc|ag}/\hat{m}_{[tc][ag]} = 9.157$ for the model corresponding to the No-Constraints-1 in the analyses of the most likely phylogeny of mitochondrial DNA encoding proteins.

Although the significance of each parameter is indicated by the AIC values of the No-Constraints models with the various sets of parameters, its discussion is postponed until the next section where results for models with selective constraints are presented, because no selective constraints on amino acids is a completely wrong assumption.

A physico-chemical evaluation of selective constraints on amino acids

Let us examine how the likelihood of JTT is improved by using the present formula for selective constraints, Eq. 11. The first evaluation of selective constraints on amino acids is based on the mean energy increments due to an amino acid replacement that result from the changes of pairwise contact energies [17, 46, 48, 49] and the volume change [51] of an amino acid side chain by an amino acid replacement. This model in which selective constraints on amino acids are evaluated from mean energy increments due to an amino acid replacement is called here an Energy-Increment-based (EI) model with a suffix meaning the number of ML parameters; see Table 1. The ML values for the EI models with various sets of parameters are listed in Table 2, and the ML estimates for the EI-10 and the EI-11 are listed in Table S2.

The No-Constraints-1, the No-Constraints-10, and the No-Constraints-13 models correspond to a special case of $\beta = 0$ in the EI-2, the EI-11, and the EI-14 models, respectively. As a matter of course, the

selective constraints on amino acids that represent conservative selection against amino acid substitutions significantly improve the ΔAIC values for all substitution matrices.

The significance of multiple nucleotide changes in a codon is indicated by the improvements of the ΔAIC between the EI-3 and the EI-4, between the EI-12 and the EI-13M, between the EI-10 and the EI-11, and between the EI-13 and the EI-14 models, in the latter of which the parameter $\hat{m}_{[tc][ag]}$ for multiple nucleotide changes is optimized as a free variable. Also, the ΔAIC is improved by the inclusion of the scale parameter σ; compare the ΔAIC values between the EI-2 and the EI-3, between the EI-10M and the EI-11, between the EI-12 and the EI-13, and between the EI-13M and the EI-14. Thus, taking account of both multiple nucleotide changes in a codon and variations in substitution rates is essential to obtain the reasonably large ML values.

The most effective one of the remaining parameters on likelihood is the parameter for transition-transversion bias, $m_{tc|ag}/m_{[tc][ag]}$. The next effective parameters are f_ξ^{mut} and f_ξ^{usage}, and finally the remaining rate parameters. The ΔAIC values of the models EI-2G, EI-3, EI-7, EI-11, EI-10MU, and EI-14 indicate that all parameters are effective to significantly improve the likelihood of each of the observed matrices. The ML estimates of the parameters f_ξ^{mut} and f_ξ^{usage} show the similar tendencies between the models, although this tendency differs among the substitution matrices, JTT, WAG, cpREV, and mtREV. The comparison of the ΔAIC values between the EI-10MU and the EI-14 models indicates that the parameters for exchangeabilities except for transition-transversion bias, are statistically significant but are not so effective as f_ξ^{mut} and f_ξ^{usage} on the improvement of the likelihood.

The relative weight υ of the effects of volume change due to an amino acid replacement on selective constraints in Eq. S1-S1-6 is assumed to be equal to one but may be varied. Optimizing υ as a free variable can improve the value of ΔAIC from 13151.9 to 12932.1 for JTT. This model may be justified because the effects of volume change due to an amino acid replacement on protein structures may be different among the types of protein structures, i.e., between membrane and soluble proteins, and between α and β proteins.

Table 2 shows that the parameters $\{f_\xi^{\text{usage}}\}$ for codon usage are significant to improve likelihood, however, the ML estimator of f_ξ^{usage} often takes extremely small or large values. Thus, it may be better to assume equal codon usage by fixing $f_\xi^{\text{usage}} = 0.25$ if codon frequencies are unknown. In the following, equal codon usage is assumed in most cases of unknown codon frequencies.

Other physico-chemical evaluations of selective constraints on amino acids

Grantham [31] and Miyata et al. [32] introduced physico-chemical distances between amino acids in attempts to model selective restraints against amino acid substitutions. Their physico-chemical distances were also used by Goldman and Yang [18] and Yang et al. [7], in which the acceptance ratio ($\exp w_{ab}$) was represented by using a linear formula of Miyata et al. [32] ($\exp w_{ab} = \alpha(1 - \beta d_{ab})$) or a geometric formula ($\exp w_{ab} = \alpha \exp(-\beta d_{ab})$) of physico-chemical distance d_{ab} between amino acids of type a and b; where α and β are parameters. In their models, stepwise substitutions through single nucleotide changes were assumed, and codon substitutions due to multiple nucleotide changes were completely neglected; in other words, $m_{[tc][ag]} \rightarrow 0$ with $m_{\xi\eta}/m_{[tc][ag]} = \text{constant}$ in Eq. 1 was assumed. Yang et al. [7] reported that the use of the Miyata's distance [32] for the acceptance ratio in their codon-based model lead to a better fit to the small data of mitochondrial protein sequences than the JTT-F and the mtREV24-F models, in which the rate matrix of JTT or mtREV24 with an adjustment for the equilibrium frequencies of amino acids is used; their codon-based models correspond to the present model with $m_{[tc][ag]} \rightarrow 0$, $m_{tc} = m_{ag}$, and $m_{ta} = m_{tg} = m_{ca} = m_{cg}$, i.e., the two parameter model for nucleotide mutations with the adjustment for amino acid frequencies.

Table 2 and Table S3 list the ML values and the ML estimates for JTT and WAG in the present

models in which either the Grantham's distance or the Miyata's distance (d_{ab} for an amino acid pair a and b) is used as $w_{ab}^{\text{estimate}} = -d_{ab}$ to evaluate the selective constraints w_{ab} in Eq. 11;

$$w_{ab} \equiv -\beta d_{ab} + w_0(1 - \delta_{ab}) \tag{S1-7}$$

where w_0 is always fixed to the value 0, because the likelihoods of amino acid substitution matrices do not significantly depend on w_0. These models are called here Grantham and Miyata with a suffix meaning the number of ML parameters; see Table 1. Both the selective constraints based on the Grantham's and on the Miyata's distances significantly improve the ΔAIC.

Miyata et al. [32] claimed that their new scale can explain the tendencies of amino acid replacements better than the Grantham's distance scale. Table 2 shows that the Miyata's physico-chemical distance performs better in all parameter sets than the Grantham's distance. This result is consistent with that of Yang et al. [7] for mitochondrial proteins. The present physico-chemical evaluation of selective constraints (EI model) fits JTT and WAG even better than the Miyata's distance scale, although the performances of both the methods are almost same for cpREV and mtREV.

One of the important facts in these results is that allowing multiple nucleotide changes in a codon significantly improve the AIC irrespective of the estimations of selective constraints; compare the Δ AIC values between the Grantham-10 and the Grantham-11, and between the Miyata-10 and the Miyata-11. In other words, the improvement of the AIC value is not an artifact due to the present physico-chemical estimation of selective constraints.

Evolutionary process of amino acid substitutions in terms of log-odds

Kinjo and Nishikawa [45] reported that the most principal component of log-odds matrices exhibits a sharp transition at the sequence identity of 30-35%, which almost coincides with the twilight zone in homology search. This interesting feature of log-odds matrices was found by analyzing the eigenspectra of the log-odds matrices for 18 different levels of sequence identities, which were constructed from the structure-based alignments of protein sequences in the Homstrad database [54] with the procedure of the BLOSUM substitution matrices [55]. Although they did not mention, this feature is also encoded in an amino acid or codon substitution probability matrix for a short time interval such as JTT, WAG, LG, and KHG. Here, we show that this feature is encoded in the transition matrix estimated by the ML-91+ model that precisely reproduces JTT.

Fig. S11A shows the first, the second and the third principal eigenvalues of the log-odds matrix ($\log\text{-}O(\langle S\rangle(t))_{ab}$) of the ML-91+ are drawn on amino acid identity by solid, broken and dotted lines, respectively. The dependences of these eigenvalues on the amino acid identity are almost exactly the same as those shown in the Fig. 1A of their paper [45]; i.e., the first principal eigenvalue changes its sign from negative to positive at about 35 % identity, and the second principal eigenvalue takes the place of a negative eigenvalue by changing its sign from positive to negative. A similar event of exchanging the second and the third principal eigenvalues in the order occurs between 15 and 20 % identity in their case and at about 25 % identity in the present JTT-ML91+ matrix; note that the value of sequence identity x % on the abscissa in their Fig. 1A [45] represents a log-odds matrix compiled from alignments with sequence identity $\geq x$ % and $< (x + 10)$ %.

From Fig. S11A, one infers that the vector corresponding to the first principal eigenvector at about 80 % identity becomes the second principal eigenvector at about 35 % identity and the third principal eigenvector at about 25 % identity. Likewise one infers that the vector corresponding to the second principal eigenvector at about 80 % identity becomes the first principal eigenvector below about 35 % identity, and the vector being equal to the third principal eigenvector at about 80 % identity becomes the second principal eigenvector below 25 % identity. This inference is exactly correct, as shown in Figs.

S11B, S11C, and S11D and in Fig. 1B of Kinjo and Nishikawa [45]. In Figs. S11B, S11C, and S11D, the inner product $\boldsymbol{V}_i(t) \cdot \boldsymbol{V}_j^{\mathrm{JTT}}(\mathrm{20PAM})$ of the ith principal eigenvectors $\boldsymbol{V}_i(t)$ of the JTT-ML91+ log-odds matrix at time t and the jth principal eigenvectors $\boldsymbol{V}_j^{\mathrm{JTT}}(\mathrm{20PAM})$ of the JTT log-odds matrix at 20 PAM is plotted against sequence identity at time t. Fig. S11 indicates that the eigenvalues change but the eigenvectors remain almost the same until sequence identity attains about 20 %. The sharp exchange between the first and the second principal eigenvalues is not peculiar to the present substitution matrices but can occur in any transition matrix in which diagonal elements differ from each other; transition matrices generated with $R_{ab} = \mathrm{const} \cdot f_b$ have such a characteristic feature. A critical point is what the principal eigenvectors are as well as those eigenvalues.

The first principal eigenvalues of the log-odds matrices are large negative in $t > 40$ % identity, contributing negative values to the diagonal elements of the log-odds matrices. Thus, the first principal eigenvector with a large negative eigenvalue is a primary contribution to the mutability of each amino acid, as pointed out in Kinjo and Nishikawa [45]. On the other hand, the second and the third principal eigenvalues are positive, so that the product of ith and jth elements of their eigenvectors represents how often the ith and the jth types of amino acids can be replaced to each other. Kinjo and Nishikawa [45] showed that the second principal eigenvector is well correlated with a hydrophobicity scale of amino acids.

Thus, the sharp transition in the order of the eigenvalues contributing to the mutabilities of amino acids and to the replaceabilities of amino acid pairs at about 35 % identity means that the memory of ancestral sequences disappear and amino acids in the sequences are replaced with similar physico-chemical types of amino acids at about 35 % identity. This explains why it becomes hard to identify homologous relationships between sequences whose similarities are less than 35 % identity [45]. Barriers for identifying sequence homologies may also exist at about 25 % and 15 %, where the second and the third sharp transitions in the order of the eigenvalues occur. Because conservative substitutions in respect to physico-chemical properties of amino acids are required for proteins to fold into their native structures, the second barrier at about 25 % corresponds to a threshold for being able to detect structural homology between proteins. The similar characteristic features are observed in the mtREV and the cpREV matrices, too. Thus, the characteristic features becoming manifest after a long evolutionary history of proteins are completely encoded in the transition matrices based on the reversible Markov model. This fact supports in some extent the appropriateness of the present Markov model to describe the evolutionary process of codon substitutions.

References

1. Kimura M (1980) A simple model for estimating evolutionary rates of base substitutions through comparative studies of nucleotide sequences. J Mol Evol 16: 111-120.

2. Hasegawa M, Kishino H, Yano T (1985) Dating of the human-ape splitting by a molecular clock of mitochondrial dna. J Mol Evol 22: 160-174.

3. Tamura K, Nei M (1993) Estimation of the number of nucleotide substitutions in the control region of mitochondrial dna in humans and chimpanzees. Mol Biol Evol 10: 512-526.

4. Dayhoff MO, Schwartz RM, Orcutt BC (1978) A model of evolutionary change in proteins. In: Dayhoff MO, editor, Atlas of protein sequence and structure, Washington D.C.: National Biomedical Research Foundation, volume 5. Suppl. 3 edition, pp. 345-352.

5. Jones DT, Taylor WR, Thornton JM (1992) The rapid generation of mutation data matrices from protein sequences. CABIOS 8: 275-282.

6. Adachi J, Hasegawa M (1996) Model of amino acid substitution in proteins encoded by mitochondrial dna. J Mol Evol 42: 459-468.

7. Yang Z, Nielsen R, Hasegawa M (1998) Models of amino acid substitution and application to mitochondrial protein evolution. Mol Biol Evol 15: 1600-1611.

8. Adachi J, Waddell PJ, Martin W, Hasegawa M (2000) Plastid genome phylogeny and a model of amino acid substitution for proteins encoded by chloroplast dna. J Mol Evol 50: 348-358.

9. Dimmic MW, Mindell DP, Goldstein RA (2000) Modelling evolution at the protein level using an adjustable amino acid fitness model. Pacific Symposium on Biocomputing 5: 18-29.

10. Whelan S, Goldman N (2001) A general empirical model of protein evolution derived from multiple protein families using a maximum-likelihood approach. Mol Biol Evol 18: 691-699.

11. Le SQ, Gascuel O (2008) An improved general amino acid replacement matrix. Mol Biol Evol 25: 1307-1320.

12. Huelsenbeck JP, Joyce P, Lakner C, Ronquist F (2008) Bayesian analysis of amino acid substitution models. Phil Trans R Soc B 363: 3941-3953.

13. Schneider A, Cannarozzi GM, Gonnet GH (2005) Empirical codon substitution matrix. BMC Bioinformatics 6: 134.

14. Kosiol C, Holmes I, Goldman N (2007) An empirical codon model for protein sequence evolution. Mol Biol Evol 24: 1464-1479.

15. Delport W, Scheffler K, Gravenor MB, Muse SV, Kosakovsky Pond S (2010) Benchmarking multi-rate codon models. PLos One 5: e11587.

16. Delport W, Scheffler K, Botha G, Gravenor MB, Muse SV, et al. (2010) Codontest: Modeling amino acid substitution preferences in coding sequences. PLos Comp Biol 6: e1000885.

17. Miyazawa S, Jernigan RL (1993) A new substitution matrix for protein sequence searches based on contact frequencies in protein structures. Protein Eng 6: 267-278.

18. Goldman N, Yang Z (1994) A codon-based model of nucleotide substitution for protein-coding dna. Mol Biol Evol 11: 725-736.

19. Muse SV, Gaut BS (1994) A likelihood approach for comparing synonymous and nonsynonymous nucleotide substitution rates, with application to the chloroplast genome. Mol Biol Evol 11: 715-724.

20. Whelan S, Goldman N (2004) Estimating the frequency of events that cause multiple-nucleotide changes. Genetics 167: 2027-2043.

21. Doron-Faigenboim A, Pupko T (2007) A combined empirical and mechanistic codon model. Mol Biol Evol 24: 388-397.

22. Yang Z, Nielsen R (2008) Mutation-selection models of codon substitution and their use to estimate selective strengths on codon usage. Mol Biol Evol 25: 568-579.

23. Seo TK, Kishino H (2008) Synonymous substitutions substantially improve evolutionary inference from highly diverged proteins. Syst Biol 57: 367-377.

24. Seo TK, Kishino H (2009) Statistical comparison of nucleotide, amino acid, and codon substitution models for evolutionary analysis of protein-coding sequences. Syst Biol 58: 199-210.

25. Jin L, Nei M (1990) Limitations of the evolutionary parsimony method of phylogeny analysis. Mol Biol Evol 7: 82-102.

26. Yang Z (1993) Maximum-likelihood estimation of phylogeny from dna sequences when substitution rates differ over time. Mol Biol Evol 10: 1396-1401.

27. Averof M, Rokas A, Wolfe KH, Sharp PM (2000) Evidence for a high frequency of simultaneous double-nucleotide substitutions. Science 287: 1283-1286.

28. Smith NGC, Webster MT, Ellegren H (2003) A low rate of simultaneous double-nucleotide mutations in primates. Mol Biol Evol 20: 47-53.

29. Bazykin G, Kondrashov F, Ogurtsov A, Sunyaev S, Kondrashov A (2004) Positive selection at sites of multiple amino acid replacements since rat-mouse divergence. Nature 429: 558-562.

30. Anisimova M, Kosiol C (2009) Investigating protein-coding sequence evolution with probabilistic codon substitution models. Mol Biol Evol 26: 255-271.

31. Grantham R (1974) Amino acid difference formula to help explain protein evolution. Science 185: 862-864.

32. Miyata T, Miyazawa S, Yasunaga T (1979) Two type of amino acid substitutions in protein evolution. J Mol Evol 12: 219-236.

33. Choi SC, Hobolth A, Robinson DM, Kishino H, Thorne JL (2007) Quantifying the impact of protein tertiary structure on molecular evolution. Mol Biol Evol 24: 1769-1782.

34. Conant GC, Wagner GP, Stadler PF (2007) Modeling amino acid substitution patterns in orthologous and paralogous genes. Mol Phylogenet Evol 42: 298-307.

35. Takahata N (1987) On the overdispersed molecular clock. Genetics 116: 169-179.

36. Rodrigue N, Lartillot N, Philippe H (2008) Bayesian comparisons of codon substitution models. Genetics 180: 1579-1591.

37. Akaike H (1974) A new look at the statistical model identification. IEEE Trans Autom Contr AC-19: 716-723.

38. Whelan S, de Bakker P, Quevillon E, Rodriguez N, Goldman N (2006) Pandit: an evolution-centric database of protein and associated nucleotide domains with inferred trees. Nucl Acid Res 34: D327-D331.

39. Miyata T, Yasunaga T (1980) Molecular evolution of mrna: a method for estimating evolutionary rates of synonymous and amino acid substitutions from homologous nucleotide sequences and its applications. J Mol Evol 16: 23-36.

40. Halpern AL, Bruno WJ (1998) Evolutionary distances for protein-coding sequences: modeling site-specific residue frequencies. Mol Biol Evol 15: 910-917.

41. Keller I, Bensasson D, Nichols RA (2007) Transition-transversion bias is not universal: A counter example from grasshopper pseudogenes. PLoS Genet 3: 0185-0191.

42. Larkin MA, Blackshields G, Brown NP, Chenna R, McGettigan PA, et al. (2007) Clustalw and clustalx version 2.0. Bioinformatics 23: 2947-2948.

43. Guindon S, Gascuel O (2003) Simple, fast, and accurate algorithm to estimate large phylogenies by maximum likelihood. Systematic Biol 52: 696-704.

44. Yang Z (1994) Maximum likelihood phylogenetic estimation from dna sequences with variable rates over sites: approximate methods. J Mol Evol 39: 306-314.

45. Kinjo AR, Nishikawa K (2004) Eigenvalue analysis of amino acid substitution matrices reveals a sharp transition of the mode of sequence conservation in proteins. Bioinformatics 20: 2504-2508.

46. Miyazawa S, Jernigan RL (2003) Long- and short-range interactions in native protein structures are consistent/minimally frustrated in sequence space. Proteins 50: 35-43.

47. Kimura M, Ohta T (1974) On some principles governing molecular evolution. Proc Natl Acad Sci USA 71: 2848-2852.

48. Miyazawa S, Jernigan RL (1999) Self-consistent estimation of inter-residue protein contact energies based on an equilibrium mixture approximation of residues. Proteins 34: 49-68.

49. Miyazawa S, Jernigan RL (1996) Residue-residue potentials with a favorable contact pair term and an unfavorable high packing density term for simulation and threading. J Mol Biol 256: 623-644.

50. Miyazawa S, Jernigan RL (1985) Estimation of effective interresidue contact energies from protein crystal structures: Quasi-chemical approximation. Macromolecules 18: 534-552.

51. Tsai J, Taylor R, Chothia C, Gerstein M (1999) The packing density in proteins: standard radii and volumes. J Mol Biol 290: 253-266.

52. Lanave C, Preparata G, Saccone C, Serio G (1984) A new method for calculating evolutionary substitution rates. J Mol Evol 20: 86-93.

53. Felsenstein J (2004) Inferring Phylogenies. Massachusetts: Sinauer Associates, Inc.

54. Mizuguchi K, Deane CM, Blundell TL, Overington JP (1998) Homstrad: a database of protein structure alignments for homologous families. Protein Sci 7: 2469-2471.

55. Henikoff S, Henikoff JG (1992) Amino acid substitution matrices from protein blocks. Proc Natl Acad Sci USA 89: 10915-10919.

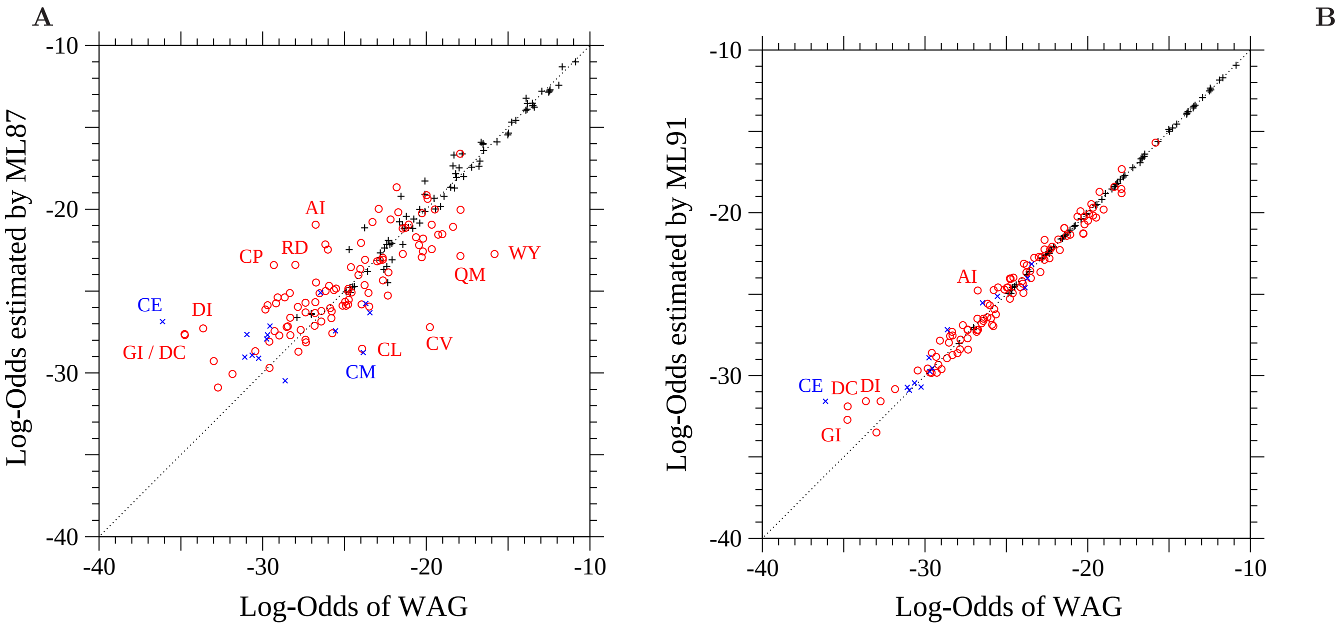

Figure S1. The ML-87 and the ML-91 models fitted to WAG. Each element log-$O(\langle S\rangle(\hat{\tau},\hat{\sigma}))_{ab}$ of the log-odds matrices of (A) the ML-87 and (B) the ML-91 models fitted to the 1-PAM WAG matrix is plotted against the log-odds log-$O(S^{\mathrm{WAG}}(1\ \mathrm{PAM}))_{ab}$ calculated from WAG. Plus, circle, and cross marks show the log-odds values for one-, two-, and three-step amino acid pairs, respectively. The dotted line in each figure shows the line of equal values between the ordinate and the abscissa.

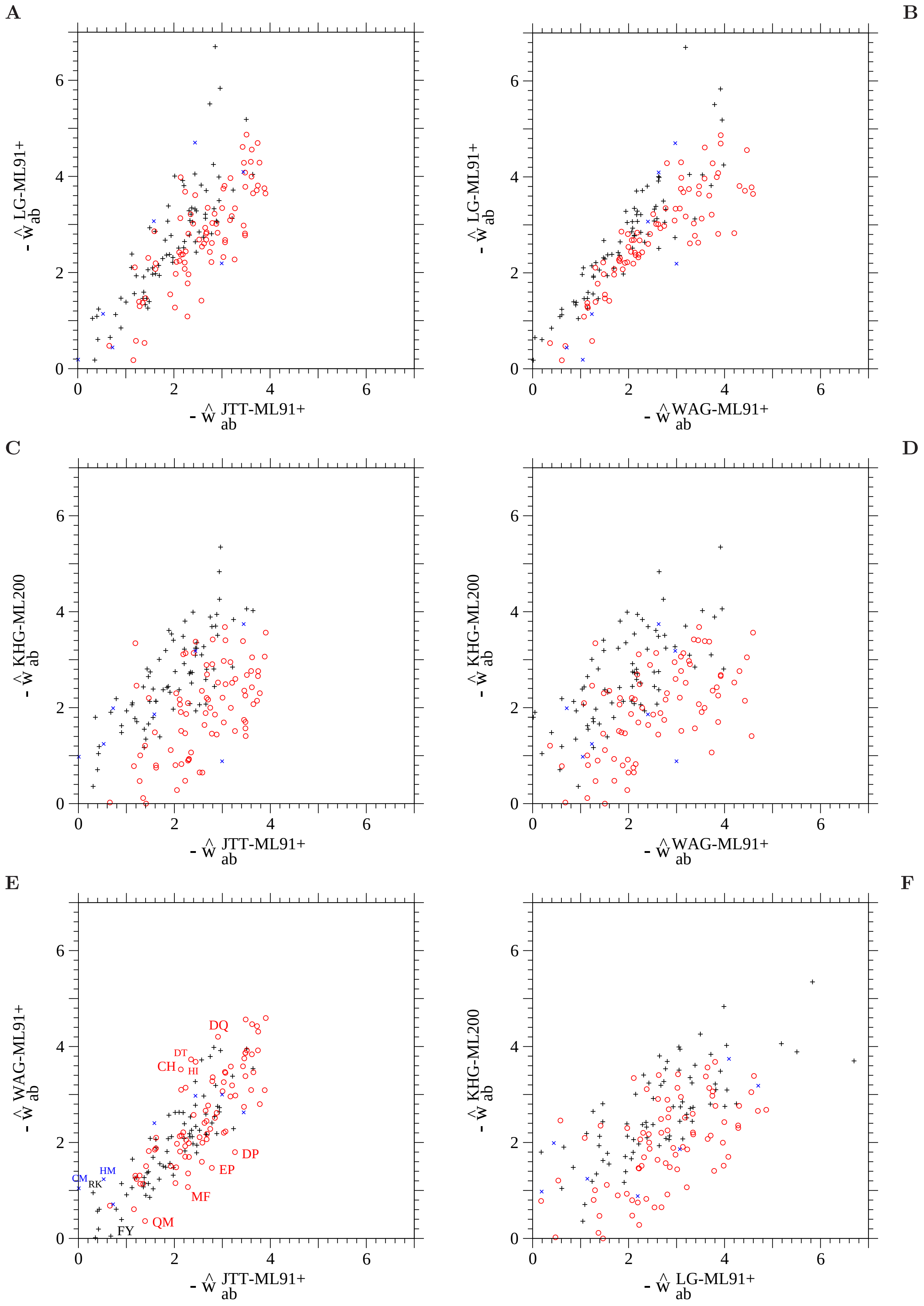

Figure S2. Comparison between various estimates of selective constraint for each amino acid pair The ML estimates of selective constraint on substitutions of each amino acid pair are compared between the models fitted to various empirical substitution matrices. The estimates $\hat{w}_{ab}$ for multi-step amino acid pairs that belong to the least exchangeable class at least in one of the models are not shown. Plus, circle, and cross marks show the values for one-, two-, and three-step amino acid pairs, respectively.

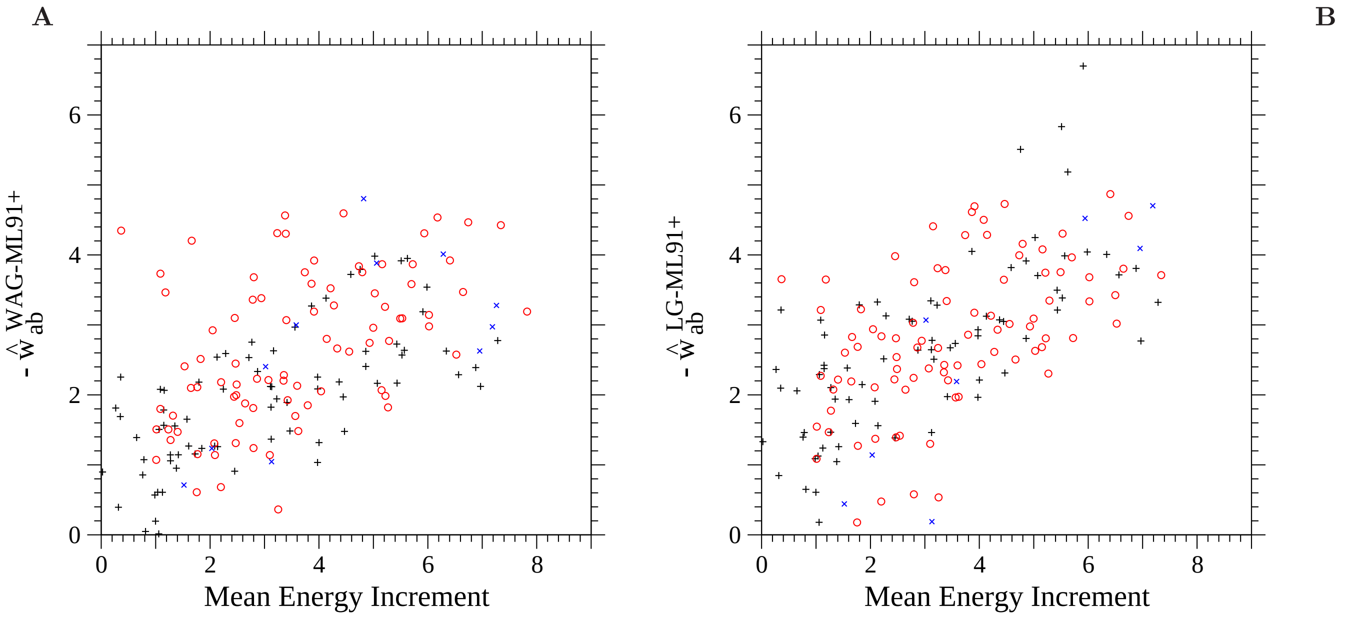

Figure S3. Selective constraint for each amino acid pair estimated from WAG and from LG. The ML estimate, $-\hat{w}_{ab}^{\text{WAG-ML91+}}$ in (A) and $-\hat{w}_{ab}^{\text{LG-ML91+}}$ in (B), of selective constraint on substitutions of each amino acid pair in the ML-91+ models fitted to the 1-PAM matrices of WAG and LG is plotted against the mean energy increment due to an amino acid substitution, $(\Delta\hat{\varepsilon}_{ab}^{c} + \Delta\hat{\varepsilon}_{ab}^{v})$ defined by Eqs. S1-4, S1-5, and S1-6. The estimates $\hat{w}_{ab}$ for the least exchangeable class of multi-step amino acid pairs are not shown. Plus, circle, and cross marks show the values for one-, two-, and three-step amino acid pairs, respectively.

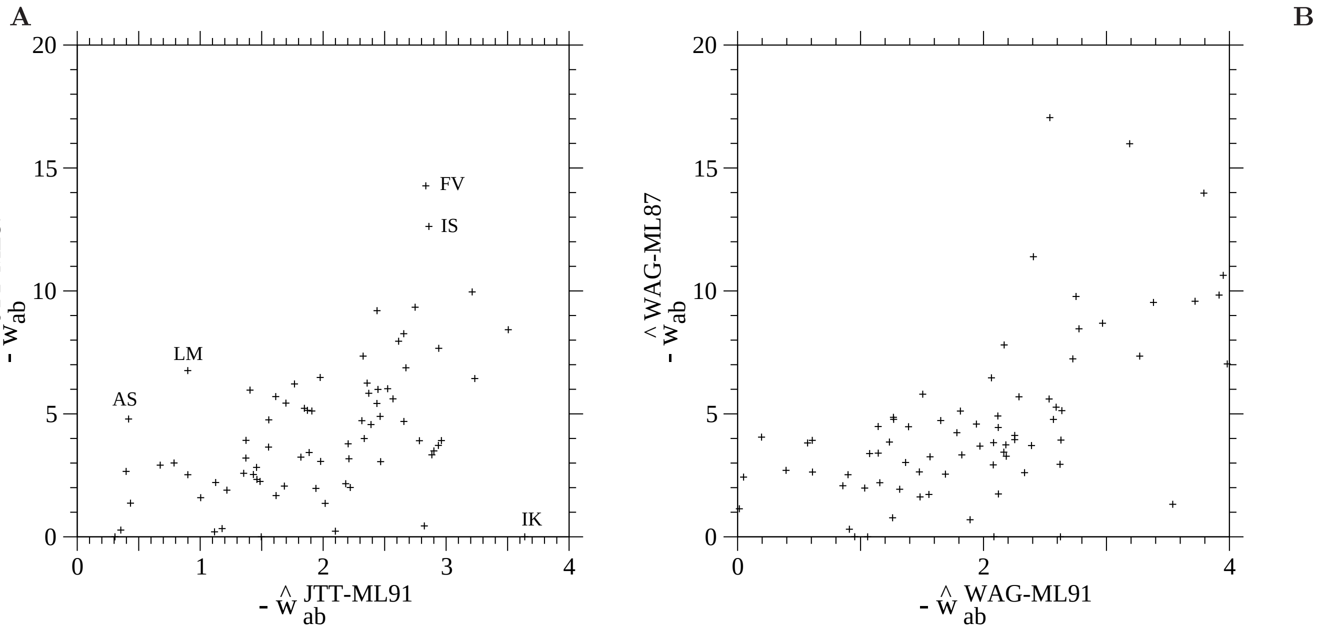

Figure S4. Comparison of the ML estimates of selective constraint for each amino acid pair between the ML-87 and the ML-91 models. The ML estimate of selective constraint for each single step amino acid pair in the ML-87 model fitted to (A) the 1-PAM JTT matrix or (B) the 1-PAM WAG matrix is plotted against that in the ML-91 model.

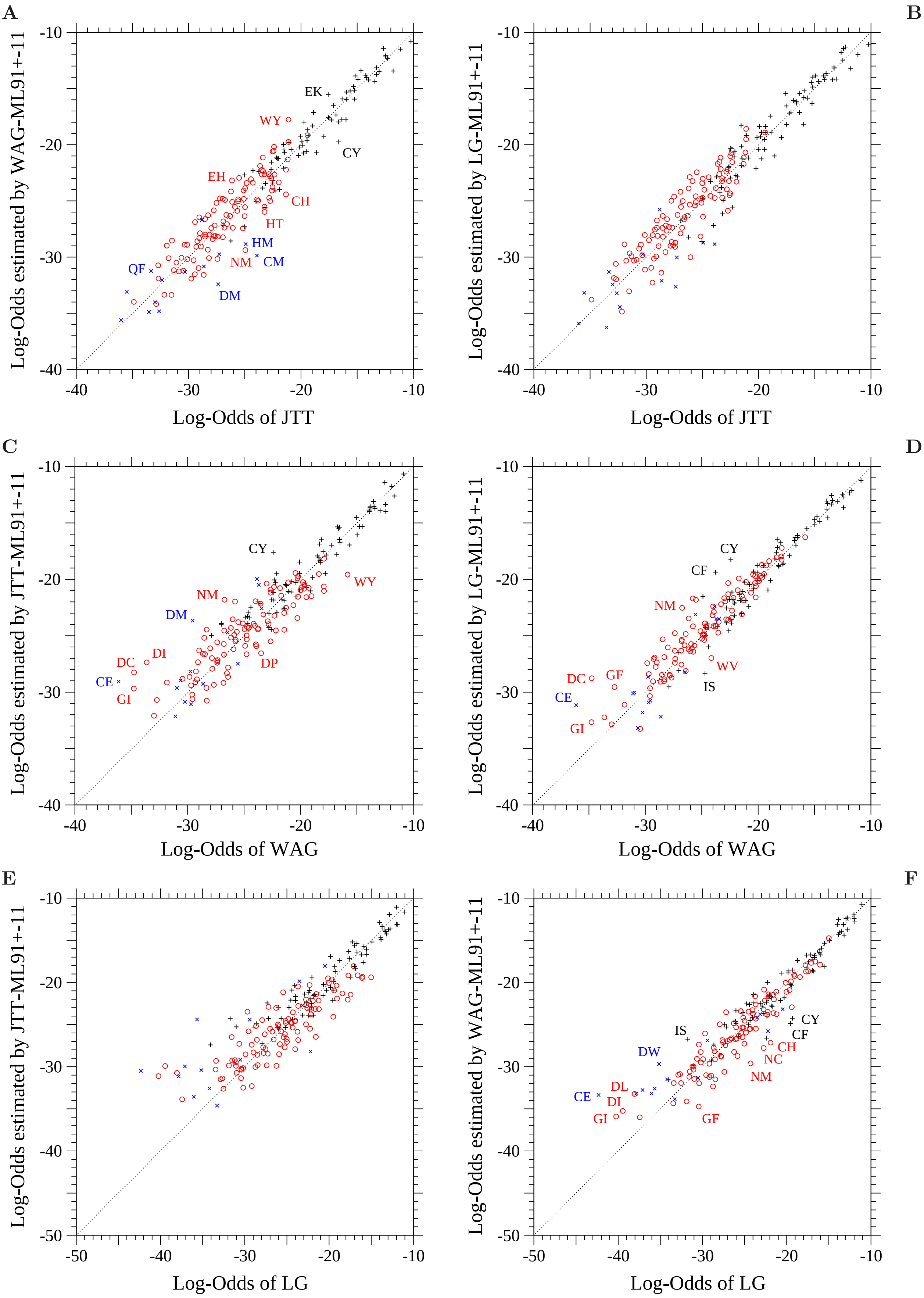

Figure S5. Models fitted to each of JTT, WAG, and LG. Each element log-$O(\langle S\rangle(\hat{\tau},\hat{\sigma}))_{ab}$ of the log-odds matrix of the model fitted to each empirical substitution matrix is plotted against the log-odds log-$O(S^{\mathrm{obs}}(1\ \mathrm{PAM}))_{ab}$ calculated from the corresponding empirical substitution matrix. Plus, circle, and cross marks show the log-odds values for one-, two-, and three-step amino acid pairs, respectively. The dotted line in each figure shows the line of equal values between the ordinate and the abscissa.

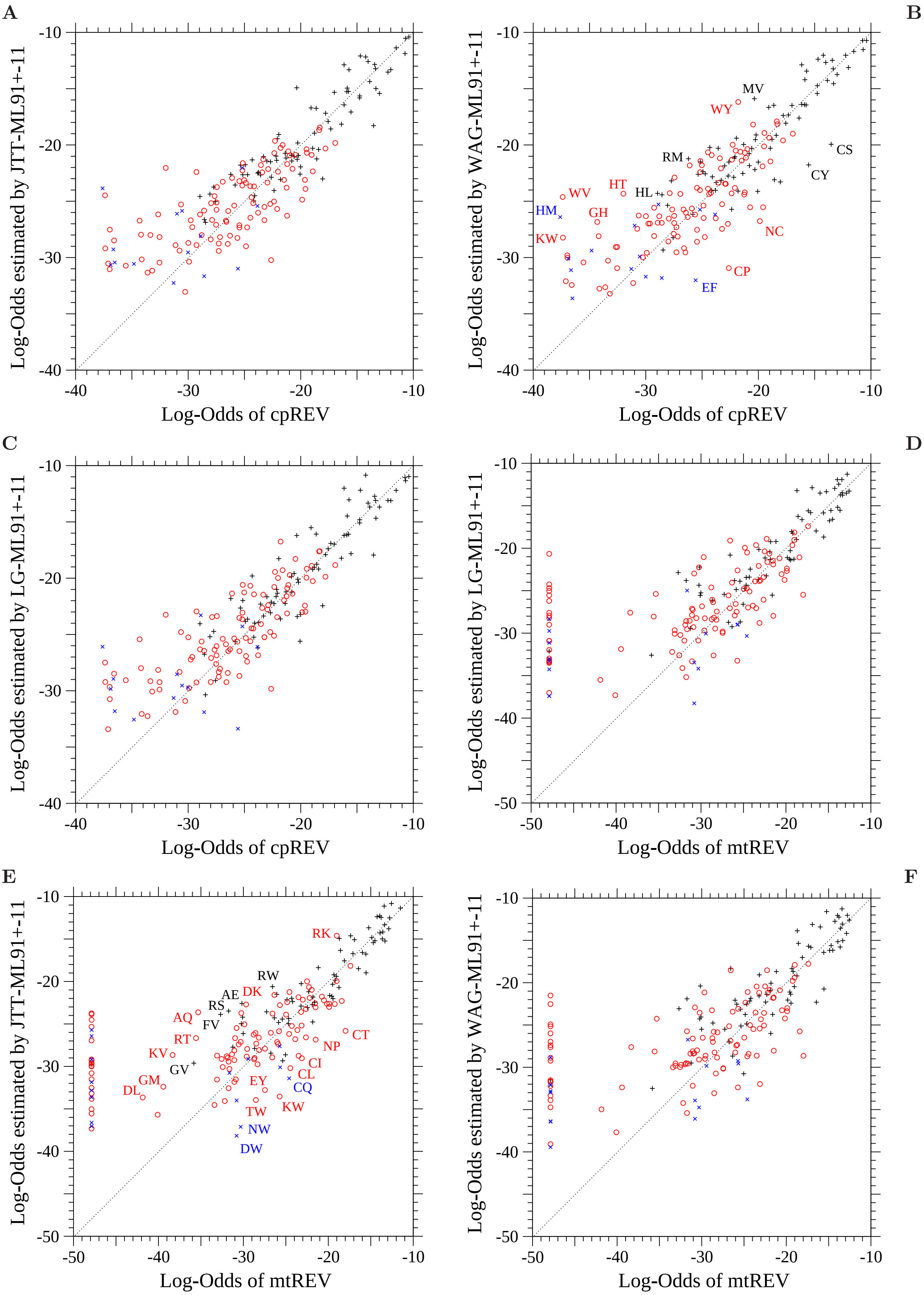

Figure S6. **Models fitted to each of cpREV and mtREV.** Each element $\log\text{-}O(\langle S\rangle(\hat{\tau},\hat{\sigma}))_{ab}$ of the log-odds matrix of the model fitted to each empirical substitution matrix is plotted against the log-odds $\log\text{-}O(S^{\text{obs}}(1\text{ PAM}))_{ab}$ calculated from the corresponding empirical substitution matrix. Plus, circle, and cross marks show the log-odds values for one-, two-, and three-step amino acid pairs, respectively. The dotted line in each figure shows the line of equal values between the ordinate and the abscissa.

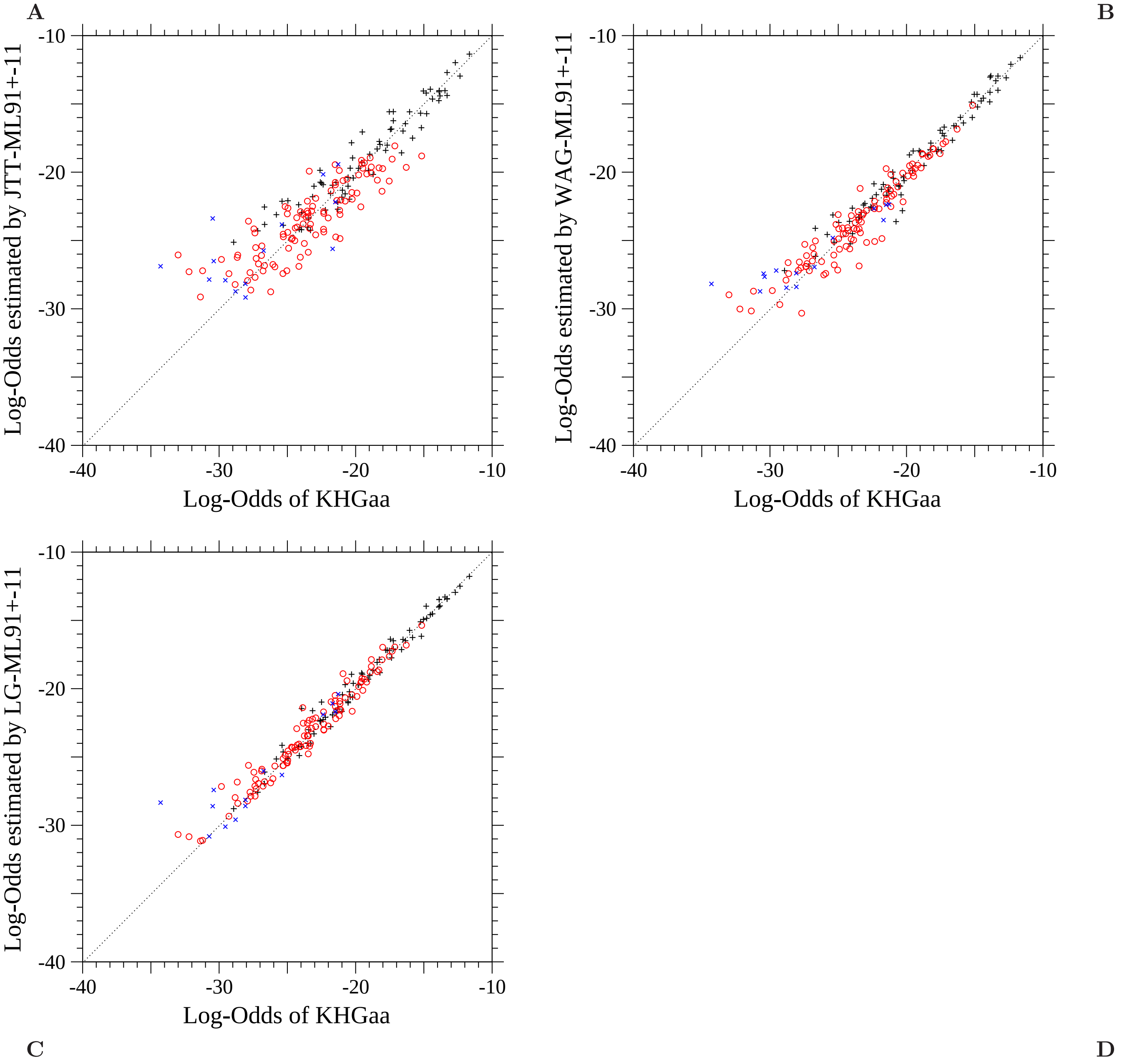

Figure S7. Models fitted to the KHG-derived amino acid substitution matrix. Each element log-$O(\langle S \rangle(\hat{\tau}, \hat{\sigma}))_{ab}$ of the log-odds matrix of the model fitted to the 1-PAM KHG-derived amino acid substitution matrix (KHGaa) is plotted against the log-odds log-$O(S^{\text{obs}}(1\ \text{PAM}))_{ab}$ calculated from KHGaa. Plus, circle, and cross marks show the log-odds values for one-, two-, and three-step amino acid pairs, respectively. The dotted line in each figure shows the line of equal values between the ordinate and the abscissa.

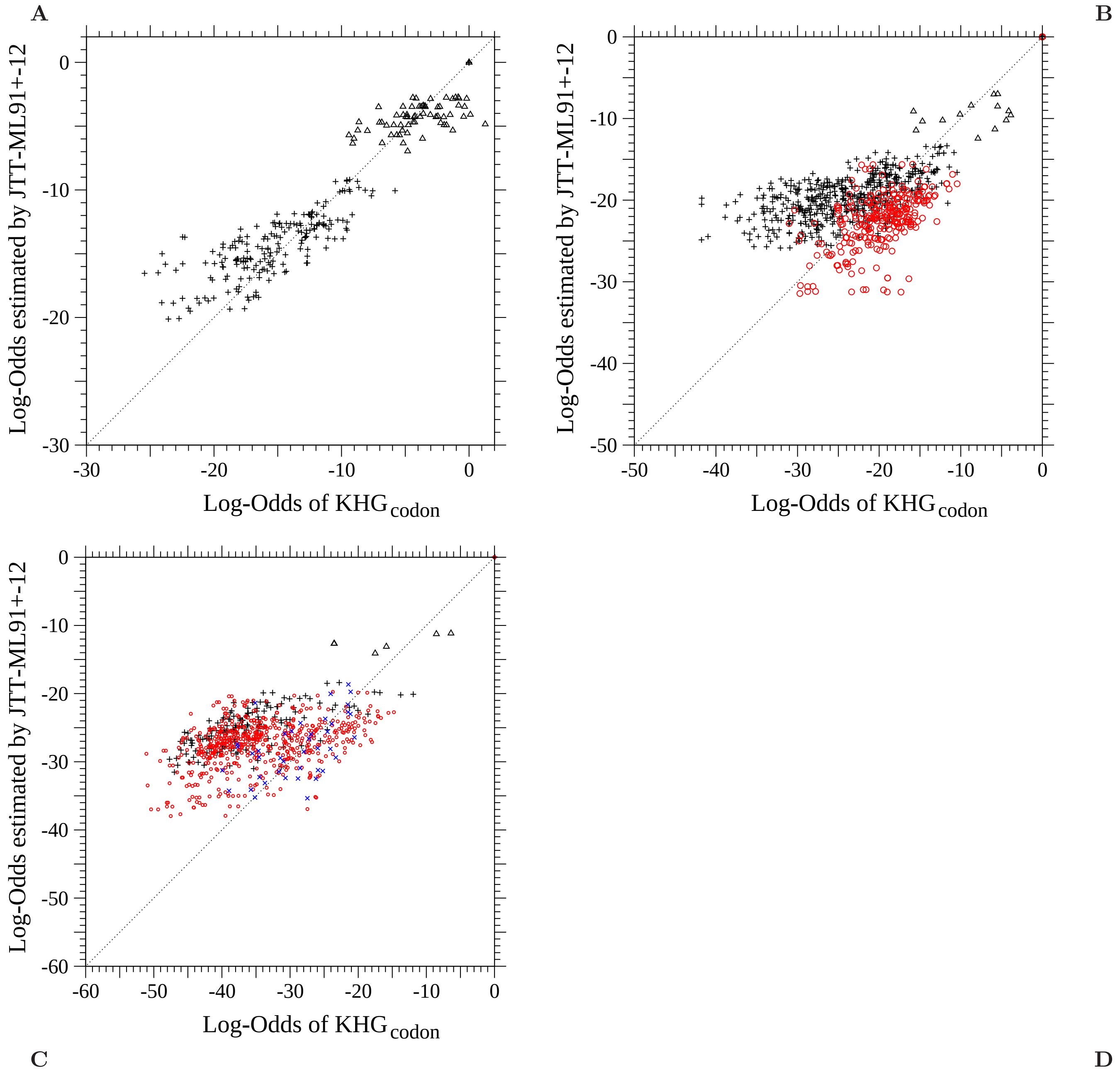

Figure S8. The JTT-ML91+-12 model fitted to the 1-PAM KHG codon substitution matrix. Each element log-$O(\langle S\rangle(\hat{\tau},\hat{\sigma}))_{\mu\nu}$ of the log-odds matrix corresponding to (A) single, (B) double, and (C) triple nucleotide changes in the JTT-ML91+-12 model fitted to the 1-PAM KHG codon substitution matrix is plotted against the log-odds log-$O(S^{\mathrm{KHG}}(1\ \mathrm{PAM}))_{\mu\nu}$ calculated from KHG. Upper triangle, plus, circle, and cross marks show the log-odds values for synonymous pairs and one-, two-, and three-step amino acid pairs, respectively. The dotted line in each figure shows the line of equal values between the ordinate and the abscissa.

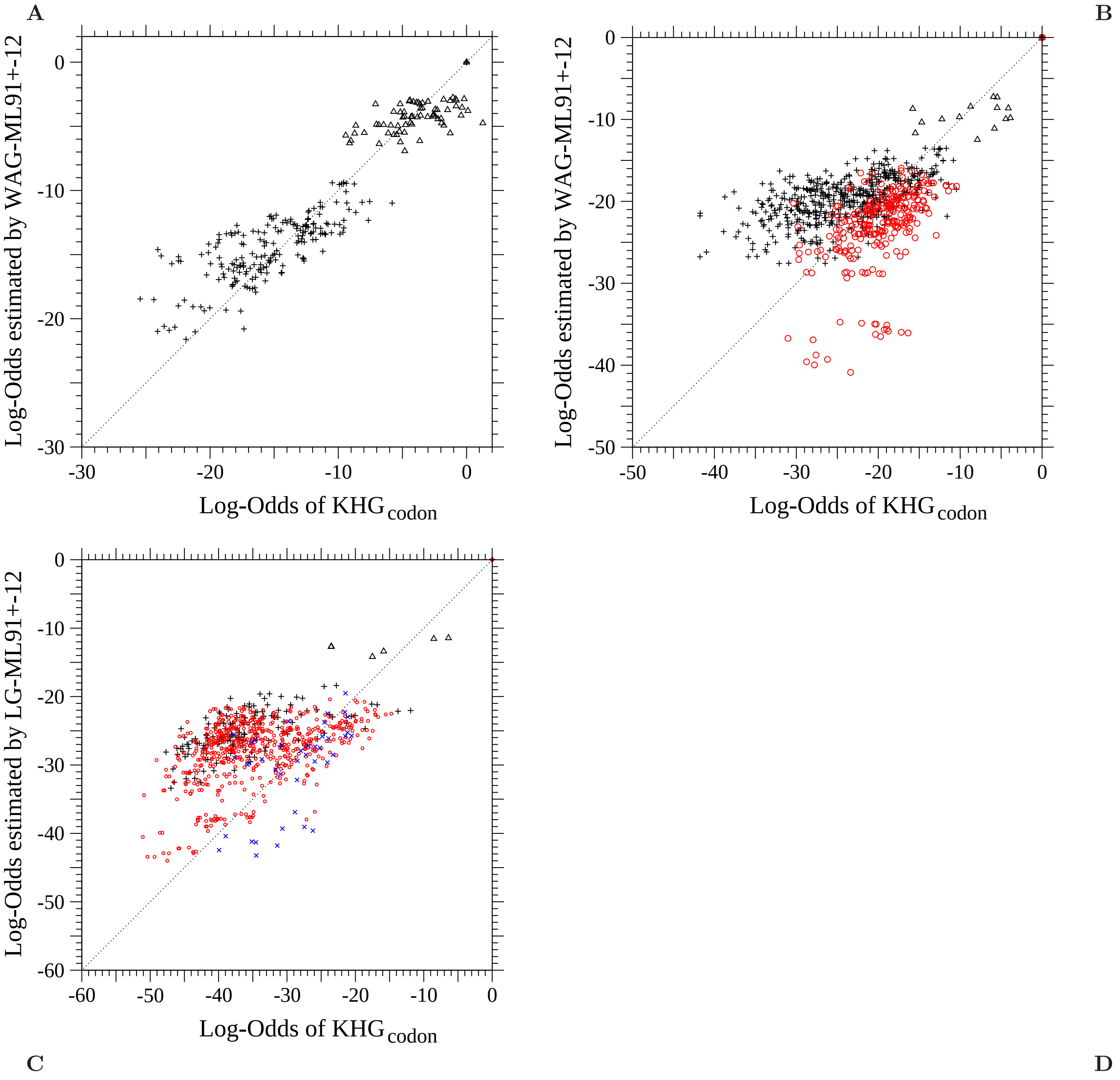

Figure S9. The WAG-ML91+-12 model fitted to the 1-PAM KHG codon substitution matrix. Each element log-$O(\langle S\rangle(\hat{\tau},\hat{\sigma}))_{\mu\nu}$ of the log-odds matrix corresponding to (A) single, (B) double, and (C) triple nucleotide changes in the WAG-ML91+-12 model fitted to the 1-PAM KHG codon substitution matrix is plotted against the log-odds log-$O(S^{\text{KHG}}(1\text{ PAM}))_{\mu\nu}$ calculated from KHG. Upper triangle, plus, circle, and cross marks show the log-odds values for synonymous pairs and one-, two-, and three-step amino acid pairs, respectively. The dotted line in each figure shows the line of equal values between the ordinate and the abscissa.

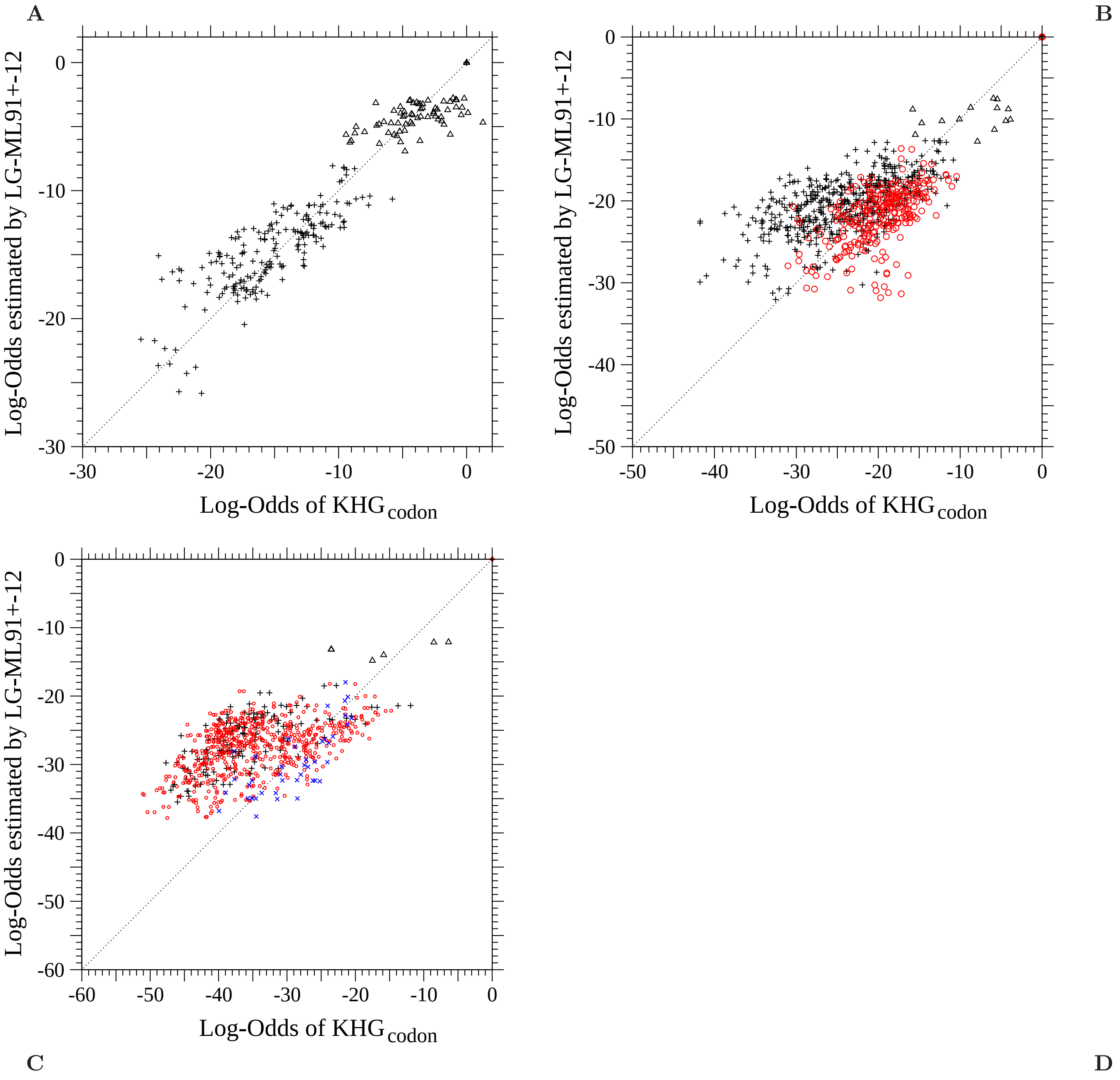

Figure S10. The LG-ML91+-12 model fitted to the 1-PAM KHG codon substitution matrix. Each element log-$O(\langle S \rangle(\hat{\tau}, \hat{\sigma}))_{\mu\nu}$ of the log-odds matrix corresponding to (A) single, (B) double, and (C) triple nucleotide changes in the LG-ML91+-12 model fitted to the 1-PAM KHG codon substitution matrix is plotted against the log-odds log-$O(S^{\text{KHG}}(1 \text{ PAM}))_{\mu\nu}$ calculated from KHG. Upper triangle, plus, circle, and cross marks show the log-odds values for synonymous pairs and one-, two-, and three-step amino acid pairs, respectively. The dotted line in each figure shows the line of equal values between the ordinate and the abscissa.

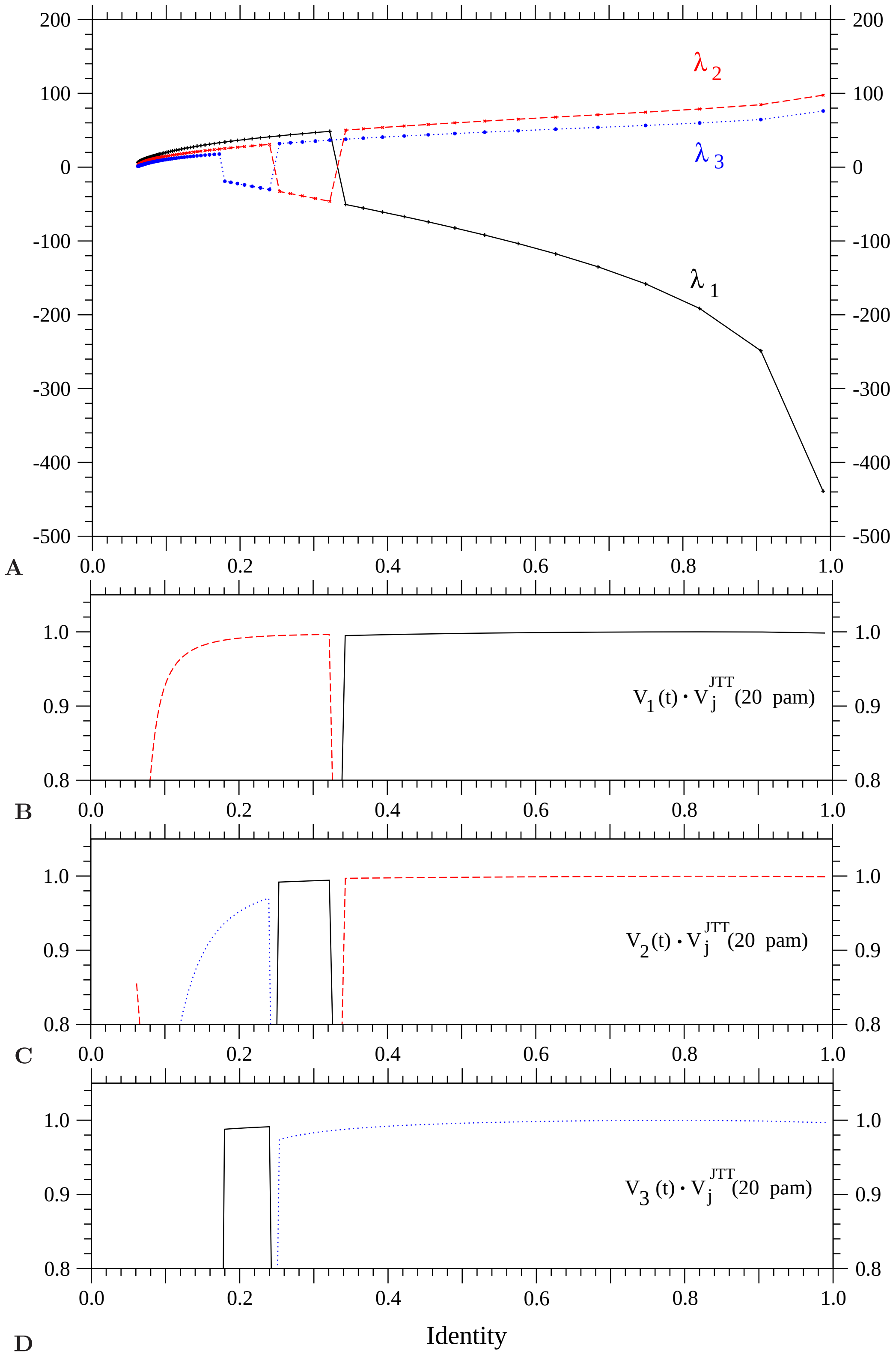

Figure S11. Temporal changes of the eigenvalues and the eigenvectors of the log-odds matrix log-$O(\langle S\rangle(t))$ calculated by the ML-91+ model fitted to JTT as a function of sequence identity. In (A), the solid, the broken, and the dotted lines show the temporal changes of the first (λ_1), the second (λ_2), and the third (λ_3) principal eigenvalues, respectively. The inner products of the eigenvectors with the eigenvectors of the JTT 20-PAM log-odds matrix, $\boldsymbol{V}_i(t) \cdot \boldsymbol{V}_j^{\mathrm{JTT}}$(20-PAM), are shown in (B) for the first principal eigenvector ($i = 1$), in (C) for the second principal eigenvector ($i = 2$), and in (D) for the third principal eigenvector ($i = 3$), by solid lines for $j = 1$, by broken lines for $j = 2$, and by dotted lines for $j = 3$.

Table S1. ML estimates of the present models without selective constraints on amino acids for the 1-PAM substitution matrices of JTT, WAG, cpREV, and mtREV.

		JTT		WAG		cpREV		mtREV	
		No-Constraints- [a]		No-Constraints- [a]		No-Constraints- [a]		No-Constraints- [a]	
id no.	parameter	1	10	1	10	1	10	1	10
0	$-\hat{w}_0$	(0.0)	(0.0)	(0.0)	(0.0)	(0.0)	(0.0)	(0.0)	(0.0)
1	$1/\hat{\beta}$	(∞)	(∞)	(∞)	(∞)	(∞)	(∞)	(∞)	(∞)
2	$\hat{m}_{[tc][ag]}$	$(\to 0)$	$\to 0$	$(\to 0)$	0.279	$(\to 0)$	0.0455	$(\to 0)$	0.0405
3	$\hat{m}_{tc\|ag}/\hat{m}_{[tc][ag]}$	2.16	2.20	1.61	1.54	2.17	2.62	2.32	3.24
4	$\hat{m}_{ag}/\hat{m}_{tc\|ag}$	(1.0)	1.28	(1.0)	1.36	(1.0)	1.50	(1.0)	1.47
5	$\hat{m}_{ta}/\hat{m}_{[tc][ag]}$	(1.0)	0.629	(1.0)	0.687	(1.0)	0.480	(1.0)	0.595
6	$\hat{m}_{tg}/\hat{m}_{[tc][ag]}$	(1.0)	0.708	(1.0)	0.622	(1.0)	0.775	(1.0)	0.373
7	$\hat{m}_{ca}/\hat{m}_{[tc][ag]}$	(1.0)	1.28	(1.0)	1.45	(1.0)	1.64	(1.0)	1.96
8	$\hat{f}^{\text{mut}}_{t+a}$	(0.5)	0.495	(0.5)	0.401	(0.5)	0.279	(0.5)	0.226
9	$\hat{f}^{\text{mut}}_{t}/\hat{f}^{\text{mut}}_{t+a}$	(0.5)	0.486	(0.5)	0.503	(0.5)	0.563	(0.5)	0.583
10	$\hat{f}^{\text{mut}}_{c}/\hat{f}^{\text{mut}}_{c+g}$	(0.5)	0.335	(0.5)	0.354	(0.5)	0.306	(0.5)	0.223
14	$\hat{\sigma}$	$(\to 0)$	1.76	$(\to 0)$	1.58	$(\to 0)$	2.96	$(\to 0)$	2.46
	$\hat{\tau}\hat{\sigma}$	0.0137	0.0228	0.0136	0.0206	0.0139	0.0296	0.0149	0.0296
	#parameters	21	30	21	30	21	30	21	30
	$\hat{I}_{KL}(\hat{\boldsymbol{\theta}}) \times 10^8$ [b]	729533	207260	1156393	233841	1014962	249448	945289	305500
	ΔAIC [c]	86428.1	24595.5	37917.6	7719.1	3478.0	904.5	2644.1	901.0
	Ratio of substitution rates								
	per codon								
	the total base/codon	1.0	1.30	1.0	1.47	1.0	1.40	1.0	1.35
	transition/transversion	1.13	1.00	0.848	0.752	1.11	1.02	1.24	1.10
	nonsynonymous/synonymous[d]	2.75	4.15	2.84	5.77	2.60	4.91	2.09	3.30
	Ratio of substitution rates								
	per codon for $\sigma \to 0$								
	the total base/codon	1.0	1.0	1.0	1.21	1.0	1.04	1.0	1.02
	transition/transversion	1.13	1.20	0.848	0.853	1.11	1.43	1.24	1.45
	nonsynonymous/synonymous[d]	2.75	2.83	2.84	4.26	2.60	3.19	2.09	2.08

[a] In all models, equal codon usage ($\hat{f}^{\text{usage}}_t = \hat{f}^{\text{usage}}_a = \hat{f}^{\text{usage}}_c = \hat{f}^{\text{usage}}_g = 0.25$) is assumed. If the value of a parameter is parenthesized, the parameter is not variable but fixed to the value specified.
[b] $\hat{I}_{KL}(\hat{\boldsymbol{\theta}}) = -(\ell(\hat{\boldsymbol{\theta}})/N + 2.98607330)$ for JTT, $-(\ell(\hat{\boldsymbol{\theta}})/N + 2.97444860)$ for WAG, $-(\ell(\hat{\boldsymbol{\theta}})/N + 2.95801048)$ for cpREV, and $-(\ell(\hat{\boldsymbol{\theta}})/N + 2.85313622)$ for mtREV; see text for details.
[c] $\Delta\text{AIC} \equiv 2N\hat{I}_{KL}(\hat{\boldsymbol{\theta}}) + 2\times$ #parameters with $N \simeq 5919000$ for JTT, $N \approx 1637663$ for WAG, $N \approx 169269$ for cpREV and $N \approx 137637$ for mtREV; see text for details.
[d] Note that these ratios are not the ratios of the rates per site but per codon; see text for details.

Table S2. ML estimates of the present models with the selective constraints based on mean energy increments due to an amino acid substitution (EI) for the 1-PAM substitution matrices of JTT, WAG, cpREV, and mtREV.

	JTT		WAG		cpREV		mtREV	
	EI-10 [a]	EI-11 [a]	EI-10 [a]	EI-11 [a]	EI-10 [a]	EI-11 [a]	EI-10 [a]	EI-11 [a]
$-\hat{w}_0$	(0.0)	(0.0)	(0.0)	(0.0)	(0.0)	(0.0)	(0.0)	(0.0)
$1/\hat{\beta}$	2.50	2.60	1.78	2.14	2.15	2.26	2.14	2.29
$\hat{m}_{[tc][ag]}$	$(\rightarrow 0)$	0.308	$(\rightarrow 0)$	0.916	$(\rightarrow 0)$	0.684	$(\rightarrow 0)$	0.737
$\hat{m}_{tc\|ag}/\hat{m}_{[tc][ag]}$	2.51	2.22	1.82	1.58	2.82	2.24	4.21	3.06
$\hat{m}_{ag}/\hat{m}_{tc\|ag}$	1.01	1.01	1.13	1.10	1.19	1.14	1.05	1.01
$\hat{m}_{ta}/\hat{m}_{[tc][ag]}$	1.02	1.07	1.26	1.22	0.992	1.14	1.48	1.44
$\hat{m}_{tg}/\hat{m}_{[tc][ag]}$	1.06	1.09	0.985	1.01	1.34	1.23	0.792	0.797
$\hat{m}_{ca}/\hat{m}_{[tc][ag]}$	0.937	0.891	1.04	0.949	0.974	0.925	1.17	1.08
$\hat{f}^{\text{mut}}_{t+a}$	0.582	0.565	0.516	0.486	0.376	0.405	0.359	0.403
$\hat{f}^{\text{mut}}_{t}/\hat{f}^{\text{mut}}_{t+a}$	0.522	0.525	0.603	0.575	0.647	0.642	0.671	0.646
$\hat{f}^{\text{mut}}_{c}/\hat{f}^{\text{mut}}_{c+g}$	0.432	0.450	0.495	0.511	0.450	0.462	0.388	0.404
$\hat{\sigma}$	3.20	0.918	11.7	0.998	7.26	0.969	5.25	0.339
$\hat{\tau}\hat{\sigma}$	0.0358	0.0217	0.0709	0.0204	0.0558	0.0211	0.0531	0.0185
#parameters	30	31	30	31	30	31	30	31
$\hat{I}_{KL}(\hat{\boldsymbol{\theta}}) \times 10^8$ [b]	129885	126178	144772	126415	180379	169548	233525	222441
ΔAIC [c]	15435.7	14999.0	4801.8	4202.5	670.7	636.0	702.8	674.3
Ratio of substitution rates per codon								
the total base/codon	1.36	1.35	1.53	1.54	1.45	1.48	1.38	1.44
transition/transversion	1.09	1.11	0.803	0.834	1.08	1.13	1.34	1.41
nonsynonymous/synonymous[d]	2.09	2.13	2.48	2.82	2.45	2.65	1.75	1.92
Ratio of substitution rates per codon for $\sigma \rightarrow 0$								
total base/codon	1,0	1.18	1.0	1.38	1.0	1.31	1.0	1.37
transition/transversion	1.49	1.28	1.25	0.944	1.93	1.36	2.35	1.56
nonsynonymous/synonymous[d]	1.12	1.59	0.945	2.13	1.15	1.99	0.767	1.64
Ratio of substitution rates per codon for $w_{ab} = 0$ and $\sigma \rightarrow 0$								
total base/codon	1.0	1.28	1.0	1.59	1.0	1.48	1.0	1.59
transition/transversion	1.31	1.15	0.983	0.830	1.51	1.50	2.15	1.57
nonsynonymous/synonymous[d]	2.57	3.83	2.82	6.53	2.74	1.16	1.84	4.51

[a] In all models, equal codon usage ($\hat{f}^{\text{usage}}_t = \hat{f}^{\text{usage}}_a = \hat{f}^{\text{usage}}_c = \hat{f}^{\text{usage}}_g = 0.25$) is assumed. If the value of a parameter is parenthesized, the parameter is not variable but fixed to the value specified.
[b] $\hat{I}_{KL}(\hat{\boldsymbol{\theta}}) = -(\ell(\hat{\boldsymbol{\theta}})/N + 2.98607330)$ for JTT, $-(\ell(\hat{\boldsymbol{\theta}})/N + 2.97444860)$ for WAG, $-(\ell(\hat{\boldsymbol{\theta}})/N + 2.95801048)$ for cpREV, and $-(\ell(\hat{\boldsymbol{\theta}})/N + 2.85313622)$ for mtREV; see text for details.
[c] $\Delta\text{AIC} \equiv 2N\hat{I}_{KL}(\hat{\boldsymbol{\theta}}) + 2\times$ #parameters with $N \simeq 5919000$ for JTT, $N \approx 1637663$ for WAG, $N \approx 169269$ for cpREV, and $N \approx 137637$ for mtREV; see text for details.
[d] Note that these ratios are not the ratios of the rates per site but per codon; see text for details.

Table S3. ML estimates of the present models with the selective constraints based on the Grantham's and the Miyata's amino acid distances for the 1-PAM substitution matrices of JTT and WAG.

	JTT				WAG			
	Grantham- [a]		Miyata- [a]		Grantham- [a]		Miyata- [a]	
	10	11	10	11	10	11	10	11
$-\hat{w}_0$	(0.0)	(0.0)	(0.0)	(0.0)	(0.0)	(0.0)	(0.0)	(0.0)
$1/\hat{\beta}$	82.0	81.9	1.71	1.82	58.9	65.1	1.28	1.59
$\hat{m}_{[tc][ag]}$	$(\rightarrow 0)$	0.0392	$(\rightarrow 0)$	0.617	$(\rightarrow 0)$	0.353	$(\rightarrow 0)$	1.33
$\hat{m}_{tc\|ag}/\hat{m}_{[tc][ag]}$	2.12	2.09	2.32	1.92	1.49	1.44	1.64	1.40
$\hat{m}_{ag}/\hat{m}_{tc\|ag}$	1.08	1.08	1.05	1.05	1.18	1.17	1.15	1.11
$\hat{m}_{ta}/\hat{m}_{[tc][ag]}$	0.864	0.863	0.925	0.983	0.987	0.938	1.02	1.02
$\hat{m}_{tg}/\hat{m}_{[tc][ag]}$	0.961	0.983	0.922	0.985	0.816	0.907	0.813	0.912
$\hat{m}_{ca}/\hat{m}_{[tc][ag]}$	1.16	1.16	1.26	1.12	1.39	1.32	1.55	1.23
$\hat{f}^{\text{mut}}_{t+a}$	0.582	0.581	0.574	0.543	0.528	0.517	0.499	0.466
$\hat{f}^{\text{mut}}_{t}/\hat{f}^{\text{mut}}_{t+a}$	0.512	0.513	0.513	0.505	0.573	0.562	0.575	0.531
$\hat{f}^{\text{mut}}_{c}/\hat{f}^{\text{mut}}_{c+g}$	0.384	0.385	0.448	0.479	0.412	0.420	0.513	0.541
$\hat{\sigma}$	2.80	2.37	2.98	0.00938	9.00	2.97	9.87	0.00118
$\hat{r}\hat{\sigma}$	0.0330	0.0306	0.0342	0.0147	0.0596	0.0317	0.0632	0.0135
#parameters	30	31	30	31	30	31	30	31
$\hat{I}_{KL}(\hat{\boldsymbol{\theta}}) \times 10^8$ [b]	157835	157281	138419	130721	173694	168463	154639	133347
ΔAIC [c]	18744.5	18680.9	16446.1	15536.8	5749.0	5579.7	5124.9	4429.5
Ratio of substitution rates per codon								
the total base/the total codon	1.35	1.35	1.35	1.34	1.51	1.50	1.51	1.53
transition/transversion	1.04	1.04	1.07	1.10	0.768	0.779	0.791	0.812
nonsynonymous/synonymous [d]	2.21	2.20	2.14	2.18	2.54	2.65	2.53	2.93
Ratio of substitution rates per codon for $\sigma \longrightarrow 0$								
the total base/the total codon	1.0	1.02	1.0	1.33	1.0	1.16	1.0	1.53
transition/transversion	1.33	1.31	1.42	1.10	1.06	0.951	1.17	0.813
nonsynonymous/synonymous [d]	1.22	1.28	1.17	2.17	1.04	1.52	1.02	2.93
Ratio of substitution rates per codon for $w_{ab} = 0$ and $\sigma \longrightarrow 0$								
the total base/the total codon	1.0	1.04	1.0	1.48	1.0	1.26	1.0	1.74
transition/transversion	1.12	1.10	1.21	0.990	0.803	0.771	0.881	0.736
nonsynonymous/synonymous [d]	2.67	2.81	2.63	5.24	2.97	4.20	2.92	8.49

[a] In all models, equal codon usage ($\hat{f}^{\text{usage}}_t = \hat{f}^{\text{usage}}_a = \hat{f}^{\text{usage}}_c = \hat{f}^{\text{usage}}_g = 0.25$) is assumed. If the value of a parameter is parenthesized, the parameter is not variable but fixed to the value specified.

[b] $\hat{I}_{KL}(\hat{\boldsymbol{\theta}}) = -(\ell(\hat{\boldsymbol{\theta}})/N + 2.98607330)$ for JTT, and $-(\ell(\hat{\boldsymbol{\theta}})/N + 2.97444860)$ for WAG; see text for details.

[c] $\Delta\text{AIC} \equiv 2N\hat{I}_{KL}(\hat{\boldsymbol{\theta}}) + 2\times$ #parameters with $N = 5919000$ for JTT, and $N \approx 1637663$ for WAG; see text for details.

[d] Note that these ratios are not the ratios of the rates per site but per codon; see text for details.
