# Supplementary material for: Selective Constraints on Amino Acids Estimated by a Mechanistic Codon Substitution Model with Multiple Nucleotide Changes
Source: PLoS One. 2011 Mar 18;6(3):e17244. doi: 10.1371/journal.pone.0017244 (PMC3060808; doi:10.1371/journal.pone.0017244)
Supplement: Figure S2 — Comparison between various estimates of selective constraint for each amino acid pair The ML estimates of selective constraint on substitutions of each amino acid pair are compared between the models fitted to various empirical substitution matrices. The estimates for multi-step amino acid pairs that belong to the least exchangeable class at least in one of the models are not shown. Plus, circle, and cross marks show the values for one-, two-, and three-step amino acid pairs, respectively. (PDF) [file pone.0017244.s004.pdf]

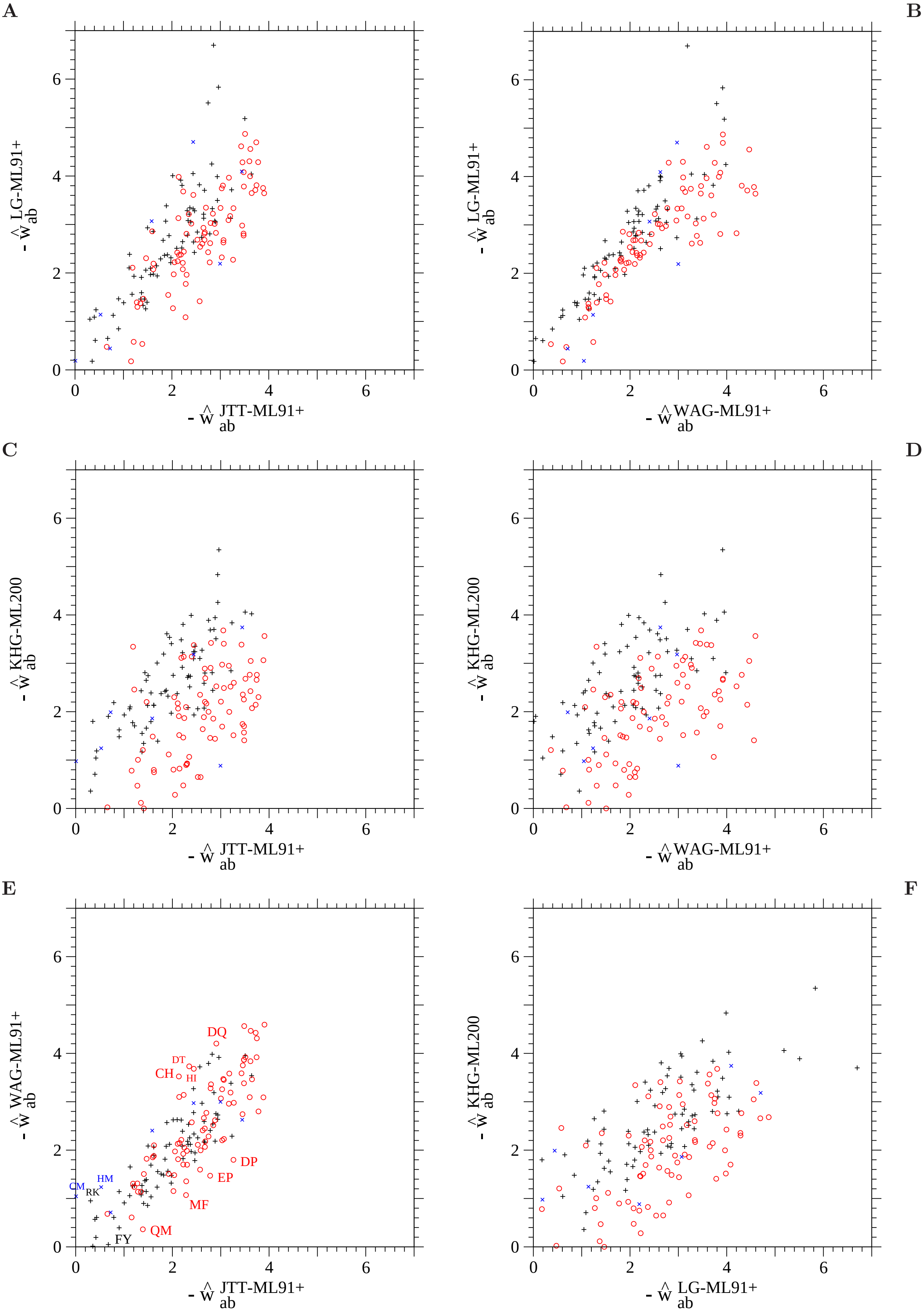

Figure S2. Comparison between various estimates of selective constraint for each amino acid pair The ML estimates of selective constraint on substitutions of each amino acid pair are compared between the models fitted to various empirical substitution matrices. The estimates $\hat{w}_{ab}$ for multi-step amino acid pairs that belong to the least exchangeable class at least in one of the models are not shown. Plus, circle, and cross marks show the values for one-, two-, and three-step amino acid pairs, respectively.
