# Supplementary material for: Selective Constraints on Amino Acids Estimated by a Mechanistic Codon Substitution Model with Multiple Nucleotide Changes
Source: PLoS One. 2011 Mar 18;6(3):e17244. doi: 10.1371/journal.pone.0017244 (PMC3060808; doi:10.1371/journal.pone.0017244)
Supplement: Figure S3 — Selective constraint for each amino acid pair estimated from WAG and from LG. The ML estimate, in (A) and in (B), of selective constraint on substitutions of each amino acid pair in the ML-91+ models fitted to the 1-PAM matrices of WAG and LG is plotted against the mean energy increment due to an amino acid substitution, () defined by Eqs. S1-4, S1-5, and S1-6 in Text S1. The estimates for the least exchangeable class of multi-step amino acid pairs are not shown. Plus, circle, and cross marks show the values for one-, two-, and three-step amino acid pairs, respectively. (PDF) [file pone.0017244.s005.pdf]

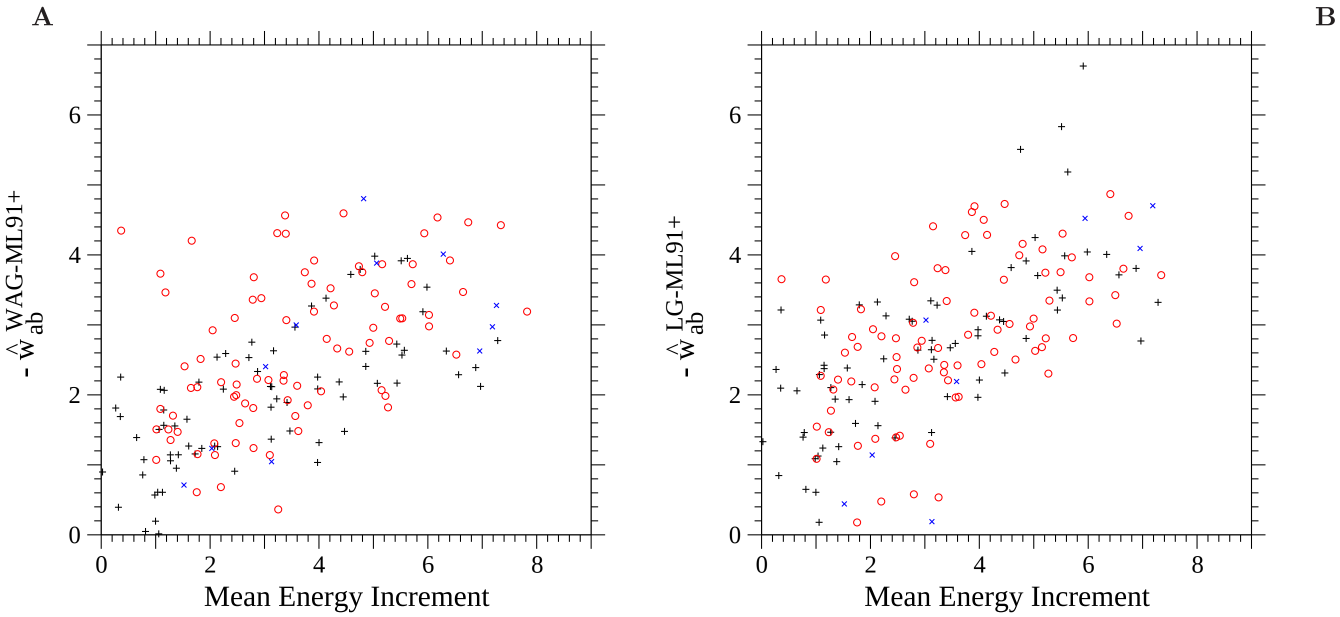

Figure S3. Selective constraint for each amino acid pair estimated from WAG and from LG. The ML estimate, $-\hat{w}_{ab}^{\text{WAG-ML91+}}$ in (A) and $-\hat{w}_{ab}^{\text{LG-ML91+}}$ in (B), of selective constraint on substitutions of each amino acid pair in the ML-91+ models fitted to the 1-PAM matrices of WAG and LG is plotted against the mean energy increment due to an amino acid substitution, $(\Delta\hat{\varepsilon}_{ab}^{c} + \Delta\hat{\varepsilon}_{ab}^{v})$ defined by Eqs. S1-4, S1-5, and S1-6. The estimates $\hat{w}_{ab}$ for the least exchangeable class of multi-step amino acid pairs are not shown. Plus, circle, and cross marks show the values for one-, two-, and three-step amino acid pairs, respectively.
