# Supplementary material for: Selective Constraints on Amino Acids Estimated by a Mechanistic Codon Substitution Model with Multiple Nucleotide Changes
Source: PLoS One. 2011 Mar 18;6(3):e17244. doi: 10.1371/journal.pone.0017244 (PMC3060808; doi:10.1371/journal.pone.0017244)
Supplement: Figure S4 — Comparison of the ML estimates of selective constraint for each amino acid pair between the ML-87 and the ML-91 models. The ML estimate of selective constraint for each single step amino acid pair in the ML-87 model fitted to (A) the 1-PAM JTT matrix or (B) the 1-PAM WAG matrix is plotted against that in the ML-91 model. (PDF) [file pone.0017244.s006.pdf]

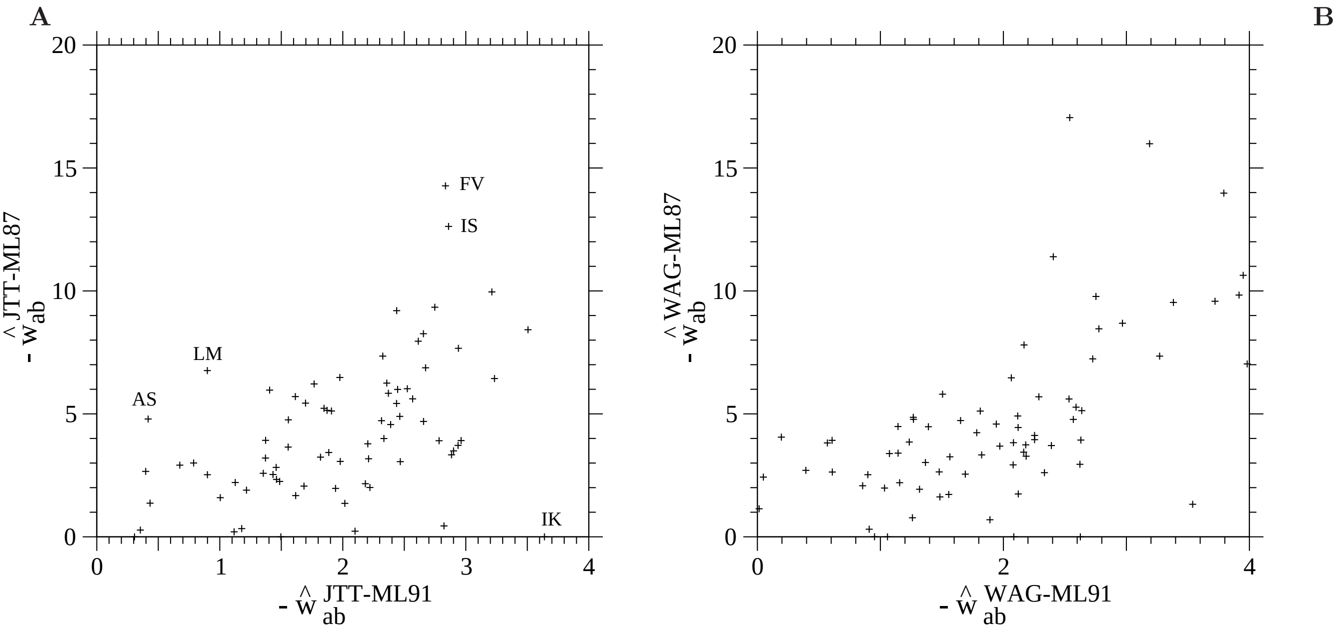

Figure S4. Comparison of the ML estimates of selective constraint for each amino acid pair between the ML-87 and the ML-91 models. The ML estimate of selective constraint for each single step amino acid pair in the ML-87 model fitted to (A) the 1-PAM JTT matrix or (B) the 1-PAM WAG matrix is plotted against that in the ML-91 model.
