# Supplementary material for: Selective Constraints on Amino Acids Estimated by a Mechanistic Codon Substitution Model with Multiple Nucleotide Changes
Source: PLoS One. 2011 Mar 18;6(3):e17244. doi: 10.1371/journal.pone.0017244 (PMC3060808; doi:10.1371/journal.pone.0017244)
Supplement: Figure S5 — Models fitted to each of JTT, WAG, and LG. Each element log- of the log-odds matrix of the model fitted to each empirical substitution matrix is plotted against the log-odds log- calculated from the corresponding empirical substitution matrix. Plus, circle, and cross marks show the log-odds values for one-, two-, and three-step amino acid pairs, respectively. The dotted line in each figure shows the line of equal values between the ordinate and the abscissa. (PDF) [file pone.0017244.s007.pdf]

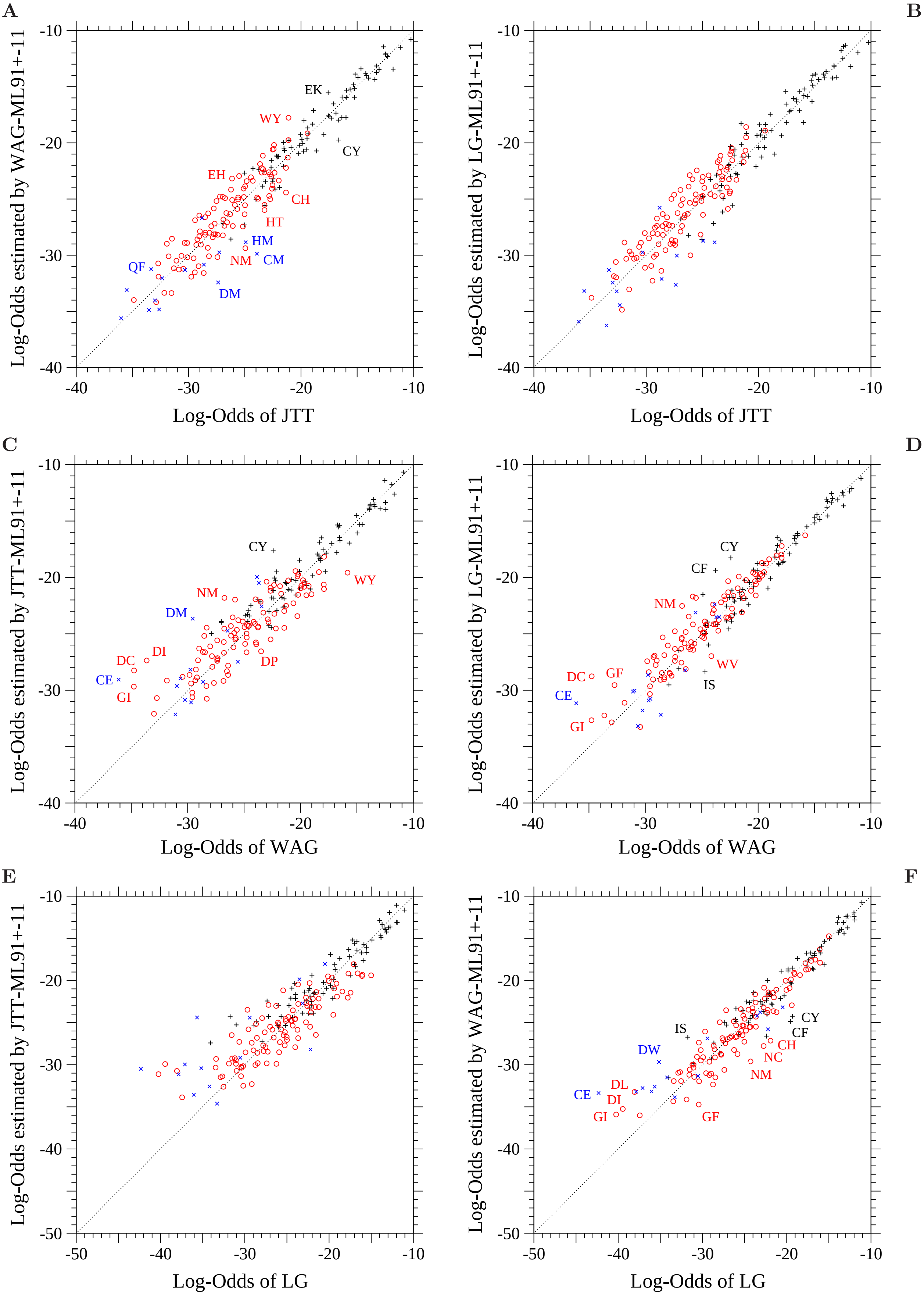

Figure S5. Models fitted to each of JTT, WAG, and LG. Each element log-$O(\langle S\rangle(\hat{\tau},\hat{\sigma}))_{ab}$ of the log-odds matrix of the model fitted to each empirical substitution matrix is plotted against the log-odds log-$O(S^{\text{obs}}(1\ \text{PAM}))_{ab}$ calculated from the corresponding empirical substitution matrix. Plus, circle, and cross marks show the log-odds values for one-, two-, and three-step amino acid pairs, respectively. The dotted line in each figure shows the line of equal values between the ordinate and the abscissa.
