# Supplementary material for: Selective Constraints on Amino Acids Estimated by a Mechanistic Codon Substitution Model with Multiple Nucleotide Changes
Source: PLoS One. 2011 Mar 18;6(3):e17244. doi: 10.1371/journal.pone.0017244 (PMC3060808; doi:10.1371/journal.pone.0017244)
Supplement: Figure S9 — The WAG-ML91+−12 model fitted to the 1-PAM KHG codon substitution matrix. Each element log- of the log-odds matrix corresponding to (A) single, (B) double, and (C) triple nucleotide changes in the WAG-ML91+−12 model fitted to the 1-PAM KHG codon substitution matrix is plotted against the log-odds log- calculated from KHG. Upper triangle, plus, circle, and cross marks show the log-odds values for synonymous pairs and one-, two-, and three-step amino acid pairs, respectively. The dotted line in each figure shows the line of equal values between the ordinate and the abscissa. (PDF) [file pone.0017244.s011.pdf]

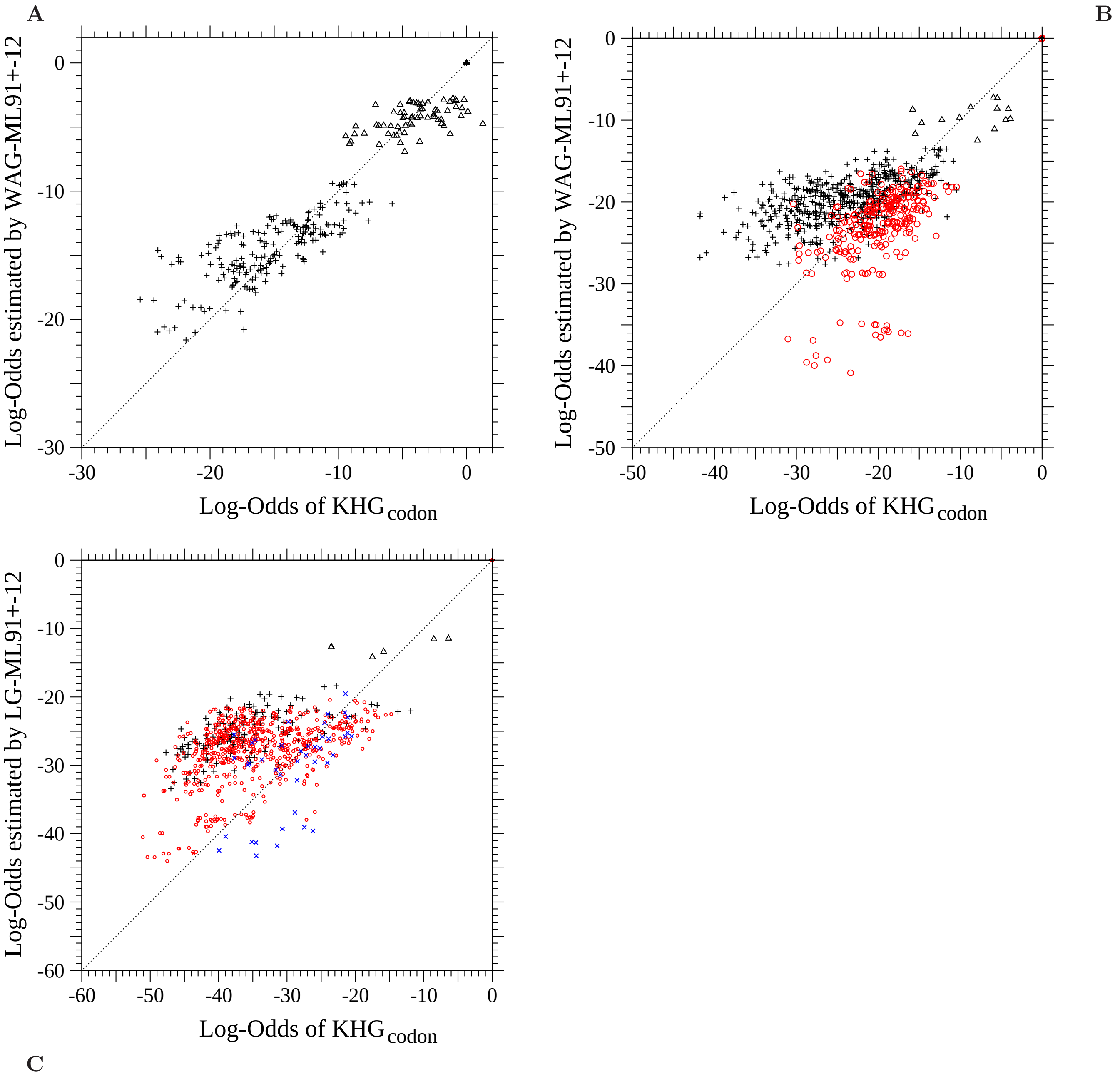

Figure S9. The WAG-ML91+-12 model fitted to the 1-PAM KHG codon substitution matrix. Each element log-$O(\langle S\rangle(\hat{\tau},\hat{\sigma}))_{\mu\nu}$ of the log-odds matrix corresponding to (A) single, (B) double, and (C) triple nucleotide changes in the WAG-ML91+-12 model fitted to the 1-PAM KHG codon substitution matrix is plotted against the log-odds log-$O(S^{\mathrm{KHG}}(1\ \mathrm{PAM}))_{\mu\nu}$ calculated from KHG. Upper triangle, plus, circle, and cross marks show the log-odds values for synonymous pairs and one-, two-, and three-step amino acid pairs, respectively. The dotted line in each figure shows the line of equal values between the ordinate and the abscissa.
