# Supplementary material for: Selective Constraints on Amino Acids Estimated by a Mechanistic Codon Substitution Model with Multiple Nucleotide Changes
Source: PLoS One. 2011 Mar 18;6(3):e17244. doi: 10.1371/journal.pone.0017244 (PMC3060808; doi:10.1371/journal.pone.0017244)
Supplement: Figure S11 — Temporal changes of the eigenvalues and the eigenvectors of the log-odds matrix log- calculated by the ML-91+ model fitted to JTT as a function of sequence identity. In (A), the solid, the broken, and the dotted lines show the temporal changes of the first (), the second (), and the third () principal eigenvalues, respectively. The inner products of the eigenvectors with the eigenvectors of the JTT 20-PAM log-odds matrix, , are shown in (B) for the first principal eigenvector (), in (C) for the second principal eigenvector (), and in (D) for the third principal eigenvector (), by solid lines for , by broken lines for , and by dotted lines for . (PDF) [file pone.0017244.s013.pdf]

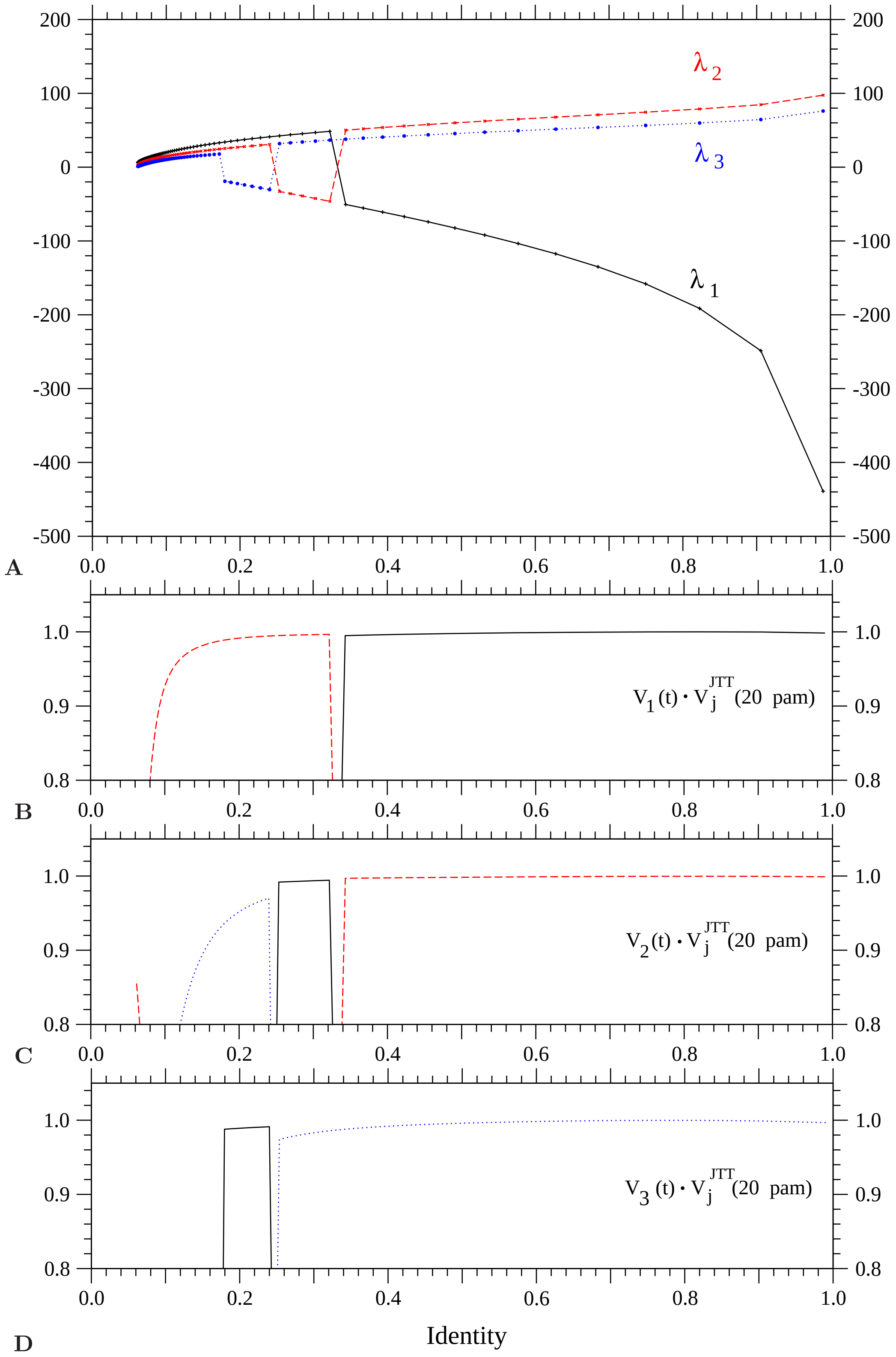

Figure S11. Temporal changes of the eigenvalues and the eigenvectors of the log-odds matrix log-$O(\langle S\rangle(t))$ calculated by the ML-91+ model fitted to JTT as a function of sequence identity. In (A), the solid, the broken, and the dotted lines show the temporal changes of the first (λ_1), the second (λ_2), and the third (λ_3) principal eigenvalues, respectively. The inner products of the eigenvectors with the eigenvectors of the JTT 20-PAM log-odds matrix, $\boldsymbol{V}_i(t) \cdot \boldsymbol{V}_j^{\mathrm{JTT}}$(20-PAM), are shown in (B) for the first principal eigenvector ($i = 1$), in (C) for the second principal eigenvector ($i = 2$), and in (D) for the third principal eigenvector ($i = 3$), by solid lines for $j = 1$, by broken lines for $j = 2$, and by dotted lines for $j = 3$.
