# Supplementary material for: Selective Constraints on Amino Acids Estimated by a Mechanistic Codon Substitution Model with Multiple Nucleotide Changes
Source: PLoS One. 2011 Mar 18;6(3):e17244. doi: 10.1371/journal.pone.0017244 (PMC3060808; doi:10.1371/journal.pone.0017244)
Supplement: Table S1 — ML estimates of the present models without selective constraints on amino acids for the 1-PAM substitution matrices of JTT, WAG, cpREV, and mtREV. (PDF) [file pone.0017244.s014.pdf]

Table S1. ML estimates of the present models without selective constraints on amino acids for the 1-PAM substitution matrices of JTT, WAG, cpREV, and mtREV.

		JTT		WAG		cpREV		mtREV	
		No-Constraints- [a]		No-Constraints- [a]		No-Constraints- [a]		No-Constraints- [a]	
id no.	parameter	1	10	1	10	1	10	1	10
0	$-\hat{w}_0$	(0.0)	(0.0)	(0.0)	(0.0)	(0.0)	(0.0)	(0.0)	(0.0)
1	$1/\hat{\beta}$	(∞)	(∞)	(∞)	(∞)	(∞)	(∞)	(∞)	(∞)
2	$\hat{m}_{[tc][ag]}$	$(\rightarrow 0)$	$\rightarrow 0$	$(\rightarrow 0)$	0.279	$(\rightarrow 0)$	0.0455	$(\rightarrow 0)$	0.0405
3	$\hat{m}_{tc\|ag}/\hat{m}_{[tc][ag]}$	2.16	2.20	1.61	1.54	2.17	2.62	2.32	3.24
4	$\hat{m}_{ag}/\hat{m}_{tc\|ag}$	(1.0)	1.28	(1.0)	1.36	(1.0)	1.50	(1.0)	1.47
5	$\hat{m}_{ta}/\hat{m}_{[tc][ag]}$	(1.0)	0.629	(1.0)	0.687	(1.0)	0.480	(1.0)	0.595
6	$\hat{m}_{tg}/\hat{m}_{[tc][ag]}$	(1.0)	0.708	(1.0)	0.622	(1.0)	0.775	(1.0)	0.373
7	$\hat{m}_{ca}/\hat{m}_{[tc][ag]}$	(1.0)	1.28	(1.0)	1.45	(1.0)	1.64	(1.0)	1.96
8	$\hat{f}^{\text{mut}}_{t+a}$	(0.5)	0.495	(0.5)	0.401	(0.5)	0.279	(0.5)	0.226
9	$\hat{f}^{\text{mut}}_{t}/\hat{f}^{\text{mut}}_{t+a}$	(0.5)	0.486	(0.5)	0.503	(0.5)	0.563	(0.5)	0.583
10	$\hat{f}^{\text{mut}}_{c}/\hat{f}^{\text{mut}}_{c+g}$	(0.5)	0.335	(0.5)	0.354	(0.5)	0.306	(0.5)	0.223
14	$\hat{\sigma}$	$(\rightarrow 0)$	1.76	$(\rightarrow 0)$	1.58	$(\rightarrow 0)$	2.96	$(\rightarrow 0)$	2.46
	$\hat{\tau}\hat{\sigma}$	0.0137	0.0228	0.0136	0.0206	0.0139	0.0296	0.0149	0.0296
	#parameters	21	30	21	30	21	30	21	30
	$\hat{I}_{KL}(\hat{\boldsymbol{\theta}}) \times 10^8$ [b]	729533	207260	1156393	233841	1014962	249448	945289	305500
	ΔAIC [c]	86428.1	24595.5	37917.6	7719.1	3478.0	904.5	2644.1	901.0
	Ratio of substitution rates								
	per codon								
	the total base/codon	1.0	1.30	1.0	1.47	1.0	1.40	1.0	1.35
	transition/transversion	1.13	1.00	0.848	0.752	1.11	1.02	1.24	1.10
	nonsynonymous/synonymous[d]	2.75	4.15	2.84	5.77	2.60	4.91	2.09	3.30
	Ratio of substitution rates								
	per codon for $\sigma \rightarrow 0$								
	the total base/codon	1.0	1.0	1.0	1.21	1.0	1.04	1.0	1.02
	transition/transversion	1.13	1.20	0.848	0.853	1.11	1.43	1.24	1.45
	nonsynonymous/synonymous[d]	2.75	2.83	2.84	4.26	2.60	3.19	2.09	2.08

[a] In all models, equal codon usage ($\hat{f}^{\text{usage}}_t = \hat{f}^{\text{usage}}_a = \hat{f}^{\text{usage}}_c = \hat{f}^{\text{usage}}_g = 0.25$) is assumed. If the value of a parameter is parenthesized, the parameter is not variable but fixed to the value specified.

[b] $\hat{I}_{KL}(\hat{\boldsymbol{\theta}}) = -(\ell(\hat{\boldsymbol{\theta}})/N + 2.98607330)$ for JTT, $-(\ell(\hat{\boldsymbol{\theta}})/N + 2.97444860)$ for WAG, $-(\ell(\hat{\boldsymbol{\theta}})/N + 2.95801048)$ for cpREV, and $-(\ell(\hat{\boldsymbol{\theta}})/N + 2.85313622)$ for mtREV; see text for details.

[c] $\Delta\text{AIC} \equiv 2N\hat{I}_{KL}(\hat{\boldsymbol{\theta}}) + 2\times$ #parameters with $N \simeq 5919000$ for JTT, $N \approx 1637663$ for WAG, $N \approx 169269$ for cpREV and $N \approx 137637$ for mtREV; see text for details.

[d] Note that these ratios are not the ratios of the rates per site but per codon; see text for details.
