# Supplementary material for: Selective Constraints on Amino Acids Estimated by a Mechanistic Codon Substitution Model with Multiple Nucleotide Changes
Source: PLoS One. 2011 Mar 18;6(3):e17244. doi: 10.1371/journal.pone.0017244 (PMC3060808; doi:10.1371/journal.pone.0017244)
Supplement: Table S2 — ML estimates of the present models with the selective constraints based on mean energy increments due to an amino acid substitution (EI) for the 1-PAM substitution matrices of JTT, WAG, cpREV, and mtREV. (PDF) [file pone.0017244.s015.pdf]

Table S2. ML estimates of the present models with the selective constraints based on mean energy increments due to an amino acid substitution (EI) for the 1-PAM substitution matrices of JTT, WAG, cpREV, and mtREV.

	JTT		WAG		cpREV		mtREV	
	EI-10 [a]	EI-11 [a]	EI-10 [a]	EI-11 [a]	EI-10 [a]	EI-11 [a]	EI-10 [a]	EI-11 [a]
$-\hat{w}_0$	(0.0)	(0.0)	(0.0)	(0.0)	(0.0)	(0.0)	(0.0)	(0.0)
$1/\hat{\beta}$	2.50	2.60	1.78	2.14	2.15	2.26	2.14	2.29
$\hat{m}_{[tc][ag]}$	$(\rightarrow 0)$	0.308	$(\rightarrow 0)$	0.916	$(\rightarrow 0)$	0.684	$(\rightarrow 0)$	0.737
$\hat{m}_{tc\|ag}/\hat{m}_{[tc][ag]}$	2.51	2.22	1.82	1.58	2.82	2.24	4.21	3.06
$\hat{m}_{ag}/\hat{m}_{tc\|ag}$	1.01	1.01	1.13	1.10	1.19	1.14	1.05	1.01
$\hat{m}_{ta}/\hat{m}_{[tc][ag]}$	1.02	1.07	1.26	1.22	0.992	1.14	1.48	1.44
$\hat{m}_{tg}/\hat{m}_{[tc][ag]}$	1.06	1.09	0.985	1.01	1.34	1.23	0.792	0.797
$\hat{m}_{ca}/\hat{m}_{[tc][ag]}$	0.937	0.891	1.04	0.949	0.974	0.925	1.17	1.08
$\hat{f}^{\text{mut}}_{t+a}$	0.582	0.565	0.516	0.486	0.376	0.405	0.359	0.403
$\hat{f}^{\text{mut}}_{t}/\hat{f}^{\text{mut}}_{t+a}$	0.522	0.525	0.603	0.575	0.647	0.642	0.671	0.646
$\hat{f}^{\text{mut}}_{c}/\hat{f}^{\text{mut}}_{c+g}$	0.432	0.450	0.495	0.511	0.450	0.462	0.388	0.404
$\hat{\sigma}$	3.20	0.918	11.7	0.998	7.26	0.969	5.25	0.339
$\hat{\tau}\hat{\sigma}$	0.0358	0.0217	0.0709	0.0204	0.0558	0.0211	0.0531	0.0185
#parameters	30	31	30	31	30	31	30	31
$\hat{I}_{KL}(\hat{\boldsymbol{\theta}}) \times 10^8$ [b]	129885	126178	144772	126415	180379	169548	233525	222441
ΔAIC [c]	15435.7	14999.0	4801.8	4202.5	670.7	636.0	702.8	674.3
Ratio of substitution rates per codon								
the total base/codon	1.36	1.35	1.53	1.54	1.45	1.48	1.38	1.44
transition/transversion	1.09	1.11	0.803	0.834	1.08	1.13	1.34	1.41
nonsynonymous/synonymous[d]	2.09	2.13	2.48	2.82	2.45	2.65	1.75	1.92
Ratio of substitution rates per codon for $\sigma \rightarrow 0$								
total base/codon	1,0	1.18	1.0	1.38	1.0	1.31	1.0	1.37
transition/transversion	1.49	1.28	1.25	0.944	1.93	1.36	2.35	1.56
nonsynonymous/synonymous[d]	1.12	1.59	0.945	2.13	1.15	1.99	0.767	1.64
Ratio of substitution rates per codon for $w_{ab} = 0$ and $\sigma \rightarrow 0$								
total base/codon	1.0	1.28	1.0	1.59	1.0	1.48	1.0	1.59
transition/transversion	1.31	1.15	0.983	0.830	1.51	1.50	2.15	1.57
nonsynonymous/synonymous[d]	2.57	3.83	2.82	6.53	2.74	1.16	1.84	4.51

[a] In all models, equal codon usage ($\hat{f}^{\text{usage}}_t = \hat{f}^{\text{usage}}_a = \hat{f}^{\text{usage}}_c = \hat{f}^{\text{usage}}_g = 0.25$) is assumed. If the value of a parameter is parenthesized, the parameter is not variable but fixed to the value specified.
[b] $\hat{I}_{KL}(\hat{\boldsymbol{\theta}}) = -(\ell(\hat{\boldsymbol{\theta}})/N + 2.98607330)$ for JTT, $-(\ell(\hat{\boldsymbol{\theta}})/N + 2.97444860)$ for WAG, $-(\ell(\hat{\boldsymbol{\theta}})/N + 2.95801048)$ for cpREV, and $-(\ell(\hat{\boldsymbol{\theta}})/N + 2.85313622)$ for mtREV; see text for details.
[c] $\Delta\text{AIC} \equiv 2N\hat{I}_{KL}(\hat{\boldsymbol{\theta}}) + 2\times$ #parameters with $N \simeq 5919000$ for JTT, $N \approx 1637663$ for WAG, $N \approx 169269$ for cpREV, and $N \approx 137637$ for mtREV; see text for details.
[d] Note that these ratios are not the ratios of the rates per site but per codon; see text for details.
