# Supplementary material for: Selective Constraints on Amino Acids Estimated by a Mechanistic Codon Substitution Model with Multiple Nucleotide Changes
Source: PLoS One. 2011 Mar 18;6(3):e17244. doi: 10.1371/journal.pone.0017244 (PMC3060808; doi:10.1371/journal.pone.0017244)
Supplement: Table S3 — ML estimates of the present models with the selective constraints based on the Grantham's and the Miyata's amino acid distances for the 1-PAM substitution matrices of JTT and WAG. (PDF) [file pone.0017244.s016.pdf]

Table S3. ML estimates of the present models with the selective constraints based on the Grantham's and the Miyata's amino acid distances for the 1-PAM substitution matrices of JTT and WAG.

	JTT				WAG			
	Grantham- [a]		Miyata- [a]		Grantham- [a]		Miyata- [a]	
	10	11	10	11	10	11	10	11
$-\hat{w}_0$	(0.0)	(0.0)	(0.0)	(0.0)	(0.0)	(0.0)	(0.0)	(0.0)
$1/\hat{\beta}$	82.0	81.9	1.71	1.82	58.9	65.1	1.28	1.59
$\hat{m}_{[tc][ag]}$	$(\rightarrow 0)$	0.0392	$(\rightarrow 0)$	0.617	$(\rightarrow 0)$	0.353	$(\rightarrow 0)$	1.33
$\hat{m}_{tc\|ag}/\hat{m}_{[tc][ag]}$	2.12	2.09	2.32	1.92	1.49	1.44	1.64	1.40
$\hat{m}_{ag}/\hat{m}_{tc\|ag}$	1.08	1.08	1.05	1.05	1.18	1.17	1.15	1.11
$\hat{m}_{ta}/\hat{m}_{[tc][ag]}$	0.864	0.863	0.925	0.983	0.987	0.938	1.02	1.02
$\hat{m}_{tg}/\hat{m}_{[tc][ag]}$	0.961	0.983	0.922	0.985	0.816	0.907	0.813	0.912
$\hat{m}_{ca}/\hat{m}_{[tc][ag]}$	1.16	1.16	1.26	1.12	1.39	1.32	1.55	1.23
$\hat{f}^{\text{mut}}_{t+a}$	0.582	0.581	0.574	0.543	0.528	0.517	0.499	0.466
$\hat{f}^{\text{mut}}_{t}/\hat{f}^{\text{mut}}_{t+a}$	0.512	0.513	0.513	0.505	0.573	0.562	0.575	0.531
$\hat{f}^{\text{mut}}_{c}/\hat{f}^{\text{mut}}_{c+g}$	0.384	0.385	0.448	0.479	0.412	0.420	0.513	0.541
$\hat{\sigma}$	2.80	2.37	2.98	0.00938	9.00	2.97	9.87	0.00118
$\hat{r}\hat{\sigma}$	0.0330	0.0306	0.0342	0.0147	0.0596	0.0317	0.0632	0.0135
#parameters	30	31	30	31	30	31	30	31
$\hat{I}_{KL}(\hat{\boldsymbol{\theta}}) \times 10^8$ [b]	157835	157281	138419	130721	173694	168463	154639	133347
ΔAIC [c]	18744.5	18680.9	16446.1	15536.8	5749.0	5579.7	5124.9	4429.5
Ratio of substitution rates per codon								
the total base/the total codon	1.35	1.35	1.35	1.34	1.51	1.50	1.51	1.53
transition/transversion	1.04	1.04	1.07	1.10	0.768	0.779	0.791	0.812
nonsynonymous/synonymous[d]	2.21	2.20	2.14	2.18	2.54	2.65	2.53	2.93
Ratio of substitution rates per codon for $\sigma \rightarrow 0$								
the total base/the total codon	1.0	1.02	1.0	1.33	1.0	1.16	1.0	1.53
transition/transversion	1.33	1.31	1.42	1.10	1.06	0.951	1.17	0.813
nonsynonymous/synonymous[d]	1.22	1.28	1.17	2.17	1.04	1.52	1.02	2.93
Ratio of substitution rates per codon for $w_{ab} = 0$ and $\sigma \rightarrow 0$								
the total base/the total codon	1.0	1.04	1.0	1.48	1.0	1.26	1.0	1.74
transition/transversion	1.12	1.10	1.21	0.990	0.803	0.771	0.881	0.736
nonsynonymous/synonymous[d]	2.67	2.81	2.63	5.24	2.97	4.20	2.92	8.49

[a] In all models, equal codon usage ($\hat{f}^{\text{usage}}_t = \hat{f}^{\text{usage}}_a = \hat{f}^{\text{usage}}_c = \hat{f}^{\text{usage}}_g = 0.25$) is assumed. If the value of a parameter is parenthesized, the parameter is not variable but fixed to the value specified.

[b] $\hat{I}_{KL}(\hat{\boldsymbol{\theta}}) = -(\ell(\hat{\boldsymbol{\theta}})/N + 2.98607330)$ for JTT, and $-(\ell(\hat{\boldsymbol{\theta}})/N + 2.97444860)$ for WAG; see text for details.

[c] $\Delta\text{AIC} \equiv 2N\hat{I}_{KL}(\hat{\boldsymbol{\theta}}) + 2\times$ #parameters with $N = 5919000$ for JTT, and $N \approx 1637663$ for WAG; see text for details.

[d] Note that these ratios are not the ratios of the rates per site but per codon; see text for details.
